# Megaevolutionary dynamics and the timing of evolutionary innovation in reptiles

**DOI:** 10.1038/s41467-020-17190-9

**Published:** 2020-07-03

**Authors:** Tiago R. Simões, Oksana Vernygora, Michael W. Caldwell, Stephanie E. Pierce

**Affiliations:** 1 Department of Organismic and Evolutionary Biology & Museum of Comparative Zoology, Harvard University, Cambridge, MA 02138 USA; 2 Department of Biological Sciences, University of Alberta, Edmonton, AB T6G 2E9 Canada; 3 Department of Earth and Atmospheric Sciences, University of Alberta, Edmonton, AB T6G 2E9 Canada

**Keywords:** Evolution, Palaeontology, Phylogenetics

## Abstract

The origin of phenotypic diversity among higher clades is one of the most fundamental topics in evolutionary biology. However, due to methodological challenges, few studies have assessed rates of evolution and phenotypic disparity across broad scales of time to understand the evolutionary dynamics behind the origin and early evolution of new clades. Here, we provide a total-evidence dating approach to this problem in diapsid reptiles. We find major chronological gaps between periods of high evolutionary rates (phenotypic and molecular) and expansion in phenotypic disparity in reptile evolution. Importantly, many instances of accelerated phenotypic evolution are detected at the origin of major clades and body plans, but not concurrent with previously proposed periods of adaptive radiation. Furthermore, strongly heterogenic rates of evolution mark the acquisition of similarly adapted functional types, and the origin of snakes is marked by the highest rates of phenotypic evolution in diapsid history.

## Abstract (web summary)

The dynamics of how major clades and body plans arise and evolve in deep time remain poorly understood. Here, Simões et al. report major time lags between phases of rapid phenotypic change at the origin of major reptile lineages and periods of fast molecular change and adaptive radiation.

## Introduction

Understanding the origin of new clades and body plans in the history of life represents one of the biggest challenges in evolutionary biology^1^ and has been a focus of intensive investigation for decades. Unraveling megaevolutionary dynamics^2^ among higher clades provides the key to illuminate major shifts in the pace of phenotypic and molecular evolution, the relationship between rates of phenotypic change and phenotypic diversity, and how those variables may be influenced by major events, such as mass extinctions. For instance, it has been long recognized that new animal body plans appear suddenly in the geological record, suggesting that major transformations leading to key new phenotypes seemingly occur at short and localized time periods^1–5^. These punctuated evolutionary patterns have prompted the idea that the origin and early evolution of major clades and phenotypic innovations would be necessarily associated with high rates of phenotypic evolution and expansion in phenotypic disparity in deep time^1–5^. However, our ability to decipher the patterns and processes of evolution has been largely constrained by the methodological tools and the amount of data available to address those topics. In most instances, researchers were limited to localized assessments of faunal change across stratigraphic intervals, mostly applied to hard-bodied invertebrates and mammalian teeth, which are more abundant in the fossil record [e.g., refs. ^3,4,6^.

Only in recent years have quantitative tools been developed to rigorously assess important megaevolutionary patterns across broad time scales in evolutionary paleobiology. As a result, new studies using both relaxed clocks and phylogenetic comparative methods have found high rates of morphological evolution at the origin of major clades, including the early evolution of birds, arthropods and crown placental mammals^7–9^. Fast evolutionary rates during putative periods of adaptive radiations following mass extinctions have also been recovered, such as the radiation of birds^10^ and placental mammals^8^ after the Cretaceous–Palaeogene mass extinction, and archosaurs after the Permian–Triassic mass extinction (PTME)^11^. Overall, these results support long-established predictions that major phenotypic innovations are marked by fast evolutionary rates, and expansion in phenotypic disparity and taxonomic diversity, concentrated at the origin of new major clades during periods of adaptive radiations^2–5,12^— when ecological opportunities (stemming from the invasion of new environments, recovery after mass extinctions, or the development of key new phenotypic traits) would trigger the expansion of a clade into new adaptive zones^3,13,14^. Once niches are occupied, phenotypic disparity stabilizes and species diversification and evolutionary rates decrease and stabilize at lower levels^3,13–15^.

Nevertheless, there have been important recent challenges to this classical view of the timing and rate of phenotypic change. It has been suggested that the pattern of fast rates of evolution at the origin of major clades (early burst model) does not seem universal as it was not recovered during the origin and initial radiation of some major groups, such as teleost fishes or echinoids^16,17^. Importantly, very few studies include information from the fossil record and thus cannot fully examine such megaevolutionary events at sufficiently large scales of time in order to be able to fully comprehend the expected long-term dynamics bracketing time frames before and after mass extinctions, or at the origin of major clades (for notable exceptions, see refs. ^15,17–19^). When observed at sufficiently broad scales of time, new patterns emerge generating alternative models to the classical ideas described above: episodic radiations, when seemingly early bursts at the origin of major clades actually represent smaller episodic events of rapid evolution throughout evolutionary history (e.g., in echinoids)^17^; and constructive radiations, when there is a long chronological gap between the origin of clades and new body plans (associated with high evolutionary rates and phenotypic disparity), and the actual period of taxonomic diversification (e.g., in early metazoans)^1,20^. Therefore, the timing and rate of major phenotypic revolutions generating new clades and body plans and their occurrence at times of adaptive radiations remains an open question.

Additionally, there has been a decades long debate on the molecular drivers of fast phenotypic innovation, characteristic of adaptive radiations and other periods of fast phenotypic change. Until recently, most theories were dominated by the idea that rapid phenotypic change had to be linked to fast changes in protein coding genes. This concept undergirds macromutation (saltationism) theory’s “hopeful monsters” from early geneticists^2^ and the founder effect theory of Mayr^12^, the latter being subsequently co-opted by Eldredge and Gould^4,5^ to explain sudden morphological changes in the fossil record. However, more recent advances in genomics have suggested that the major drivers of fast phenotypic evolution may not be linked to protein coding genes at all, but concentrated in regulatory regions^21^. If protein coding nuclear and mitochondrial markers (more traditionally analyzed in phylogenetics) are in fact not the primary drivers of phenotypic diversification, then phenotypic rates of evolution would be expected to be decoupled from overall measures of evolutionary rates at protein coding loci—although we note that individual analysis of all coding loci would inevitably reveal some level of correlation between a few coding loci (e.g., from de novo mutations) and changes in the phenotype. Yet, only a handful of studies have utilized available morphological and molecular data to address the relationships between genetic and phenotypic evolutionary rates at broad taxonomic scales or deep geological time [e.g.,ref. ^8^] to test whether reconstructed periods of rate shifts in protein coding loci correspond to the periods of fast phenotypic evolution.

Here, we explore megaevolutionary dynamics on phenotypic and molecular evolution during two fundamental periods of reptile evolution: (i) the origin and early diversification of the major lineages of diapsid reptiles (lizards, snakes, tuataras, turtles, archosaurs, marine reptiles, among others) during the Permian and Triassic periods, and (ii) the origin and evolution of lepidosaurs (lizards, snakes and tuataras) from the Jurassic to the present. The first provides answers concerning the origin of some of the most fundamental body plans in reptile evolution, as well as the impact of the largest mass extinction event in the history of complex life (the PTME) on early reptile evolution. The second reveals fundamental clues towards the evolution of one of the most successful vertebrate lineages on Earth today, comprising over 10,500 different species^22^. Our major questions for these two chronological and taxonomic categories include: What are the major deep time evolutionary patterns concerning evolutionary rates and phenotypic disparity? Do most periods of expansion of evolutionary rates and/or morphological disparity occur at the origin of major clades and new body plans? Which megaevolutionary model (e.g., adaptive vs. constructive radiations) better characterizes the patterns observed in early diapsid and lepidosaur evolution? Can we identify concurrent periods of fast morphological and molecular evolutionary rates in the evolution of lepidosaurs? Our findings indicate a complex evolutionary scenario in which different parts of the diapsid and lepidosaur tree of life are better explained by alternative megaevolutionary models. Unexpectedly, we also find that phenotypic novelties converging on similar functions evolved at very distinct rates of evolution.

## Results

### Trees, divergence times and rates of evolution

We expanded upon our recently published phylogenetic dataset of early evolving diapsid reptiles and lepidosaurs (fossils and living)^23^, by adding new data on extant lizards and snakes to inform both phenotypic and molecular components of the tree. To estimate evolutionary rates in a calibrated evolutionary tree, we integrated both phenotypic and molecular data using total-evidence dating (TED). This is a powerful approach in which tree topology, divergence times and phenotypic and molecular evolutionary rates are jointly estimated. To account for potential variations in estimates of divergence times and evolutionary rates due to different software implementations, we conducted analyses using the software MrBayes^24^ and the BEAST2 evolutionary package^25^. However, the BEAST packages lack diversity sampling strategies, which is known to potentially overestimate divergence times with TED^26,27^. Our results with BEAST2 had relatively older divergence times compared to MrBayes, especially among older nodes, which we attribute to this factor. For all our trees, see Supplementary Figs. 1–14 and Supplementary Data 2 and 3.

In our analyses we found evidence for deep root attraction (DRA)^28^ that, when corrected (following ref. ^28^), increased the precision for divergence times in MrBayes (Supplementary Figs. 4, 5, 14), and were also in much greater agreement with the fossil record—e.g. the divergence time for the diapsid-captorhinid split at the earliest Pennsylvanian (322 Mya), thus being close to the age of oldest known diapsid reptiles from the Late Pennsylvanian^29^. In contrast, even in analyses in which we tried to correct for DRA in BEAST2, the median age for the diapsid-captorhinid split was placed at the latest Devonian close to the Devonian-Carboniferous boundary (ca. 40 million years older), a time at which the first known tetrapods were diversifying onto land^30^, and thus, considerably more inconsistent with the fossil record (Supplementary Figs. 6, 7). Overestimated divergence times are likely to affect estimates of evolutionary rates by extending chronological branch lengths (i.e. branch duration) and reducing overall precision for divergence times (Supplementary Fig. 14, Supplementary Table 2). Therefore, our results and conclusions are primarily driven from the posterior tree estimates obtained from MrBayes (results from BEAST2 are provided in Supplementary Figs. 6, 7, 9, 12, 13).

Our initial non-clock Bayesian inference results with lepidosaurs indicate strong topological similarity between our molecular tree and a recent phylogenomic study of lepidosaurs^31^, especially concerning the paraphyly of amphisbaenians in both instances. Amphisbaenian paraphyly was also obtained by analyzing phenotypic data only. In each case, clades usually retrieved as the sister group to amphisbaenians (lacertids for molecular data and dibamids for phenotypic data) were found within “amphisbaenians” (Supplementary Figs. 1–3). Contrary to the results recovered using a previous version of this dataset^23^, we find considerable agreement concerning early diapsid relationships between total evidence non-clock and clock trees with results from MrBayes (Supplementary Figs. 3–5).

**Fig. 1:**
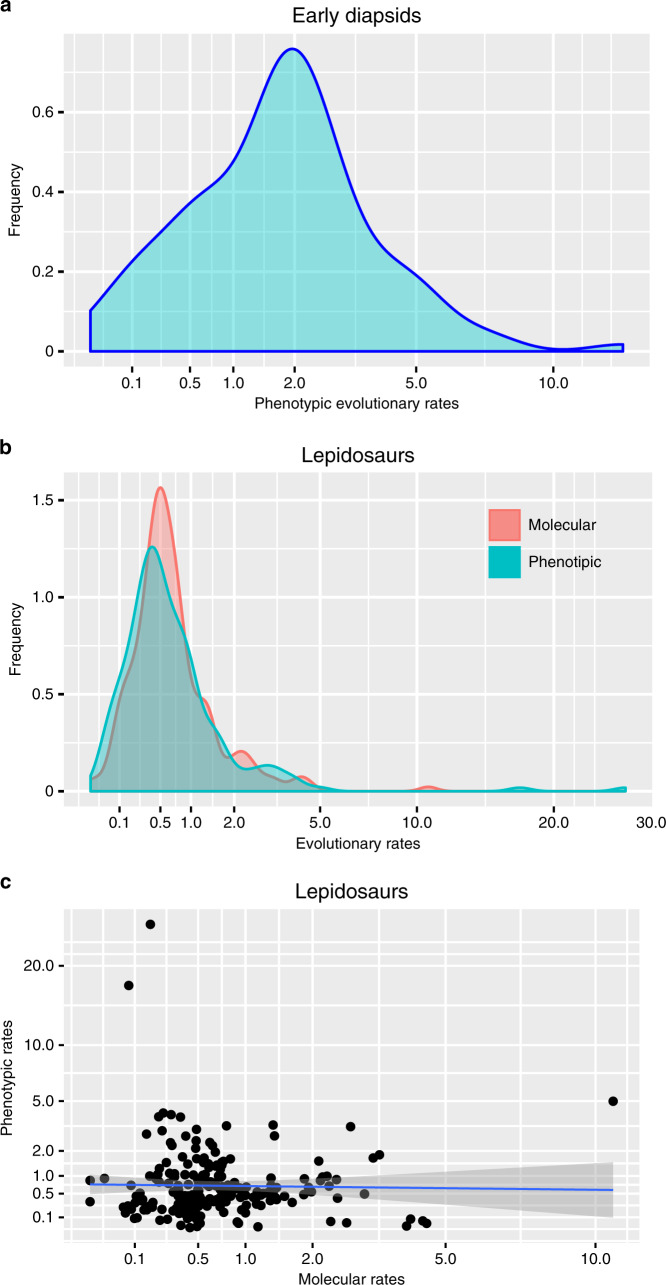
Distribution of phenotypic and molecular relative evolutionary rates in different reptile groups. Rates are relative to the mean posterior estimate of base of the clock rate value (= 0.00345 substitutions per character per myr). **a** distribution of phenotypic rates among early evolving diapsid lineages (median=1.9; mean=2.1). **b** distribution of phenotypic (median=0.51; mean=1.0) and molecular rates (median=0.54; mean=0.83) among lepidosaurs. **c** linear regression between phenotypic and molecular rates (log-transformed) among lepidosaurs (R-squared: 0.0001425; *p*-value: 0.8615; two-sided). *n* = 109 (**a**) and *n* = 211 (**b**, **c**) for molecular and phenotypic evolutionary rates pooled from all unique bipartitions from the posterior trees. Shaded grey area = 95% CI. Source data are provided as a Source Data file.

**Fig. 2:**
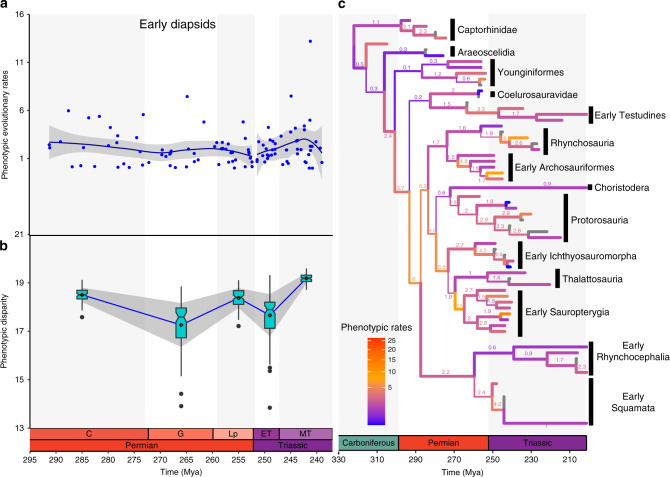
Relative phenotypic evolutionary rates and disparity through time in early diapsid reptiles. Branch widths proportional to posterior probabilities for clades stemming from the respective branches. **a** phenotypic rates among the major early evolving diapsid reptile lineages from the Early Permian to the Middle Triassic. Grey area represents 95% CI around LOESS regression line segmented by geological time bins. Carboniferous rates are not considered here due to low sample size and extremely broad confidence intervals. **b** phenotypic disparity in early diapsids through time. Box plots represent distribution of 100 bootstraps at each time bin. Lower bound = 1st quartile (Q1); upper bound = 3rd quartile (Q3); notching = median; diamond = mean; minima = Q1-1.5xIQR; maxima = Q3 + 1.5xIQR; outliers = dots. Grey area represents one SD around the means (connected by blue trendline). *n* = 109 (**a**, **b**) evolutionary rates pooled from all unique bipartitions from the posterior trees. **c** phenotypic rates of evolution in reptiles plotted on the time-calibrated maximum compatible tree from Mr. Bayes. For full tree see Supplementary Figs. 5, 10.

**Fig. 3:**
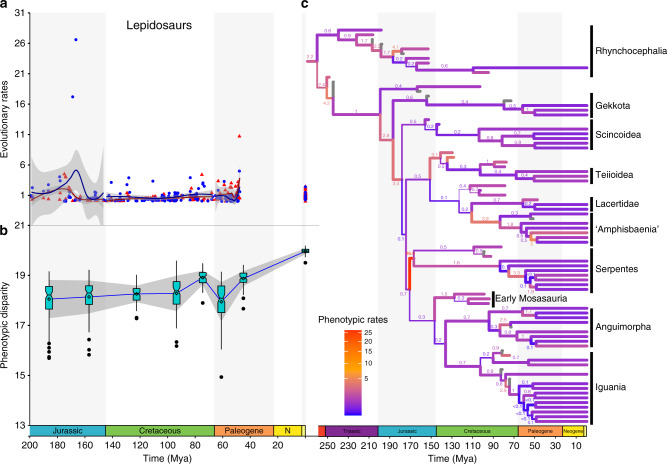
Relative phenotypic and molecular evolutionary rates and disparity through time in lepidosaurs. Branch widths proportional to posterior probabilities for clades stemming from the respective branches. **a** phenotypic (blue) and molecular (red) rates among the major lepidosaur lineages from the Jurassic to the present time. Grey area represents 95% CI around LOESS regression line segmented by geological time bins. Triassic rates are not considered here due to low sample size and extremely large confidence intervals. **b** phenotypic disparity in early diapsids through time. Box plots represent distribution of 100 bootstraps at each time bin. Lower bound = 1st quartile (Q1); upper bound = 3rd quartile (Q3); notching = median; diamond = mean; minima = Q1-1.5xIQR; maxima = Q3 + 1.5xIQR; outliers = dots. Grey area represents 95% CI around LOESS regression line segmented by geological time bins (phenotypic = dark blue and molecular = dark red). *n* = 211 (**a**, **b**) for molecular and phenotypic evolutionary rates pooled from all unique bipartitions from the posterior trees. **c** phenotypic rates of evolution in reptiles plotted on the time-calibrated maximum compatible tree from Mr. Bayes. For full tree see Supplementary Figs. 5, 10.

**Fig. 4:**
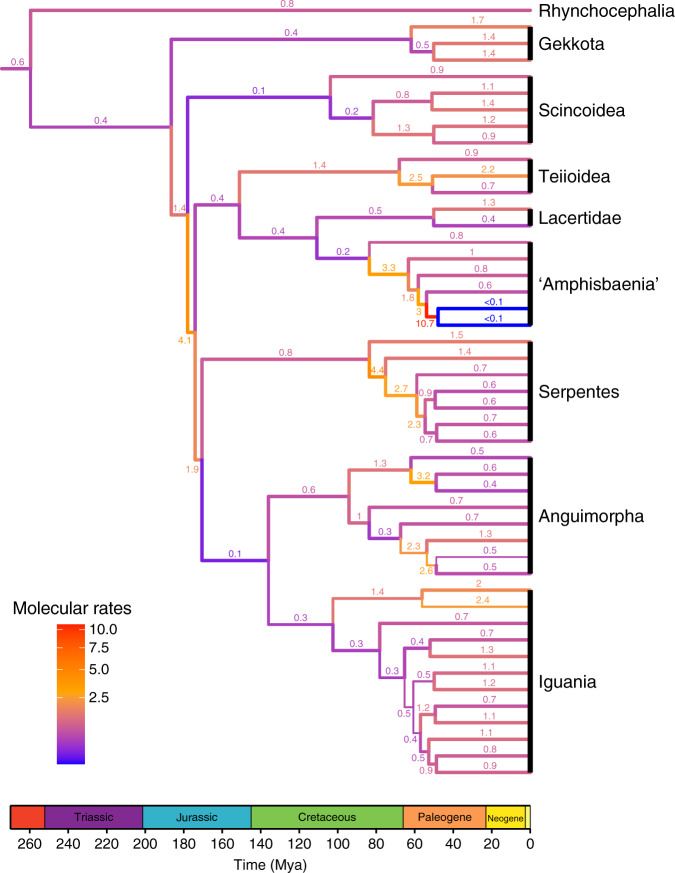
Relative molecular rates of evolution in lepidosaurs. Branch widths proportional to posterior probabilities for clades stemming from the respective branches. Molecular rates are plotted for all sampled extant taxa and internal branches until their most recent common ancestor. For full tree and rate values see Supplementary Figs. 5, 11.

In all of our results from total-evidence relaxed clocks, inferred relative rates of phenotypic evolution have their medians and means similar to each other across time bins, with modal, median and mean values ~2.0 for early evolving diapsid lineages during the Permian up to the end of the Middle Triassic (Figs. 1a, 2). In lepidosaurs, phenotypic and molecular rates have similar distributions, and median, mean and modal values between 0.3 and 1 (Figs. 1b, 3). Further, in lepidosaurs there is no detectable correlation between phenotypic and molecular rates (Fig. 1c), demonstrating a strong decoupling between both rates in the data available herein (supporting the utilization of separate clocks for phenotypic and molecular data). Among the periods of elevated rates of molecular evolution, only the early part of the Jurassic is coincident with relatively fast rates of phenotypic evolution. But even in this case, the branches exhibiting fast phenotypic change are not the same undergoing fast molecular change (Figs. 3, 4).

### Evolutionary patterns in early diapsids across time

When observed across time, phenotypic rates of evolution in early diapsids are consistently accelerated relative to the base of rates (above one—see Methods—and well above mean, median and modal values) during most of the Permian, indicating elevated rates of evolution at the origin of the major lineages of diapsid reptiles (Fig. 2a, c, Supplementary Table 3). This is coupled with relatively high rates of phenotypic disparity (Fig. 2b, Supplementary Tables 6, 11), although this disparity drops during the Guadalupian, and increases again during the Late Permian. Given the data presently available, it is difficult to determine precisely when most of the disparity was lost during the Guadalupian, but the early Guadalupian also depicts some of the lowest phenotypic evolutionary rates during the Permian, which then increase at the Guadalupian–Lopingian transition. Those results suggest that some level of extinction followed by recovery of phenotypic diversity happened during the Permian, and thus during early diapsid history. Taxonomic diversity of terrestrial non-flying tetrapods has been recently demonstrated to also drop during the Guadalupian, followed by an increase by the end of the Permian^32^. These results support recent hypotheses that early diapsid reptiles were affected by the more recently discovered Guadalupian mass extinction^33^. However, the relative increase in evolutionary rates at the Guadalupian–Lopingian boundary is much milder compared to the increase in phenotypic disparity, thus deviating from the expected signal from a recovery event from a mass extinction. We note that the amount of data available for this particular time slot concerning early reptiles in the present dataset may not be enough to fully capture a shifting evolutionary rate regime at a high scale of resolution. Future addition of Middle Permian reptiles to our dataset will provide a stronger assessment of shifts in evolutionary rates and its relationship to shifts in phenotypic disparity and taxonomic diversity and assessing the impact of the Guadalupian mass extinction in early reptiles.

Phenotypic rates decrease at the Permian–Triassic boundary, but rapidly increase during the first few million years of the Triassic, reaching their peak at the Middle Triassic, and subsequently start to decrease at the end of the Middle Triassic (Fig. 2a). Phenotypic disparity drops during the Early Triassic but recovers quickly and expands above pre-extinction levels by the Middle Triassic (Fig. 2b). These changes in evolutionary rates and disparity levels match the expected patterns of an adaptive radiation in the aftermath of mass extinctions, in which the occupation of available ecological niches is associated with an expansion of phenotypic disparity and high evolutionary rates^3^. This is further supported by the well-documented increase in the number of diapsid species and clades during the Triassic^30^, which occupied several distinct habitats and adaptive zones^34,35^.

We note, however, that there are large fluctuations of relative phenotypic evolutionary rates between 260 and 237 million years ago, and great variability between and within clades (Fig. 2a, c; Supplementary Figs. 10, 12, 15, 19, Supplementary Table 3). As a consequence, there is a general trend towards increasing phenotypic rates between the PTME and the Middle Triassic (~252–243 Mya—Fig. 2a, c)—mean relative phenotypic rates jumped from 1.557 in the Lopingian to 1. 868 in the Early Triassic and 2.629 in the Middle Triassic. This trend is not shared to the same extent by all lineages however, with standard deviations for those same periods being quite large: 1.1, 0.9 and 2.6, respectively. This across-lineage rate variation declines after 243 Mya, resulting in a nonsignificant difference of mean rate values between the Early Triassic and Middle Triassic (Supplementary Table 8). On the other hand, disparity patterns are less variable among all lineages (considerably smaller standard deviations), resulting in significant differences of disparity mean values between time bins (Supplementary Table 11). Therefore, despite the overall fast rate of recovery of morphological disparity in the aftermath of the end-Permian extinction, the reconquering of morphospace happened at a very distinct pace among early diapsid lineages. More specifically, fast relative phenotypic change marked the early evolution of rhynchosaurs, early archosauriforms, early squamates, and some (but not all) lineages of marine reptiles, such as early sauropterygians (especially placodonts) and early ichthyosauromorphs—all with relative rates well above the overall mean and median values for the Early and Middle Triassic (Fig. 2c, Supplementary Table 3). Other lineages, such as early choristoderes, thalattosaurs, most early lepidosauromorphs, and early turtles present relative rates mostly >1 (accelerated relative to the base clock rate), but close to or below the estimated means for relative rates in the Early and Middle Triassic. This result indicates the latter reptile groups were not radiating (at least not to the same extent) as were other early diapsid lineages we tested. This result may also explain the much lower taxonomic diversity throughout the entire Triassic identified for those latter lineages, all of which have only a handful of species recognized from the entire Triassic (with the possible exception of thalattosaurs).

### Evolutionary patterns in lepidosaurs across time

In lepidosaurs, the beginning of the Jurassic marks the divergence of some of the deepest branches among major squamates clades, with elevated rates of phenotypic and molecular evolution at the origin of those clades as well as moderately high rates of phenotypic disparity (Fig. 3, Supplementary Tables 4, 5). In general, those rates are lower compared to the rates observed at the origin and radiation of the major diapsid lineages during the Permian and Triassic. One major exception to the later trend, however, is the extremely high rate of phenotypic evolution at the origin of snakes. The branch leading to snakes is inferred to have the highest rates of phenotypic evolution among all the lineages of diapsid reptiles studied here (Figs. 2, 3). High phenotypic rates during the early evolution of snakes were also recently found by another study based on extant snake taxa in a lepidosaur cranial shape dataset^36^. Elevated rates of phenotypic evolution suggest an additional potential explanation for the difficulty in estimating the phylogenetic placement of snakes among squamates using phenotypic data only, besides the issue of multiple independent cases of the reduction of limbs among lizards. Interestingly, molecular rates of evolution on the lineage leading to snakes are not as elevated, although they become higher within snakes, especially when compared to molecular rates of other squamate lineages (Figs. 3, 4).

Both phenotypic and molecular evolutionary rates among lepidosaurs stabilize during the Cretaceous, lying closer to modal levels (Fig. 3a, b), and reach another peak in inferred phenotypic and molecular rates in the latest Cretaceous (Campanian–Maastrichtian). Those rates in the latest Cretaceous are significantly higher compared to the low rate levels observed in the Early Cretaceous, but there is no visibly distinct trend in rate increase during the Cretaceous (Fig. 3), and they are also not significantly different from other time bins preceding and succeeding the latest Cretaceous (Supplementary Tables 9, 10). Phenotypic disparity increased slowly but steadily throughout the Cretaceous, reaching its highest peak during the Mesozoic at the end of the Cretaceous (Fig. 3b, Supplementary Tables 7,12). This gradual increase in phenotypic disparity is supported by the fossil record, as the Late Cretaceous sees the first appearance and subsequent increase in phenotypic disparity and taxonomic diversity of the aquatically adapted mosasaurians^37^, appearance of the oldest preserved legged snakes^38^, along with the appearance of a large diversity of many crown group lizards in the Campanian and Maastrichtian of Mongolia^39^.

Lepidosaur phenotypic disparity drops following the Cretaceous–Paleogene mass extinction (Fig. 3b). Disparity, as well as phenotypic and molecular evolutionary rates (Figs. 3, 4) remain relatively low during the Paleocene, with disparity increasing again during the Eocene, reaching pre-extinction levels. Phenotypic evolutionary rates do not see an equivalent increase to those observed for phenotypic disparity, although molecular rates are higher during the middle Eocene compared to earlier parts of the Paleogene. We lack sufficient data to estimate evolutionary rates during the Neogene, but rates among extant species remain relatively low while disparity is the highest in the history of lepidosaurs. The latter is supported by considering that squamates (essentially almost all of the extant diversity of lepidosaurs) comprise more than 10,500 living species, a level of taxonomic diversity that is inferred to be the highest in the history of the group^32^, including ecologically diverse forms inhabiting almost any environment outside of the polar circles.

## Discussion

Adaptive radiations are traditionally believed to be responsible for the origin of most of Earth’s taxonomic and phenotypic diversity, usually associated with the first stages of the evolution of major clades^2,3^. However, in our results, we only detected one instance in the history of early diapsid and lepidosaurian reptiles that matches the expectations of adaptive radiations (*sensu*^3,13,14^) at broad taxonomic and deep time scales (see also Introduction). This is the recovery from the aftermath of the PTME during the Triassic by some early diapsid lineages (archosauriforms, rhynchosaurs, sauropterygians and ichthyosauriforms). The patterns detected here for some early evolving diapsid reptiles matches that of the recovery of other vertebrate and invertebrate lineages^34,35,40,41^ in the aftermath of the PTME. As previously hypothesized^34,35,40^, it is suggested that the emergence of ecological opportunities following the PTME—elimination of competitors (e.g. early synapsid clades) and exploration of new habitats and lifestyles (e.g., marine environments)—allowed some surviving reptile lineages to radiate in the Triassic. This highlights the capacitating role of ecological opportunities in the aftermath of mass extinctions towards driving adaptive radiations^13^.

At other periods of time we see patterns that are better explained by alternative models of evolution. For instance, we observe an important chronological lag between the time of origin (and initial phenotypic radiation) of the major diapsid lineages during the Permian and their later taxonomic diversification^32^. This deviates from the classical model of adaptive radiation and instead matches the expectations of a pattern of early disparification without taxonomic richness (*sensu*^42^) that is quickly followed by periods of loss and recovery of phenotypic disparity during the Late Permian. This example illustrates how the fast evolution of key features (i.e. evolutionary innovation—defined here as the appearance or subsequent change of morphological structures inferred to be of high adaptive value to explore a new ecological niche) resulting in fast evolutionary rates and large morphological disparity, does not necessarily coincide with periods of adaptive radiation. In the case of early diapsid reptiles, particular aspects of their initial evolution (most importantly, taxonomic diversity and expansion into new adaptive zones) was most likely limited by the worldwide distribution of early synapsids clades that were direct competitors to early reptiles in many terrestrial ecosystems during the Permian^34,35,40^. It is only after the demise of several synapsid lineages at the PTME that ecological opportunities matched with a new period of morphological innovation led to an adaptive radiation among some early evolving lineages of diapsid reptiles.

Among lepidosaurs, we detected some of the highest rates of evolution during the early part of the Jurassic, at the time of diversification of some of the deepest branches in squamate evolution and the origin of important new body plans (e.g. snakes), but which is also marked by low lepidosaurian taxonomic diversity^32^. In contrast, both phenotypic and molecular evolutionary rates were relatively stable and at low levels during most of the Cretaceous, the period where most of the first examples of modern lineages of squamates show up in the fossil record: the peak of Mesozoic taxonomic richness is reached at the end of the Cretaceous^32,43^. Additionally, overall phenotypic disparity slowly increased between the Jurassic and the end of the Cretaceous, indicating a slow and steady buildup of phenotypic space. Such a substantial gap of 100 million years between initially high rates of evolution and the much later acquisition of taxonomic richness^32,43^, associated with a continuous construction of morphospace, is better characterized by the more recently proposed constructive radiation model^1^, which predicts that the emergence of phenotypic novelties predate their taxonomic diversification by several millions of years. A major similar example is the fast evolution of phenotypic novelties and the exploration of morphospace during the early evolution of metazoans at the Cambrian explosion, generating many clades with few species, but with actual taxonomic diversification occurring much later in the history of animals^1,20^.

Differently from diapsid reptiles during the Permian, it is less clear why phenotypic innovation in lepidosaurs during the Jurassic (especially the origin of snakes) did not lead to a noticeable taxonomic diversification, especially among squamates. Possible reasons include competitors for resources or predators limiting ecological opportunities to early snakes and other Jurassic squamates. However, our current knowledge of the squamate fossil record in the Jurassic is still limited to a few well-preserved articulated specimens^44,45^, and most early snakes are known only from highly fragmentary remains^45^, thus limiting our ability to understand their ecological roles and interactions with other clades. As our understanding of the deployment of the snake body plan and habitat occupation has recently been advancing based on new fossil evidence^46,47^, we predict that future findings will soon be able to reveal fundamental clues on the potential factors enabling phenotypic innovation decoupled from taxonomic radiation during early snake evolution.

In all of our results, phylogenetic branches with the highest phenotypic rates are frequently those within the early branches of newly evolving clades with distinct new adaptive anatomical features characterizing new body plans (e.g., the emergence of turtles, marine reptiles, archosaurs and snakes [Figs. 2, 3]). Early fast evolving branches in the history of new major clades were long predicted (termed “tachytelic” lineages^3^), but were supposed to occur at periods of adaptive radiation. However, many such bursts in phenotypic evolution are not concentrated at specific time frames, such as the ones usually identified as adaptive radiations (e.g., the aftermath of the Permian–Triassic mass extinction^32,40,48^). Instead, we detected multiple bursts of phenotypic evolution throughout reptile history, as similarly observed during echinoid evolution^17^.

High rates are also observed during the acquisition of unique body plans that represent “failed” evolutionary experiments (lineages that did not reach high levels of diversification and went extinct soon after their origin), such as placodonts during early sauropterygian evolution in the Early Triassic (Fig. 2). The causes behind the failure of such lineages to diversify in the face of ecological opportunity (represented by new phenotypes and vacant ecological niches after the PTME) are hard to test in the fossil record. One proposed explanation is that, despite the availability of ecological opportunities, some clades simply lacked the ability to evolve fast enough, and could not adapt to new adaptive zones^13,49^. However, the observed fast phenotypic rates in those short-living Triassic diapsid lineages suggest otherwise. More parsimonious explanations include the misidentification of ecological opportunity—at the local level, various surviving lineages could be exploring the same resources (e.g., other marine reptiles adapted to near-shore environments co-inhabiting and competing with placodonts). It is also possible that competitors adapted to similar environments occupied vacant niches earlier and were already diversifying and utilizing available resources^13^.

**Fig. 5:**
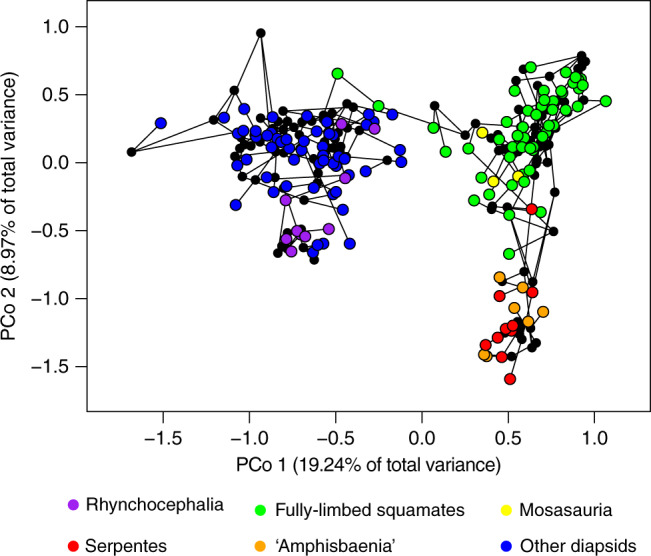
Phylomorphospace of early diapsid reptiles and lepidosaurs. The first two axes of phenotypic variation among principal coordinates. Early diapsid reptiles occupy a distinct region of the morphospace from lepidosaurs, which in turn, have rhynchocephalians, non-serpentiform squamates, and serpentiform squamates (snakes and amphisbaenians) occupying different regions of the morphospace defined by PCo 1 and 2.

Surprisingly, clades with very similar functional adaptations exhibit radically different rates of phenotypic evolution. For instance, protective/armored morphotypes (turtles and placodonts), aquatic morphotypes (ichthyosaurs, thalattosaurs, eosauropterygians and mosasaurians), and serpentiform morphotypes (snakes and amphisbaenians) show highly distinct rates of evolution in their early history (Figs. 2, 3), during the acquisition of their respective key phenotypic innovations. While the fastest evolving branch in early turtle evolution has rates up to 2.15 times faster than median values for overall rates of phenotypic evolution, the fastest branch in placodonts is 8.3 times faster than the median for early diapsids. The most dramatic example is represented by the extremely similar (but evolutionary convergent) morphology of amphisbaenians and snakes (Fig. 5), which have extremely different rates of evolution at their origin (5 times faster than median values for lepidosaurs in amphisbaenians vs 34.1 times faster in snakes). We note that, although usually characterized by the limblessness of its extant representatives, early snakes still retained partially developed limbs^38^, and many of the character changes contributing to those fast evolutionary rates relate to changes in the skull of both snakes and amphisbaenians, not limb evolution. Indeed, fast rates of skull shape evolution on the branch leading to snakes were found recently by Watanabe et al. ^36^.

Contrary to the fast changes observed on phenotypic evolutionary rates, most deep nodes in lepidosaur evolution are defined by comparatively slower rates of molecular evolution (Figs. 1c, 3, 4). Interestingly, rates of molecular evolution are quite low on the branch leading to snakes and other clades that show high levels of phenotypic evolution. Among the few empirical studies comparing phenotypic and molecular rates of evolution across broad time scales, a similar pattern is observed in mammals (despite using different methodologies from us), with molecular rates kept at relatively low levels during the early evolution of placental mammals^8^. Those results indicate that the structural protein coding sequences tested herein for lepidosaurs, and also in the mammalian study, do not seem to have any detectable correlation to the substantial and fast phenotypic changes observed at the origin of new body plans in diapsid reptile and mammalian evolution.

Although early theories on the genetic drivers of major phenotypic changes invoked revolutions in frequencies of protein coding genes^3,5^, genomic studies have revealed that substantial phenotypic change appears to be mediated by changes on cis-regulatory elements (CREs), and not by developmental gene duplication, or functional protein changes by mutations in coding sequences^21,50,51^. Therefore, our results from rates on protein coding sequences compared to rates of phenotypic evolution support the hypothesis that most genomic change associated with major phenotypic transitions in lepidosaurs (and possibly extinct reptile lineages that cannot be sampled for molecular data) is not located on protein coding genes, and might, instead, be located on conserved regulatory regions, as recently detected in the evolution of paleognathous birds^50^. Although outside the scope of the present study, we consider that the assessment of rates of evolution on conserved regulatory regions and a greater sampling of protein coding genes, to be a fundamental next step in the investigation of the genomic basis for fast phenotypic change in reptiles.

Our results indicating exceptionally high phenotypic evolutionary rates at the origin of snakes further suggest that snakes not only possess a distinctive morphology within reptiles^52^, but also that the first steps towards the acquisition of the snake body plan were extremely fast. The available (and future) genomic data on snakes may well retain critical information for understanding the drivers and processes behind phenotypic innovation in lepidosaurs. Potential drivers may be represented by factors such as transposable elements (TEs). TEs can be found in large numbers in protein coding, intronic and regulatory sequences, and may become exapted to novel functions, including regulation of gene expression within CREs in mammals^53^. The situation is even more dramatic in squamates, in which *Hox* gene clusters (which usually lack TEs and are conserved in most vertebrates to preserve the regulation of organismal development^54^) have an unparalleled accumulation of TEs compared to other vertebrates^53,55,56^. TEs also have a role in the expansion of the number of microsatellites by microsatellite seeding^57^. In conformity with their large number of TEs, squamates have undergone microsatellite seeding during their evolution, and as a result, squamates have the highest abundance of microsatellites among vertebrates, with snakes, in particular, having the highest microsatellite content among eukaryotes^57^. It is therefore possible that the unusually high number of TEs and microsatellites in squamates (snakes in particular), and their subsequent exaptation to novel functions in the genome (both in protein coding and regulatory regions) may be one of the fundamental drivers of phenotypic innovation in squamates^53,55^, explaining the exceptional rates of evolution observed in snakes.

The patterns and processes governing the origin of major clades and new morphotypes across the tree of life remain poorly understood. Our study illuminates some of those aspects by assessing megaevolutionary dynamics over broad taxonomic and chronological scales in reptile evolution. Although the evolution of most early diapsid reptiles shows the classic signatures of an adaptive radiation following the PTME as previously hypothesized^34,35,40^, we also detected many fast evolving lineages and expansion of phenotypic disparity spread across various time frames, thus being better explained by alternative megaevolutionary models, such as constructive^1,20^ and episodic radiations^17^. Additionally, we find that at least some early diapsid lineages (choristoderes, thalattosaurs, and some early lepidosauromorphs and turtles) already had evolutionary rates closer to background levels during the Early and Middle Triassic. Importantly, we also find considerable rate heterogeneity during the early evolution of functionally similar body plans. How generalizable these patterns are across other major metazoan lineages remains to be determined. Collectively, our findings suggest a considerably more complex scenario concerning the evolution of reptiles in deep time than previously thought.

## Methods

### Co-estimating trees and macroevolutionary parameters

Most paleobiological studies assessing rates of phenotypic evolution have utilized maximum parsimony inferred phylogenetic trees for an a posteriori estimate of changes along the branches of the tree [e.g., refs. ^7,58,59^]. Those estimates have provided valuable insights into evolutionary dynamics in deep time, especially concerning fossil lineages, and to the understanding of detailed patterns of phenotypic change. However, an essential limitation of this approach is how to timescale the tree and the fact that frequently used parsimony trees minimize the number of changes along the branches, with both factors directly affecting evolutionary rate estimates^60^. The integration of both phenotypic and molecular clocks in total evidence dating provides a powerful approach in which tree topology, divergence times, and phenotypic and molecular evolutionary rates are jointly estimated, thus circumventing those limitations (we refer the interested reader on the foundations of clock analyses to an existing comprehensive literature on the topic, e.g. refs. ^60–63^). Further, estimates of divergence times and evolutionary rates can be averaged across the posterior sample of trees so that estimates take phylogenetic uncertainty into consideration. When those parameter estimates are taken directly from the Bayesian summary/consensus trees, those trees can be constructed based on the product of posterior probabilities or by selecting the posterior tree with highest posterior probability. This procedure yields fully resolved trees upon which macroevolutionary parameters can be inferred without ambiguity as is often the case with consensus trees derived from maximum parsimony (e.g., choosing between *acctran* vs. *deltran* approaches at polytomic nodes, or arbitrarily resolving polytomies). Additionally, different types of relaxed-clock models are available (representing essentially distinct modes of evolution)^64,65^, and can be tested in order to determine which model has the better fit to the dataset, thus allowing an essential simultaneous consideration of tempo and mode towards estimating evolutionary relationships and rates of evolution.

### Morphological and molecular datasets

Here we updated the recently published diapsid-squamate dataset of Simões et al.^23^ in order to expand the representation of extant taxa, which are informative on both morphological and molecular data. Twelve additional taxa were added to this dataset: nine extant species (the snakes *Rena humilis, Afrotyphlops punctatus, Python regius* and *Lichanura trivirgata*, the amphisbaenians *Amphisbaena alba, Trogonophis wiegmanni*, and three additional limbed lizards, *Tupinambis teguixin, Celestus stenurus* and *Varanus albigularis*) and three fossil taxa (*Pleurosaurus goldfussi, Gobiderma pulchrum* and *Cryptolacerta hassiaca*). Morphological data were collected for the additional taxa based on personal observations (by T.R.S.) and molecular data from the nine extant taxa were added to the molecular component of this dataset^23^. Three taxa that operate as wildcards in the present dataset, as identified in a previous study using the RogueNaRok algorithm^23,66^, were removed for the present analyses, namely *Paliguana whitei*, *Palaeagama vielhaueri* and *Pamelina polonica*. Increased taxon sampling and rogue taxon removal resulted in considerable improvement on resolution and convergency of results on early diapsid relationships between non-clock and clock trees (see Results)—as expected based on recent simulations data^67^.

The molecular dataset for the selected coding regions were obtained from GenBank (Supplementary Table 1, Supplementary Data 2). For *Python regius*, for which molecular data were not available, we used sequences of congeneric species, *P. molurus*. Sequences were aligned in MAFFT 7.245^68^ online server using the global alignment strategy with iterative refinement and consistency scores (G-INS-i). Molecular sequences from all extant taxa were analyzed for the best partitioning scheme and model of evolution using PartitionFinder2^69^ under Bayesian information criterion (BIC).

Our major goal was to focus taxonomic sampling on (i) having representatives from all major lineages of early diapsid reptiles and lepidosaurs (fossils); (ii) periods of time encompassing previously proposed periods of adaptive radiation; and (iii) taxa representing the earliest stages on the evolution of major lineages. Aspects (i) and (iii) are especially important for retrieving good phylogenetic hypotheses and accurate diversification times. The final dataset consists of 347 morphological characters (including autapomorphies) sampled across 138 species (80 lepidosaurs and 58 non-lepidosaurian early diapsids) and 16 nuclear and mitochondrial loci (11,532 sites) for 47 extant representatives of lepidosaurs. Importantly, all morphological characters included here were carefully revised and compared to each other following available guidelines^70,71^ to avoid issues stemming from logical or biological dependencies among them; these are known to potentially create biases owing to character coding strategies and impact morphological tree topologies. The final multiple sequence alignment for the molecular dataset was concatenated and visually examined in Mesquite v. 3.04 [http://mesquiteproject.org]. As shown in the Results, the molecular data provide a result similar to that of a recently published phylogenomic analysis^31^ (see Supplementary Fig. 1); this suggests that despite the much smaller size of our molecular dataset, phylogenetic signal in our molecular dataset corresponds to that of recent and much more extensive molecular datasets.

### Bayesian inference analyses

Both non-clock and clock based Bayesian inference analyses were conducted using MrBayes v. 3.2.6^24^ and the BEAST2 package^25^ using high performance computing resources made available through Compute Canada. Molecular partitions were analyzed using the models of evolution obtained from PartitionFinder2^69^ (see dataset), and the morphological partition was analyzed with the Mkv model^72^.

### Time-calibrated relaxed-clock Bayesian inference analyses

We implemented total-evidence-dating (TED) using the fossilized birth-death tree model with sampled ancestors (FBD-SA), under relaxed-clock models in MrBayes v.3.2.6^27,73^—100 million generations, with four independent runs with six chains each, and a gamma prior of rate variation across characters. We conducted the same analysis using the BEAST2 package^25^, with four independent runs, also with a gamma prior of rate variation across characters. To ensure that each independent single chain run in BEAST2 reached stationarity, we increased length of each analysis to 200 million generations. Runs were sampled every 500 generation with the initial 55% of samples removed as “burn-in”. We provided an informative prior to the base of the clock rate based on the previous non-clock analysis: the median value for tree height in substitutions from posterior trees divided by the age of the tree based on the median of the distribution for the root prior: 23.8582/325.45 = 0.0733, in natural log scale = −2.61308. We chose to use the exponent of the mean to provide a broad standard deviation: *e*^0.0733^ = 1.076053. The vast majority of our calibrations were based on tip-dating, which accounts for the uncertainty in the placement of fossil taxa and avoids the issue of constraining priors on taxon relationships when implementing bound estimates for node-based age calibrations^73^. The range of the stratigraphic occurrence of the fossils used for tip-dating here were used to inform the uniform prior distributions on the age of those same fossil tips (thus allowing for uncertainty on the age of the fossils).

It has been demonstrated that using tip dates only can contribute to unrealistically older divergence time estimates for some clades^74,75^. It is also expected that using tip dates only may underestimate divergence times for some clades if the fossil taxa used for tip-dating are much younger than the oldest known appearance of that particular clade. Therefore, for the clades for which we lacked some of the oldest known fossils in our analysis, and for which there is overwhelming support in the literature (and in all our other analyses) regarding their monophyletism, and for which the age of the oldest known fossil is well-established, we employed node age calibrations with a soft minimum age. These clades and calibrations are as follows: Serpentes: based on *Eophis underwoodi* (Bathonian, Middle Jurassic—UK)^45^ → 168.3–166.1 Mya (166.1,168.3)^76^; Rhynchocephalia: based on cf. *Diphyodontosaurus* (Ladinian, Middle Triassic—Germany)^77^ → 241.5–237 Mya (237,241.5)^76^; and Choristodera: lower bound for the node age based on *Cteniogenys* sp. (Bathonian, Middle Jurassic—UK)^78^ (166.1–168.3), which is already older than the oldest taxa sampled in this analysis. However, differently from a previous analysis of this dataset^23^, we provided a much wider and uninformative upper bound to take into account the uncertainty regarding the age of the oldest choristoderes, which may span as far back as the Middle Triassic (ca. 240 Mya)^79,80^. Further, in the previous analysis of this dataset, Captorhinidae also had its node age calibrated based on *Euconcordia* (Stephanian of Europe [equivalent to the Kasimovian], Late Pennsylvanian, Carboniferous—Kansas, USA). However, *Euconcordia* may actually not nest with captorhinids (TRS, unpublished data), and therefore, we preferred to be cautious and not constrain the captorhinid node. Further, mosasaurian squamates were previously calibrated using tip dates only. However, although the oldest phylogenetically informative mosasaurians personally assessed by us are Cenomanian in age, including the three species used herein, the oldest known records of mosasaurian reptiles date as far back as the earliest Cretaceous, represented by *Kaganaias hakusanensis* from the Hauterivian from Japan^81^ and an isolated vertebra from the Barremian of Spain^82^. Therefore, in order to provide a more reliable upper bound for the divergence time of mosasaurians, we calibrated that node based on those records→132.9–125 Mya (125,132 .9). All node calibrations were given a soft upper bound, which gives a low (but non-zero) likelihood of the age being older than the upper bound value.

Convergence of independent runs was assessed using: average standard deviation of split frequencies (ASDSF ~ 0.01), potential scale reduction factors [PSRF ≈ 1 for all parameters] and effective sample size (ESS) for each parameter was greater than 200 for MrBayes. Independent BEAST runs were combined using LogCombiner v2.5.1 (available with the BEAST2 package) and checked for stationarity and convergence in Tracer v. 1.7.1 [http://beast.bio.ed.ac.uk/Tracer]. The ESS value of each parameter was greater than 200 and ASDSF < 0.01.

### Testing for the best fitting clock prior

Distinct clock models allow for different assumptions regarding the predominant tempo and mode of evolution. While strict clocks presume constant rates of evolution across lineages, relaxed-clock models allow for changes in the rate of evolution among lineages. For instance, relaxed clocks include models where rates at each branch in a phylogeny are drawn independently and identically from an underlying rate distribution (uncorrelated clocks), to others where the rate at a particular branch is dependent on the rates on the neighbouring branches (autocorrelated clocks)—see refs. ^64,65^ for model comparisons. Therefore, in uncorrelated clocks rates are free to change more dramatically among neighbouring branches, resulting in shorter branch lengths and higher rates than autocorrelated clock models^73^, and thus reflecting a more punctuated model of evolution compared to the more gradualistic model represented by autocorrelated rates^65^.

In order to detect the most appropriate clock models, we used Bayes factors (BFs) applying model fitting analyses using the stepping-stone sampling strategy to assess the marginal model likelihoods^83^ for each clock model for the current dataset [50 steps for 100 million generations in MrBayes and 100 million generations in BEAST2 (two runs each)]. We tested between strict clock models and relaxed-clock models. Relaxed-clock models were further tested for linked clock models (where morphological and molecular partitions share the same clock, and therefore variations on the rate of evolution) and unlinked clock models, allowing for the clock rates to vary independently among the morphological and molecular partitions of the dataset. In MrBayes the analysis reached stationarity phase and we found a considerably stronger fit for relaxed-clock models against a strict clock model (BF > 2000), and a stronger fit (BF > 400) for unlinked clock models, thus supporting the treatment of morphological and molecular rates independently. The results are expected given the broad scale of the present dataset, which is inclusive of several reptile families sampled over the last 300 million years and characters from multiple regions of the phenotype and genotype. Finally, an independent gamma rate (IGR) unlinked relaxed-clock model was favoured relative to the autocorrelated clock model (BF = 150), indicating the data supports a model allowing more disparate shifts in evolutionary rates across lineages. The stepping-stone analyses conducted in BEAST2 ran for 100 million generations, with some of the longest runs (using random local clocks) taking 37 days to complete in a computer cluster, and yet they failed to reach the stationarity phase. The large taxonomic sampling of our dataset (which increases computational time exponentially) compared to most other total evidence datasets analysed under BEAST2 indicate that the sheer size of this dataset prevents a reasonable assessment of marginal likelihoods independently from the ones performed under Mr. Bayes. Therefore, for subsequent analyses using BEAST2 we implemented the two uncorrelated relaxed-clock models available in BEAST2 (lognormal and exponential), given the much stronger fit of uncorrelated relaxed-clock models over other clock models in MrBayes. Further, relaxed-clock implementations can recover homogeneous rates of evolution when the best fit model is supposed to be clock-like^84^, indicating relaxed-clock models can fit a variety of different evolutionary scenarios. The lognormally distributed rates of evolution inferred from MrBayes (Fig. 1) suggests the lognormal clock model from BEAST2 is to be preferred, and the results from BEAST2 further indicate greater agreement in tree topology between different software is obtained with a lognormal relaxed clock in BEAST2.

### Divergence time estimates and evolutionary rates

Divergence time estimates using relaxed clocks are highly dependent on the tree prior choices, such as the prior on the strategy for sampling extant taxa. For instance, whereas a “random” sampling strategy assumes that all taxa are sampled randomly, a “diversity” sampling strategy is more appropriate when extant taxa are sampled in a way to maximize diversity (as performed herein) and fossils are sampled randomly^27,73^. A diversity sampling tree prior is implemented in MrBayes v. 3.2.6, but it is not yet available on BEAST2. Accounting for diversity sampling impacts tree priors^26^, affecting divergence time precision and accuracy^27,73^. The results from our initial relaxed-clock analyses show considerably (and unreasonably) older divergence times from the trees using BEAST2 compared to MrBayes (tens of millions of years older). Considering the main prior choices were the same between the two software packages, we attribute the much older divergence times in BEAST2 to not accounting for diversity sampling in total evidence analyses, as already demonstrated by previous studies^27,73^. Additionally, factors such as vague priors, limitations on currently available models of morphological evolution as well as conflict between the morphological and molecular signal may result in pushing divergence times further back in time (exceptionally long ghost lineages), especially among the deepest nodes on broad scale phylogenies, contributing to the phenomenon of deep root attraction (DRA)^28^. It is possible to minimize this impact by providing informative priors that decrease the likelihood of long ghost lineages, such as modelling higher diversification rates or a low extinction probability. This correction for DRA could potentially provide a tool for correcting the overestimation of divergence times in BEAST2, as reported above. Following Ronquist et al.^28^, we implemented one of those strategies (specifically, giving higher probabilities of low extinction by placing a Beta (1100) prior on the turnover probability) to assess its impact on divergence times on the analyses conducted on both MrBayes and BEAST2.

Implementing this strategy highly increased the precision of divergence times among the oldest nodes on the summary tree from MrBayes, and also brought divergence times for the oldest nodes on the tree into much greater agreement with the fossil record (Supplementary Figs. 4, 5, 14; Supplementary Table 2). For instance, in the analysis with no DRA correction average variance of divergence times among the 50 oldest nodes taken from the posterior trees was 143.07 million years (myr), whereas it was 58.88 myr among the 50 youngest nodes (excluding extant nodes); in the analysis with DRA correction those respective values decreased to 25.3 myr and 16.49 myr (see also ranges of 95%HPD between those analyses in Supplementary Figs. 4, 5). Therefore, we used the results from the DRA corrected analyses to report divergence times and evolutionary rates. Notably, even accounting for low extinction probability to reduce DRA in our analyses using BEAST2, we noticed no visible difference in divergence times among the oldest nodes. Divergence times were still considerably older (frequently 10–20 million years older) among intermediary and older nodes in the maximum clade credibility (MCC) tree compared to the summary tree from MrBayes (Supplementary Figs. 6, 7). This suggests that informative tree priors are not enough to avoid overestimating divergence times when diversity sampling is not taken into account, at least in BEAST2. Branch length estimates and tree calibration invariably impact estimates of absolute rate values and correlating rates with specific periods of time in the geological record is affected when divergence times are biased. As a result, our main results report only the trees from Mr. Bayes, where divergence times are not being overestimated by DRA. The results from BEAST2 are reported from trees with stronger convergence on clade composition and distribution of rate values with the summary tree from MrBayes (from the lognormal relaxed clock), but we urge caution on the interpretation of those results.

The mean posterior estimate for the base of the clock (evolutionary) rate value for both phenotypic and molecular characters (a generalized absolute background rate) was obtained from the log files and analyzed with in in Tracer v. 1.7.1, representing numbers of substitutions per character per million years. A second parameter estimated in Bayesian relaxed clocks is the variance on the base of the clock rate, which represents the deviations from the clock rate parameter at every branch of the phylogeny, thus representing a relative clock (evolutionary) rate estimate. Therefore, relative rate values at each branch of the evolutionary tree with values >1 represent accelerating rates, whereas values <1 represent decelerating rate values^24^. Further, we used resulting estimates of relative evolutionary rates pooled from all the nodes from the posterior trees as reported by MrBayes, as well as from the summary tree (maximum compatibility tree (MCT)) from MrBayes. The trendlines constructed from the former thus represent an average taken from rate estimates from distinct clade compositions from the posterior trees (Figs. 2, 3), therefore having the advantage of taking phylogenetic uncertainty into consideration. Importantly, the rates taken from the summary tree have similar trendlines and rate variation over time (Supplementary Fig. 15), therefore revealing the same general pattern and indicating the data in the summary tree provides a good representation of rate change over time.

Our reported summary trees were calculated with standard output tree procedures available in MrBayes and BEAST2: the tree maximizing the product of the posterior probabilities taken from all the trees among the sampled posterior trees, which generates the MCC tree from BEAST2 and the MCT from MrBayes.

### Dataset adaptation for morphological disparity analyses

Phylogenetic morphological characters provide a large number of variables that can be easily utilized for morphospace analysis and have been implemented in a large variety of studies on different taxonomic groups. Importantly, discrete phylogenetic characters can easily capture the disparate morphological variation that is observed among higher taxa (as observed in broad scale phylogenies, such as in the present dataset) [e.g. ref. ^85^]. Even among smaller scale phylogenies, morphospace patterns inferred from discrete phylogenetic characters result in morphospace patterns very similar to the ones inferred from morphometric data^86^. Additionally, phylogenetic characters can capture morphological variation from several aspects of the morphology of fossil species (including characters from all the major anatomical regions of the body), whereas morphometric data is usually limited to anatomical regions with good degree of preservation across all available species, which also reduces the number of fossil taxa that can be included. Therefore, phylogenetic characters provide a suitable measure of morphological disparity, especially when assessing deep time macroevolutionary problems. Yet, important adaptations and considerations of phylogenetic datasets need to be taken into account for such kind of analyses, as further described below.

Large amounts of missing data, usually above 25%, considerably reduce the overall distance between taxa that can be captured on ordination spaces on both empirical and simulated datasets^60,87^. To reduce the negative impact of missing data, we removed all characters with more than 30% of missing data from the dataset, which resulted in a total of 19% missing data on the final dataset—safely below the threshold of 25%^60,87,88^. Additionally, inapplicable characters are a big conceptual problem to construct a morphospace. Taxa with inapplicable characters will have their placement enforced upon a space they do not reside in (which is conceptually very different from missing data—when they reside in that space, but we currently lack data to place them)^88^. Deleting all inapplicable characters would further decrease the number of utilized characters at about 30%, thus reducing the span of morphological representation in the dataset. Therefore, to avoid inapplicable characters, but keep minimal representation, we deleted all characters that were inapplicable to more than 5% of taxa, rescoring the remaining cells as missing data. Polymorphisms were converted into NA scores (treated as “?” during analyses), following previous recommendations and based on the reasoning above^85,88^.

Autapomorphies, if unevenly sampled across taxa, may also contribute to bias distance matrices. However, if autapomorphies are uniformly distributed across terminal taxa, then their overall effect is to increase overall pairwise distance between terminal taxa uniformly, therefore not creating biasing the interpretation of the data^89^. In the present dataset, there are some directly observed autapomorphies, which were already excluded during the removal of characters with large amounts of missing data (see above). Having no remaining autapomorphies in the dataset is another way of having a uniform distribution of autapomorphies, guaranteeing that no taxon will have additional dimensions separating it from other taxa in the dissimilarity matrix and morphospace ordination procedure.

### Intertaxon distance matrix and ordination matrix

The procedures above resulted in a final reduced matrix of 138 taxa and 105 characters. This number of characters is more than sufficient to provide reasonable estimates of disparity^90^. This dataset (dataset 1) was used to construct a morphospace for all sampled clades of reptiles. A second version of the dataset (dataset 2) was adapted to compare disparity across time. Since most post-Triassic taxa in the data matrix are lepidosaurs, we deleted the only three non-lepidosaurian post-Triassic taxa (*Kayentachelys*, *Philydrosaurus* and *Champsosaurus*), in order to distinguish disparity across time among early diapsids clades in general (between the Late Carboniferous and Late Triassic) and disparity across time among lepidosaurs (Early Jurassic to the present). Therefore, dataset 2 contained 135 taxa and 105 characters (and the original time-calibrated tree was also pruned of those three taxa to match the reduced dataset).

Using the reduced datasets and the time-calibrated trees, we constructed an intertaxon distance matrix **D** and an ordination matrix using principal coordinate analysis (PCoA— or classical multidimensional scaling). We implemented MORD as our method of estimating pairwise taxon distances for the distance matrix **D** and the subsequent ordination matrix, made available through the Claddis R package^60^, implementing Cailliez’s correction for negative eigenvalues. Additionally, we increased our sample size by including internal nodes using ancestral state reconstructions through the recently developed pre-OASE1 method^91^. This procedure provides a much better approximation of the true morphospace when compared to methods to reconstruct ancestral nodes in most previous disparity studies using ancestral state reconstructions^91^.

### Morphological disparity measures

Here, we used the sum of the variances (a post-ordination metric), which is comparatively robust to sample size and is not affected by the orientation of the coordinate axes of the ordination analysis^85,90^. Importantly, only post-ordination methods can be used to produce a morphospace projection. Further, post-ordination methods have been more widely used in the literature making our results more directly comparable to previous studies. Since PCo scores based on phylogenetic data usually have the first principle axis representing a small proportion of the total variance (usually the first two PCo representing less than 50% of total variance), morphospace representation using PCo scores should be taken with caution. This problem can be avoided in our assessment of disparity across time (our main measure of both chronological and taxonomic changes in disparity), in which it is possible to take into account all axes of variation to estimate morphological disparity. Nonmetric multidimensional scaling was not used to preserve the metric properties of the dissimilarity matrix. To measure morphological disparity across successive time bins, we used the R package dispRity^92^ to subdivide the data across time bins. Our data are not evenly sampled across time, as it was designed to maximize taxonomic representation across stratigraphic intervals. Therefore, uniform time bins would create more heterogenic sample sizes with some bins containing drastically low sample values, besides not capturing important geological boundaries reflective of important environmental shifts and mass extinctions. For those reasons, we chose time bins approximating stratigraphic intervals to subdivide our data chronologically, which enables capturing changes across major mass extinctions at stratigraphic boundaries (e.g. Permian–Triassic and Cretaceous–Palaeogene mass extinctions), and also less heterogenic sample sizes across the bins.

### Statistical analysis

We performed pairwise statistical tests across time bins to assess whether particular time intervals (approximating stratigraphic intervals, as used for disparity analysis above) have significantly different mean values for phenotypic and molecular evolutionary rates, as well as for morphological disparity. We assessed the normality of the distribution for each time bin using the Shapiro–Wilk normality test and visual assessment of data distribution. Normality of distribution of the residuals was assessed in the same manner. Additionally, we used the Fligner–Killeen test of homogeneity of variances to assess homoscedasticity in the data. For datasets not conforming to those expectations of parametric tests, we performed data transformations (square root, cube root, exponential, and log transformations). Subsequently to data transformations, Shapiro–Wilk and Fligner–Killeen tests were repeated. For datasets passing those tests, we performed pairwise t-tests among groups to detect significant differences in mean rates or disparity values between each pair of time bins. For datasets not passing those tests, we performed the equivalent nonparametric version: pairwise Wilcoxon rank sum (Mann–Whitney) tests. Additional methodological details can be found in Supplementary Methods. Summary statistics are available in Supplementary Tables 3–7, summarized results of statistical tests are available in Supplementary Tables 8–12, and detailed explanation and results of all statistical tests are available in Supplementary Data 5.

### Reporting summary

Further information on research design is available in the Nature Research Reporting Summary linked to this article.

## Supplementary information

Supplementary Information

Peer Review File

Reporting Summary

Description of Additional Supplementary Files

Supplementary Data 1 − 5

## Data Availability

All morphological and molecular data generated and analyzed, along with trees, log files, prior parameters and posterior parameter values described in the results and figures, and detailed results of statistical tests is available online as Supplementary Data files 1–5 at Harvard’s Dataverse Repository [10.7910/DVN/ZONWD0]^93^. All GenBank accession numbers are provided in Supplementary Table 1. Source data are provided with this paper.

## Source data

Source Data
