# Supplementary material for: Megaevolutionary dynamics and the timing of evolutionary innovation in reptiles
Source: Nat Commun. 2020 Jul 3;11:3322. doi: 10.1038/s41467-020-17190-9 (PMC7335191; doi:10.1038/s41467-020-17190-9)
Supplement: Supplementary file 1 — Supplementary Information [file 41467_2020_17190_MOESM1_ESM.pdf]

# SUPPLEMENTARY INFORMATION

Simões, T.R. et al.: **Megaevolutionary dynamics and the timing of evolutionary innovation in reptiles**

## TABLE OF CONTENTS:

**Supplementary Figures**

**Supplementary Methods**

**Supplementary Tables**

**Supplementary Table 1: GenBank accession numbers**

**Supplementary Table 2: 95% HPD range of divergence times for selected nodes under different tree prior parameters.**

**Supplementary References**

# Supplementary Figures

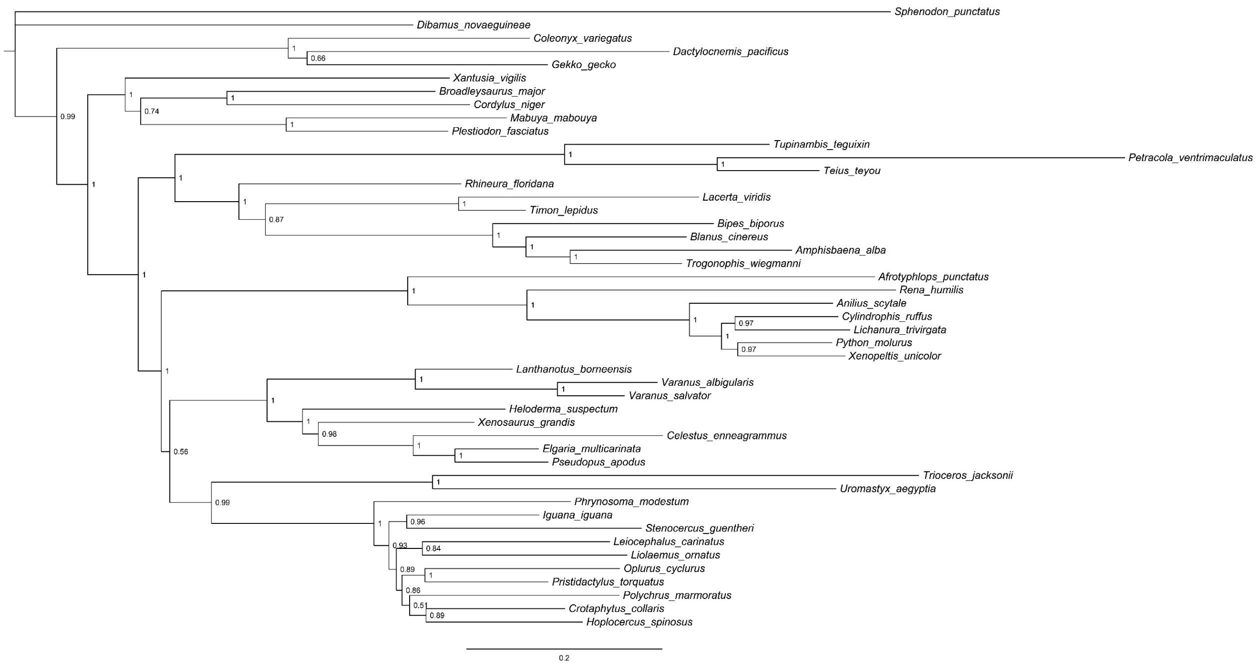


Supplementary Fig. 1. Majority rule consensus tree obtained from the non-clock Bayesian inference analyses of the molecular data only.

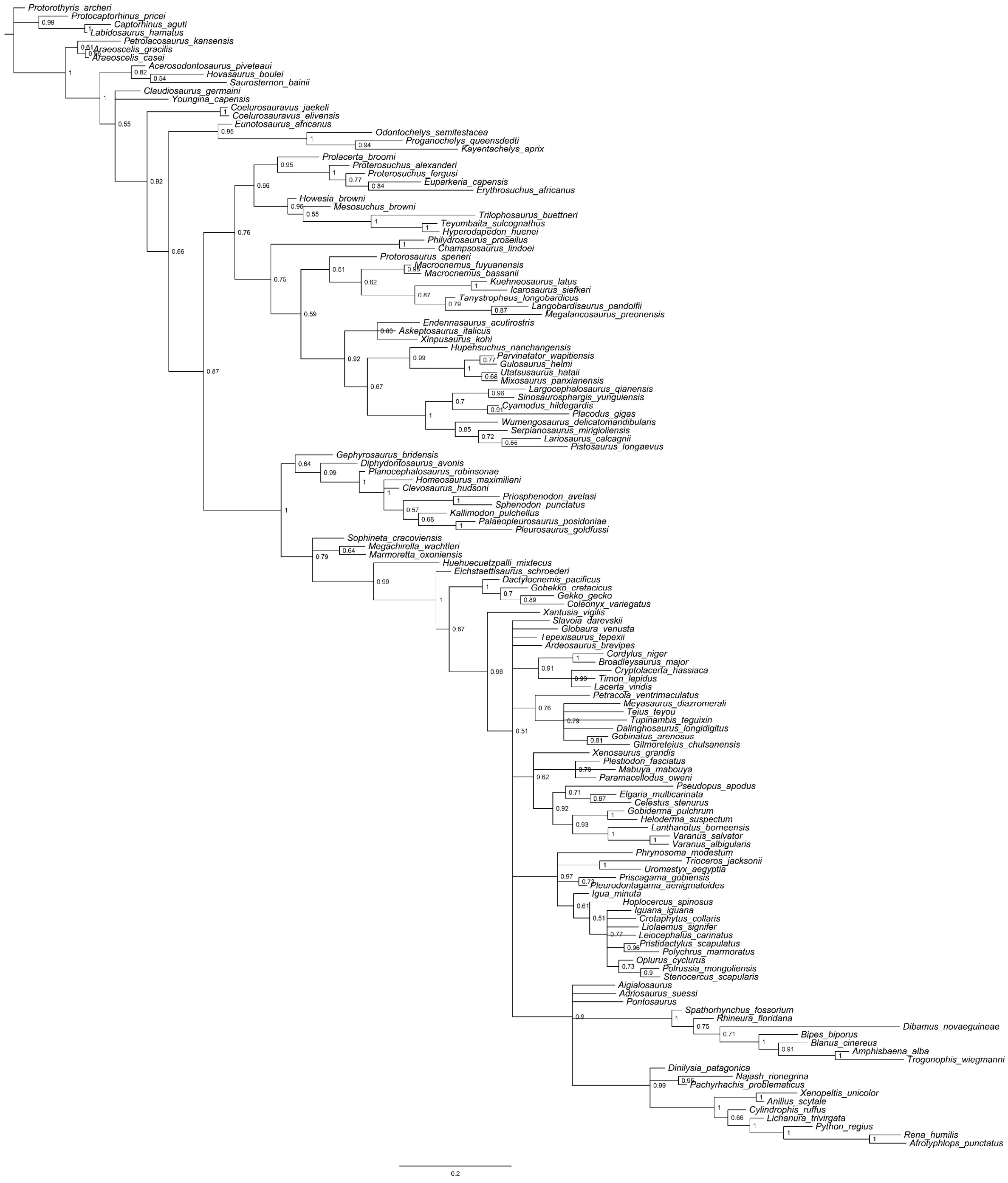

Supplementary Fig. 2. Majority rule consensus tree obtained from the non-clock Bayesian inference analyses of the morphological data only.

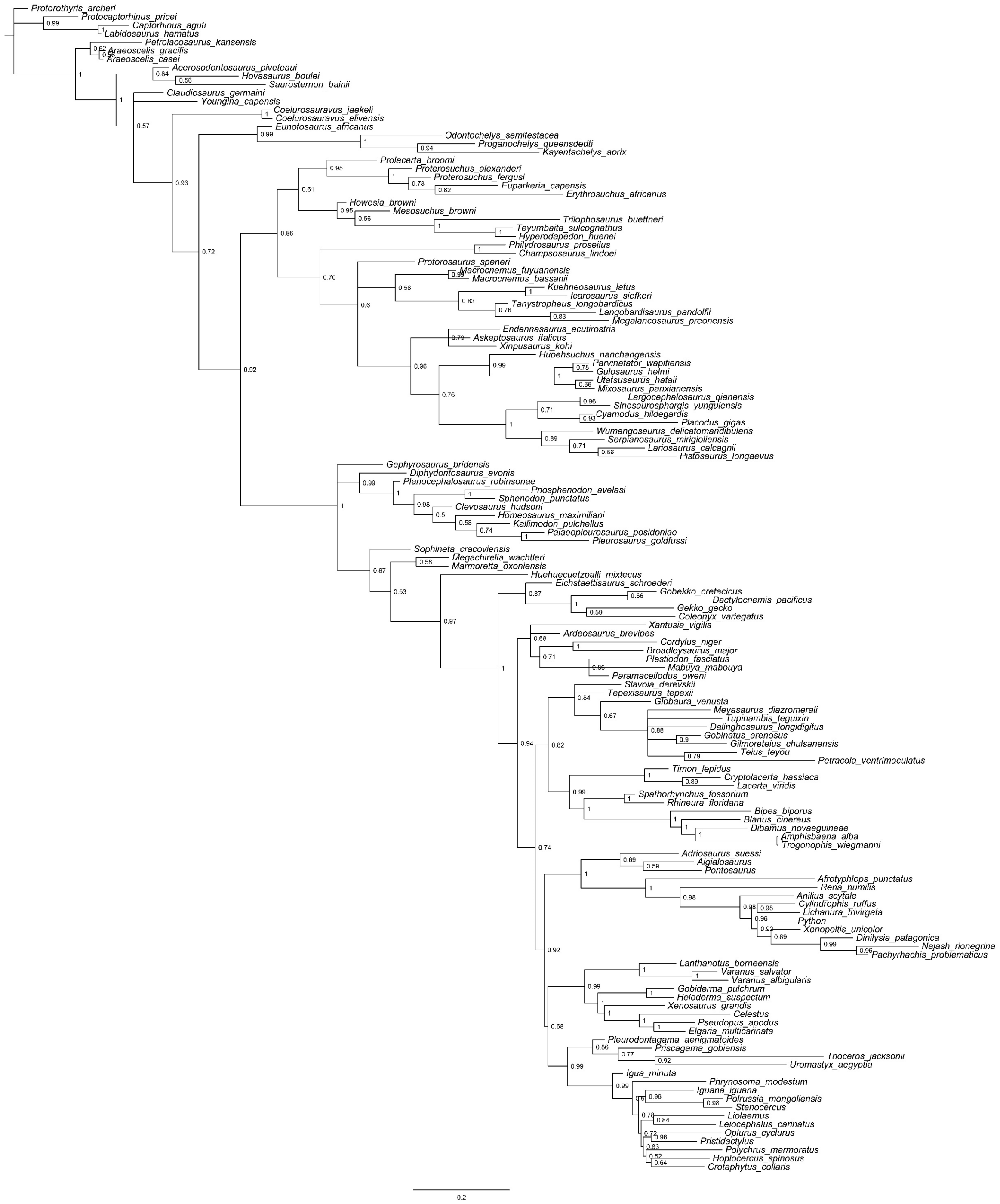

Supplementary Fig. 3. Maximum compatible tree obtained from the non-clock Bayesian inference analyses of the combined evidence data.

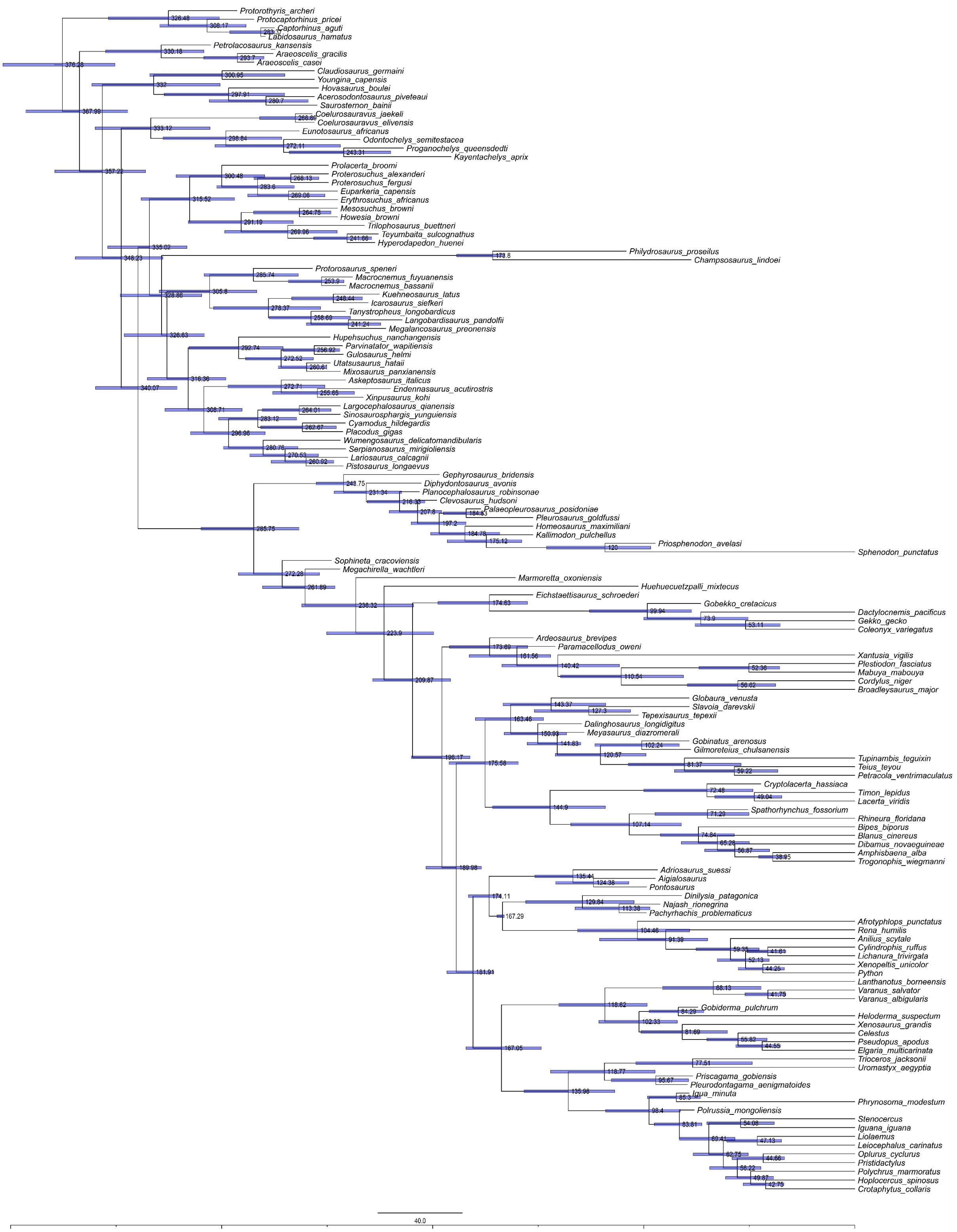


Supplementary Fig. 4. Maximum compatible tree from Mr. Bayes (total-evidence dating relaxed clock Bayesian inference) without DRA correction. Node values represent median divergence times and node bars the 95%HPD of age distribution.

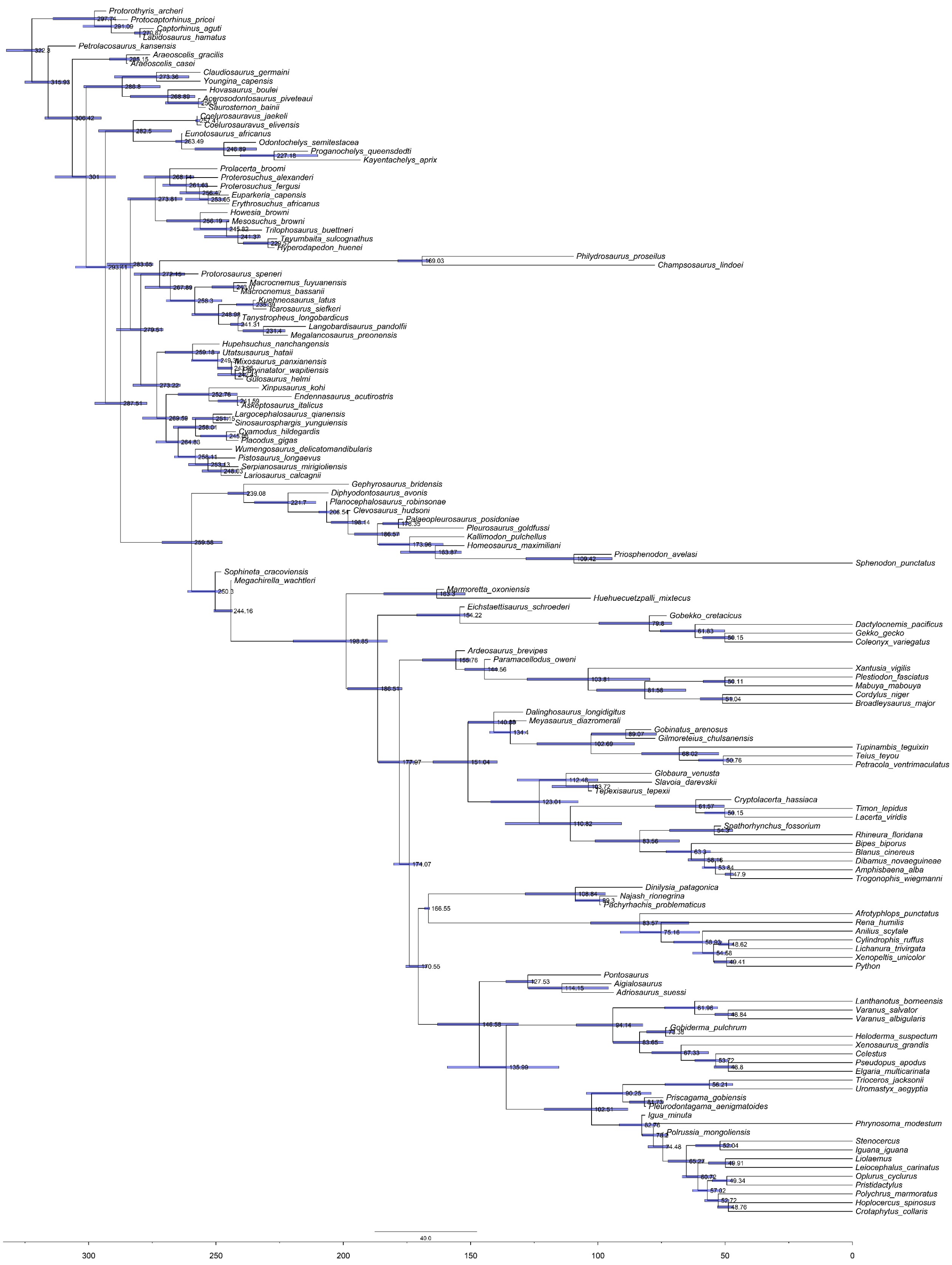


Supplementary Fig. 5. Maximum compatible tree from Mr. Bayes (total-evidence dating relaxed clock Bayesian inference) with DRA correction. Node values represent median divergence times and node bars the 95%HPD of age distribution.

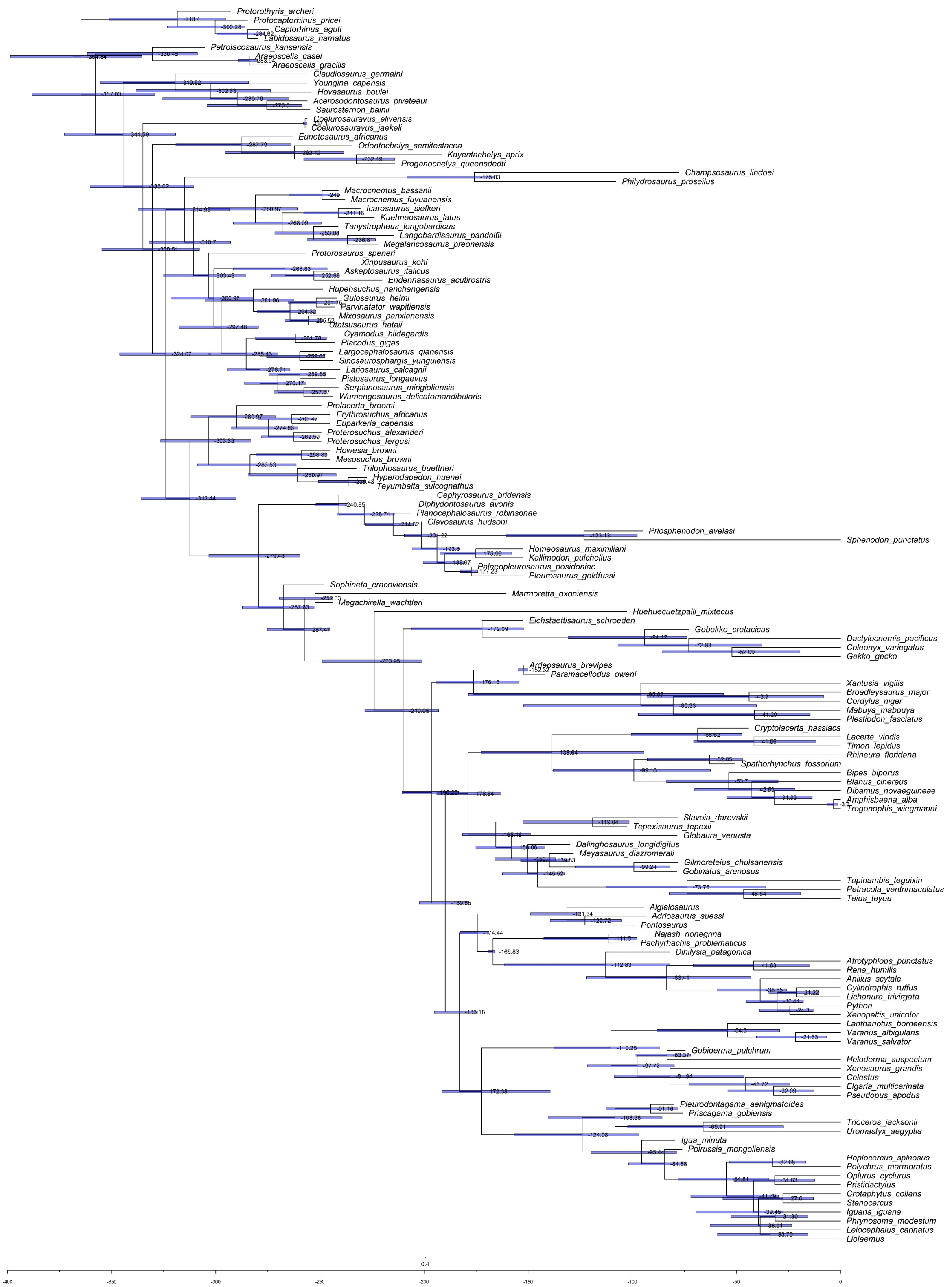


Supplementary Fig. 6. Maximum clade credibility tree from BEAST2 (total-evidence dating relaxed clock Bayesian inference) without DRA correction. Node values represent median divergence times and node bars the 95%HPD of age distribution.

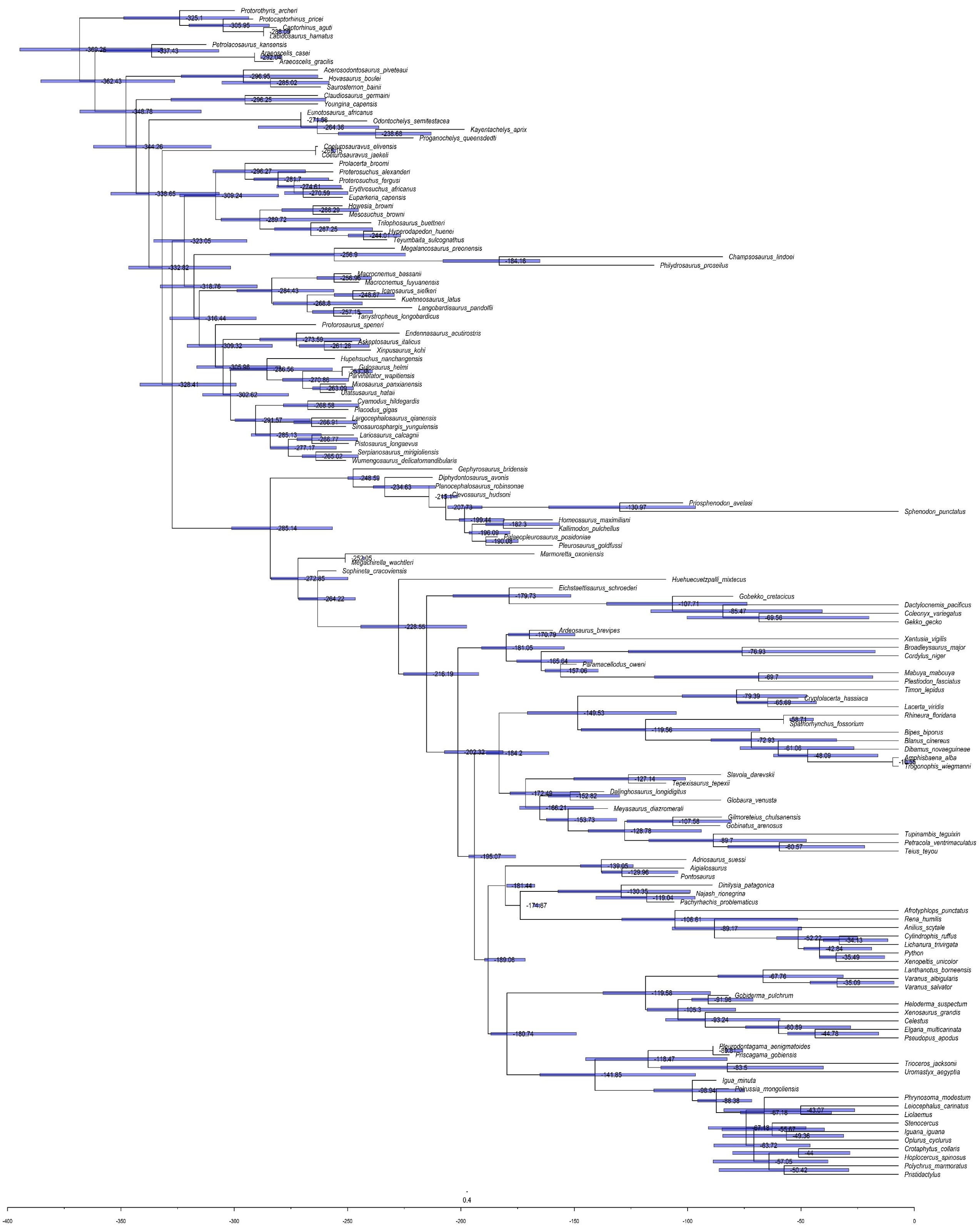

Supplementary Fig. 7. Maximum clade credibility tree from BEAST2 (total-evidence dating relaxed clock Bayesian inference) with DRA correction. Node values represent median divergence times and node bars the 95%HPD of age distribution.

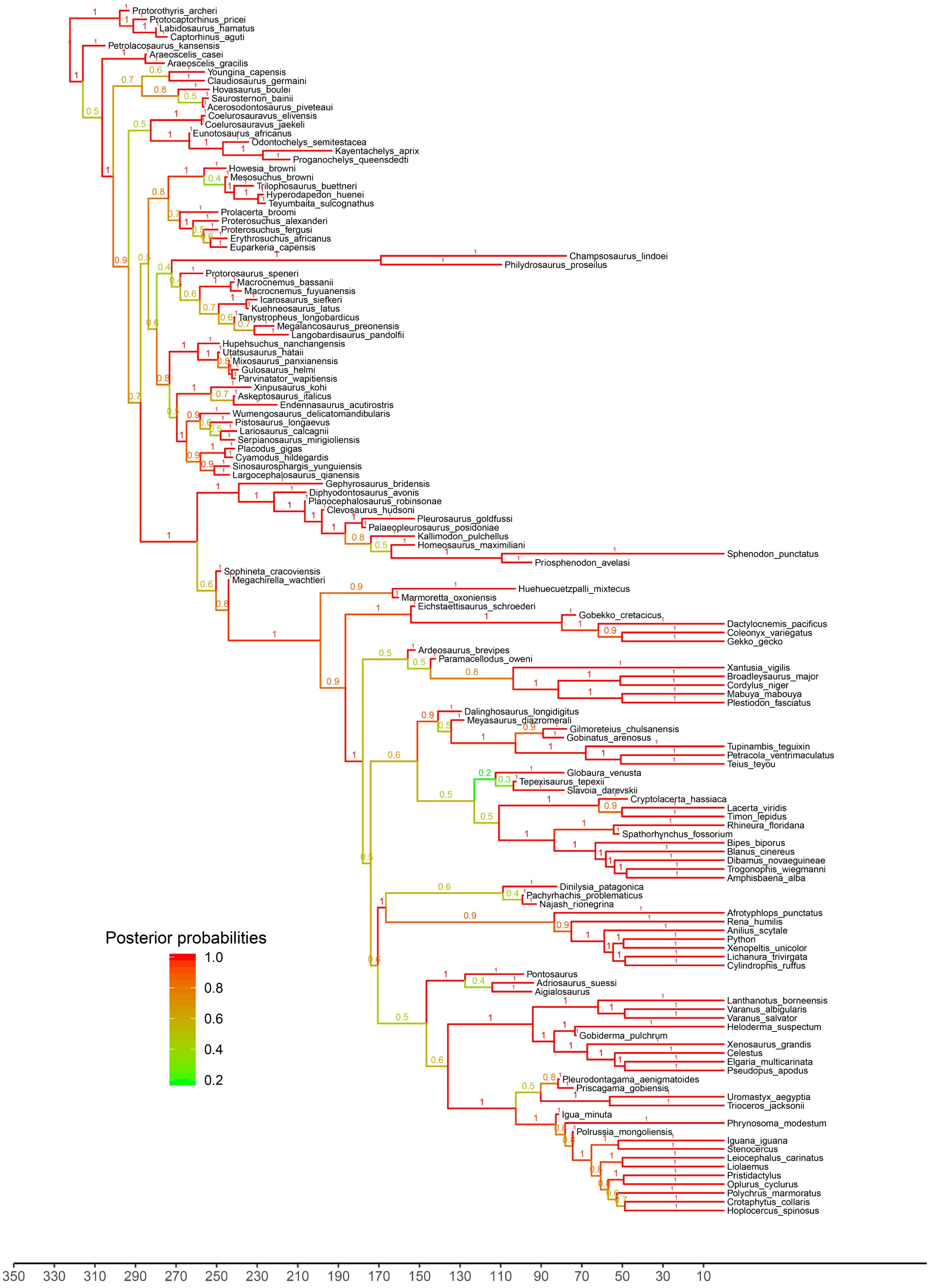


Supplementary Fig. 8. Maximum compatible tree from Mr. Bayes (total-evidence dating relaxed clock Bayesian inference) with DRA correction. Node values and branch colours represent clade posterior probabilities.

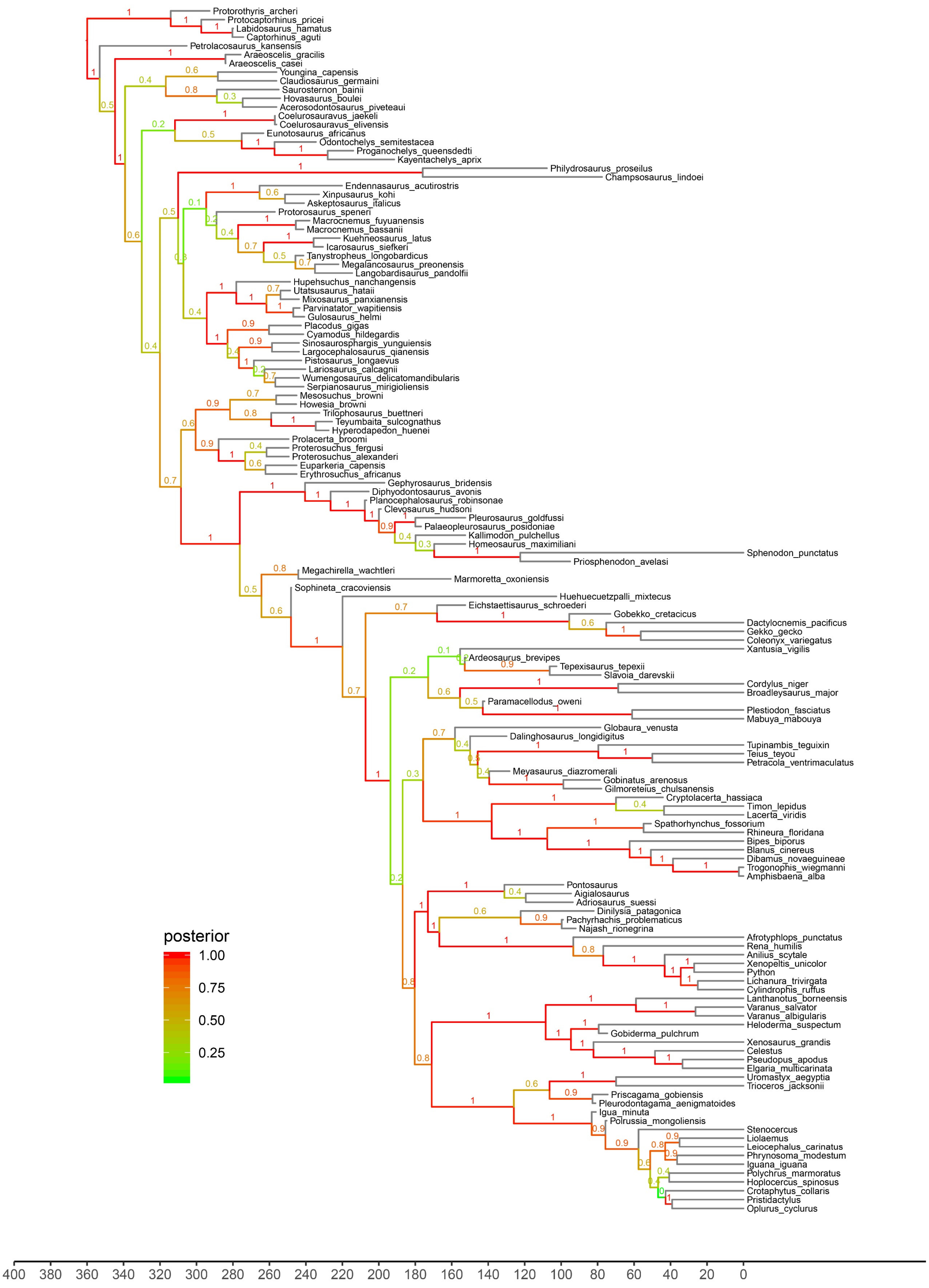


Supplementary Fig. 9. Maximum clade credibility tree from BEAST2 (total-evidence dating relaxed clock Bayesian inference) with DRA correction. Node values and branch colours represent clade posterior probabilities.

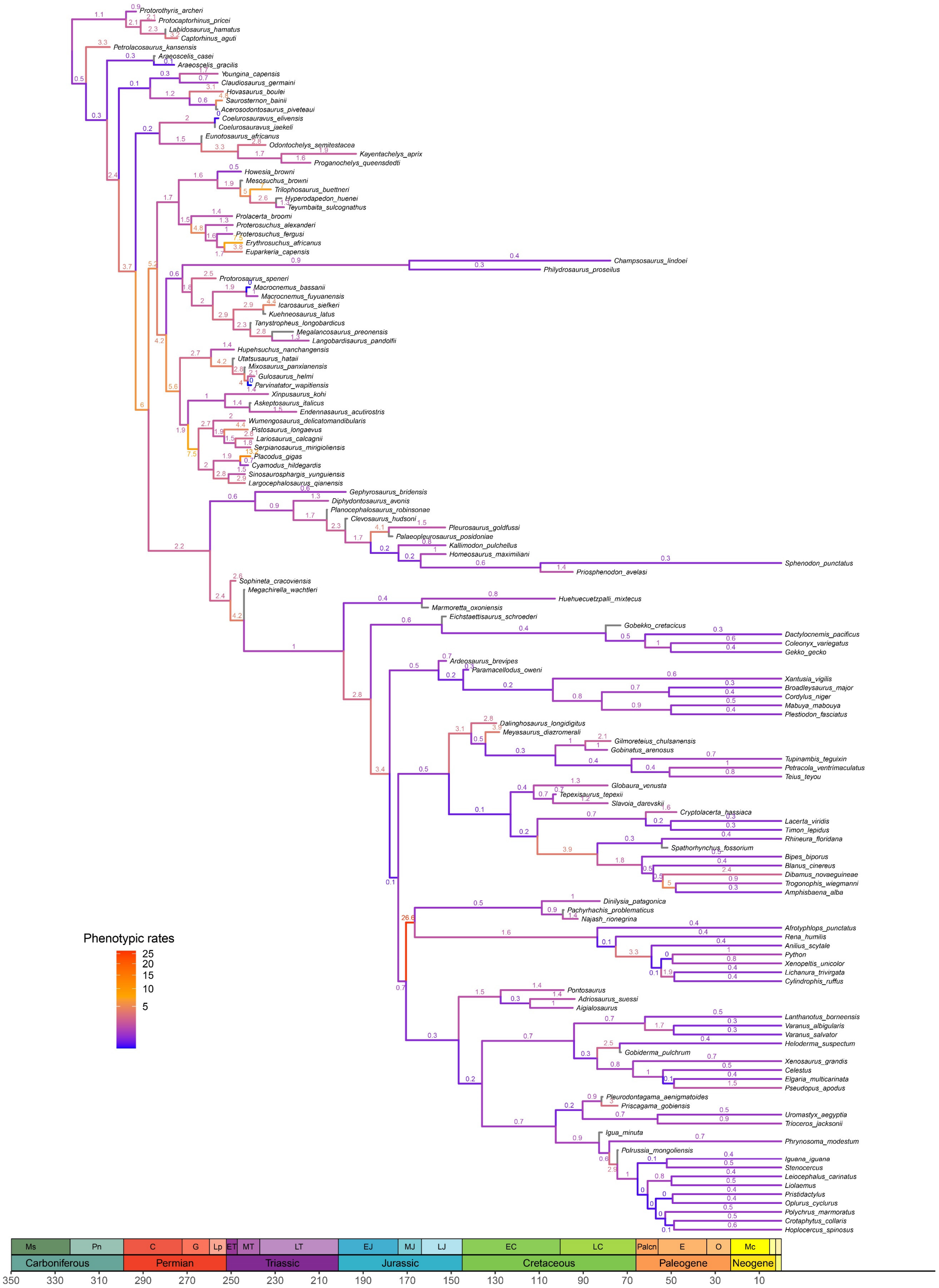


Supplementary Fig. 10. Maximum compatible tree from Mr. Bayes (total-evidence dating relaxed clock Bayesian inference) with DRA correction. Node values and branch colours represent rates of morphological evolution in early diapsids and lepidosaurs. Gray fossil tips indicate tips with zero/extremely short branch lengths (e.g., tips inferred to represent ancestors), for which rate values could not be meaningfully estimated by Mr. Bayes.

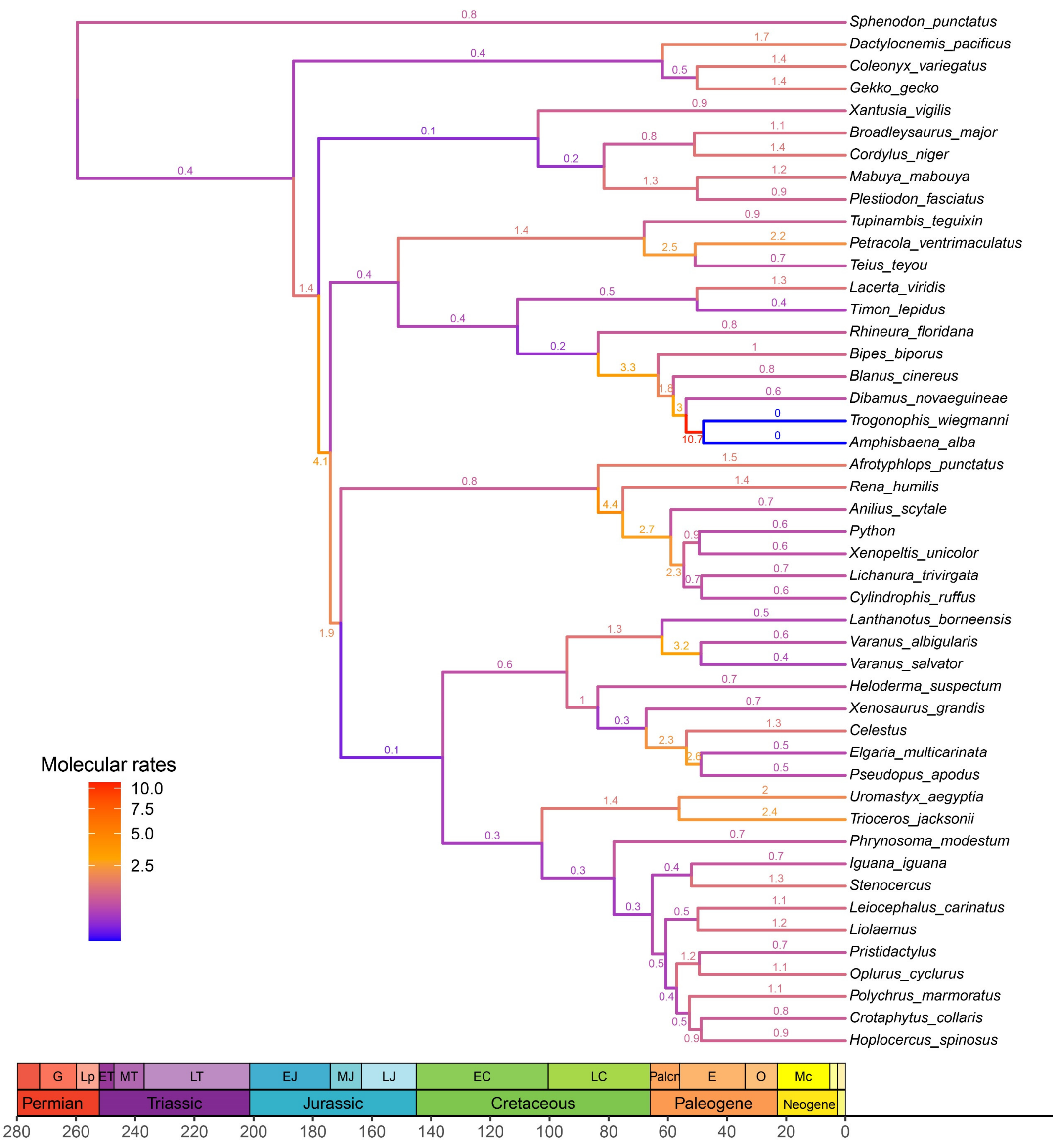


Supplementary Fig. 11. Maximum compatible tree from Mr. Bayes (total-evidence dating relaxed clock Bayesian inference) with DRA correction. Node values and branch colours represent rates of molecular evolution in extant lepidosaurs.

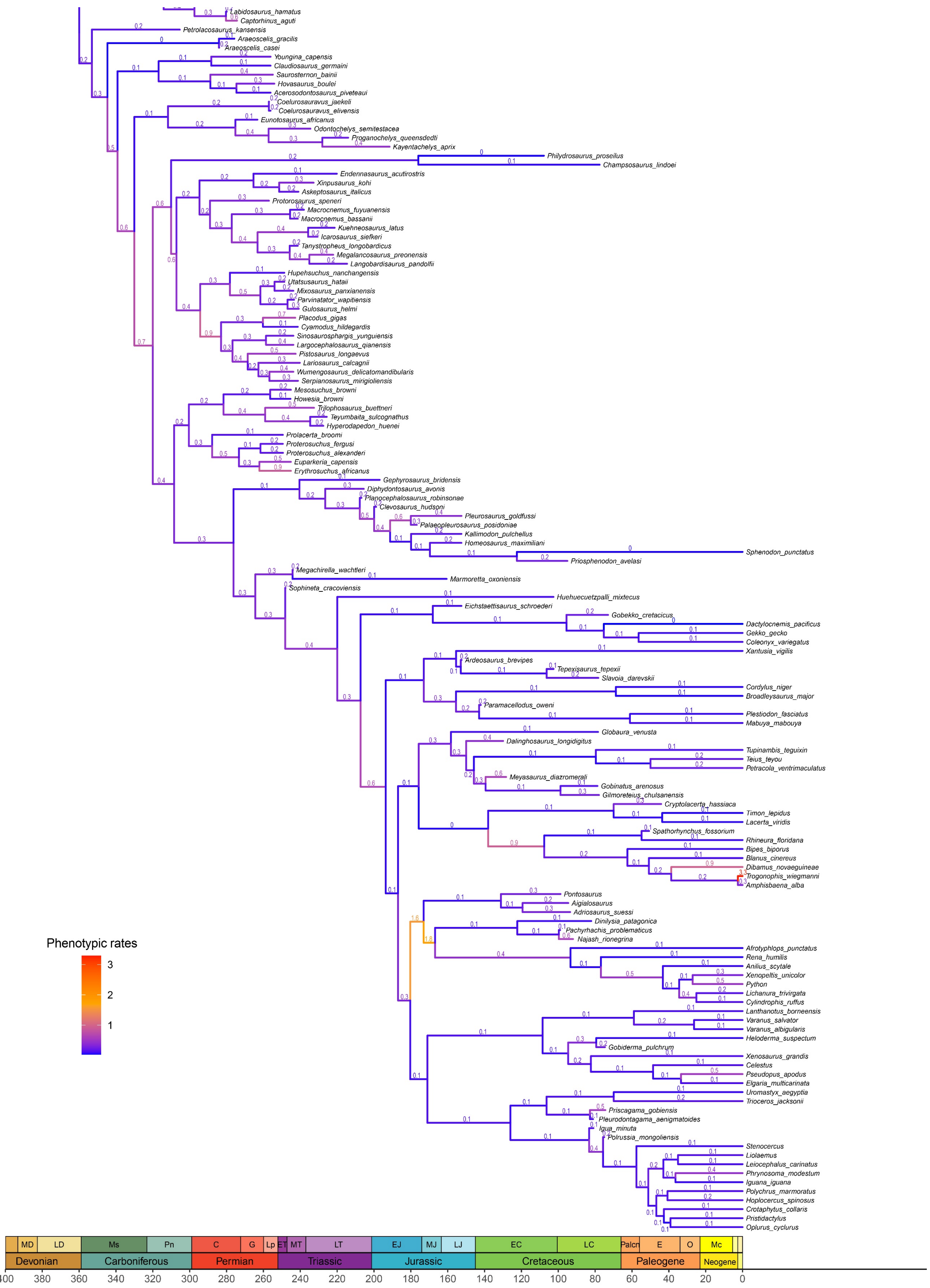


Supplementary Fig. 12. Maximum clade credibility tree from BEAST2 (total-evidence dating relaxed clock Bayesian inference) with DRA correction. Node values and branch colours represent rates of morphological evolution in early diapsids and lepidosaurs.

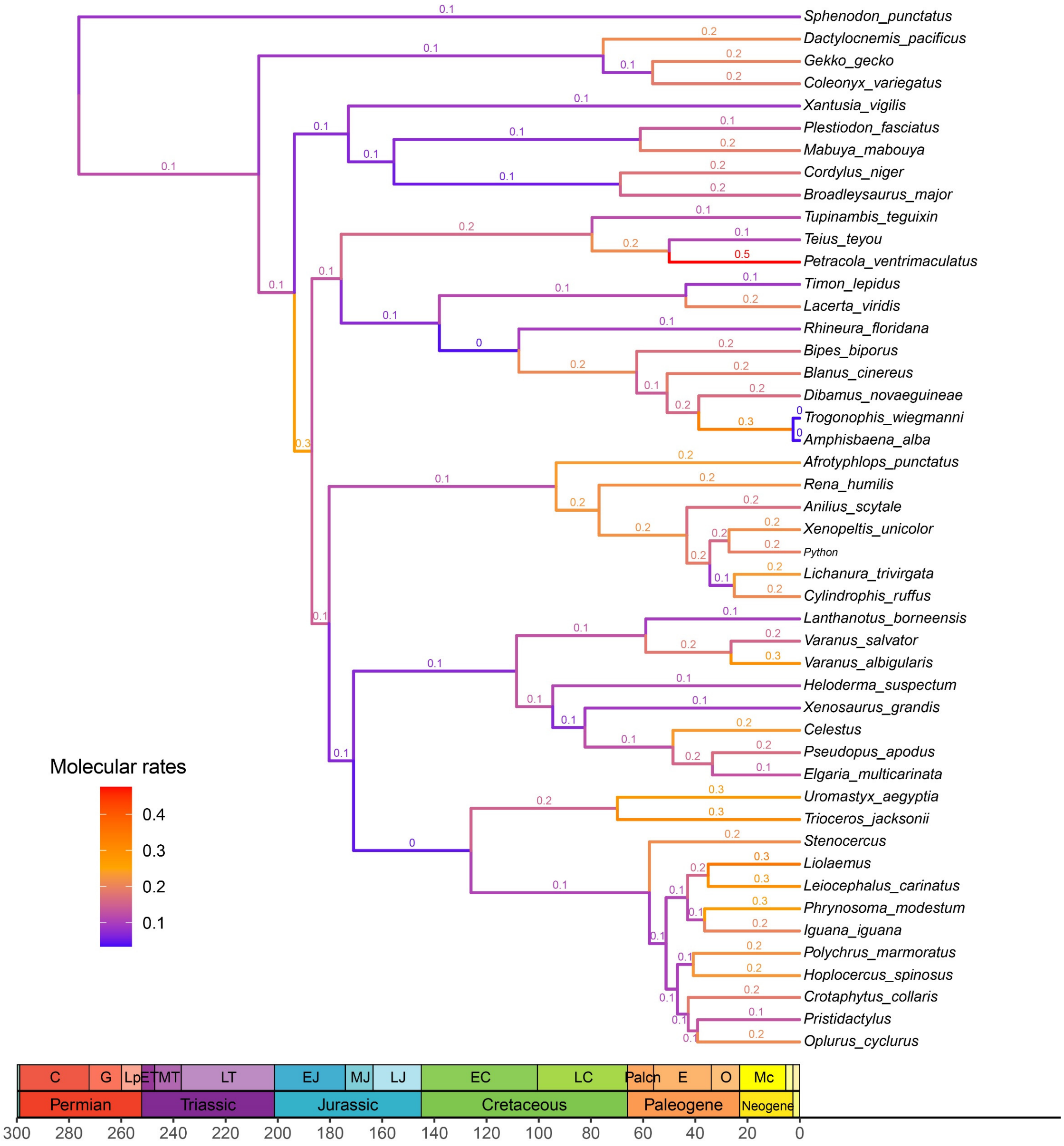


Supplementary Fig. 13. Maximum clade credibility tree from BEAST2 (total-evidence dating relaxed clock Bayesian inference) with DRA correction. Node values and branch colours represent rates of molecular evolution in extant lepidosaurs.

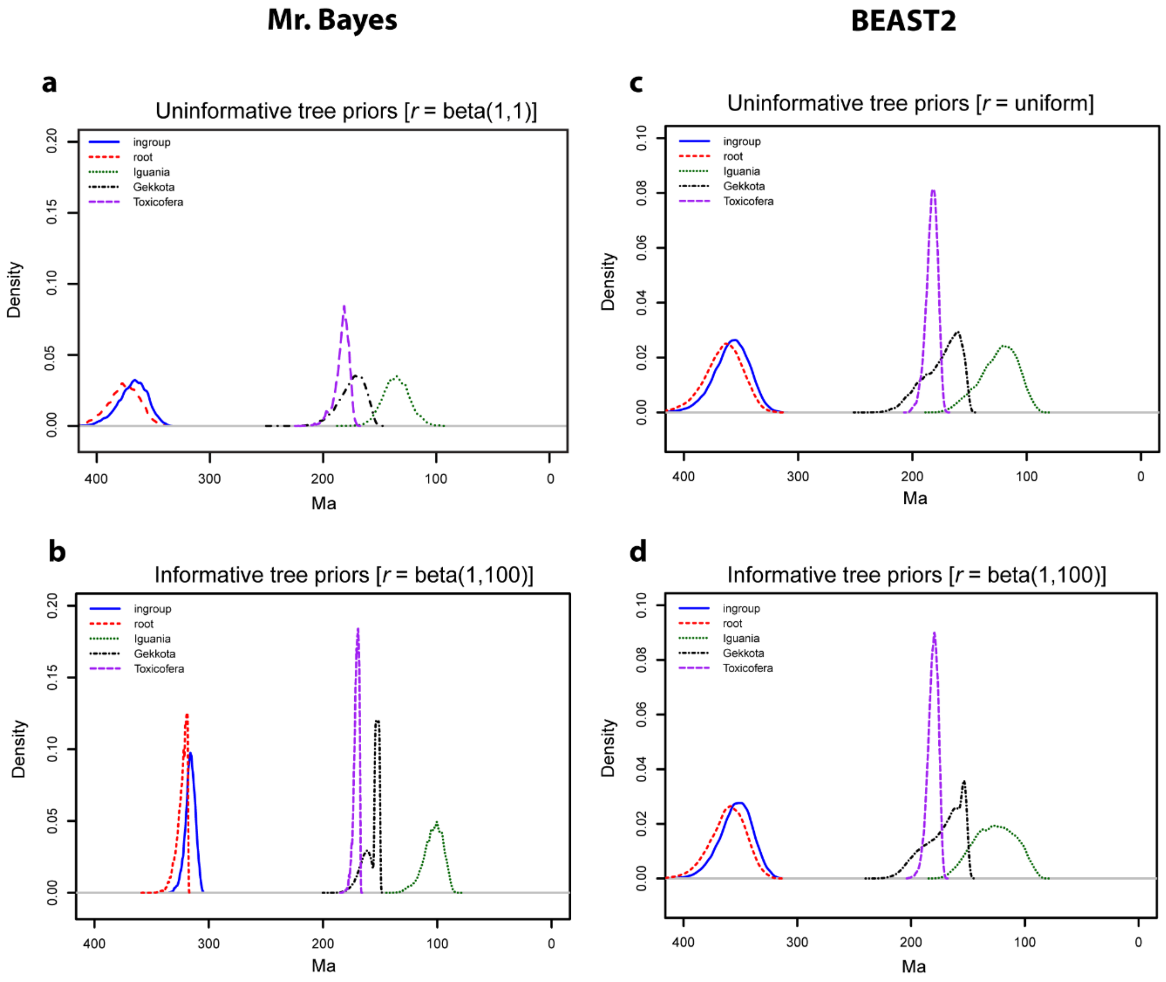


Supplementary Fig. 14. Posterior age distribution for selected clades before and after correction for deep root attraction in the analyses performed in both MrBayes and BEAST2.

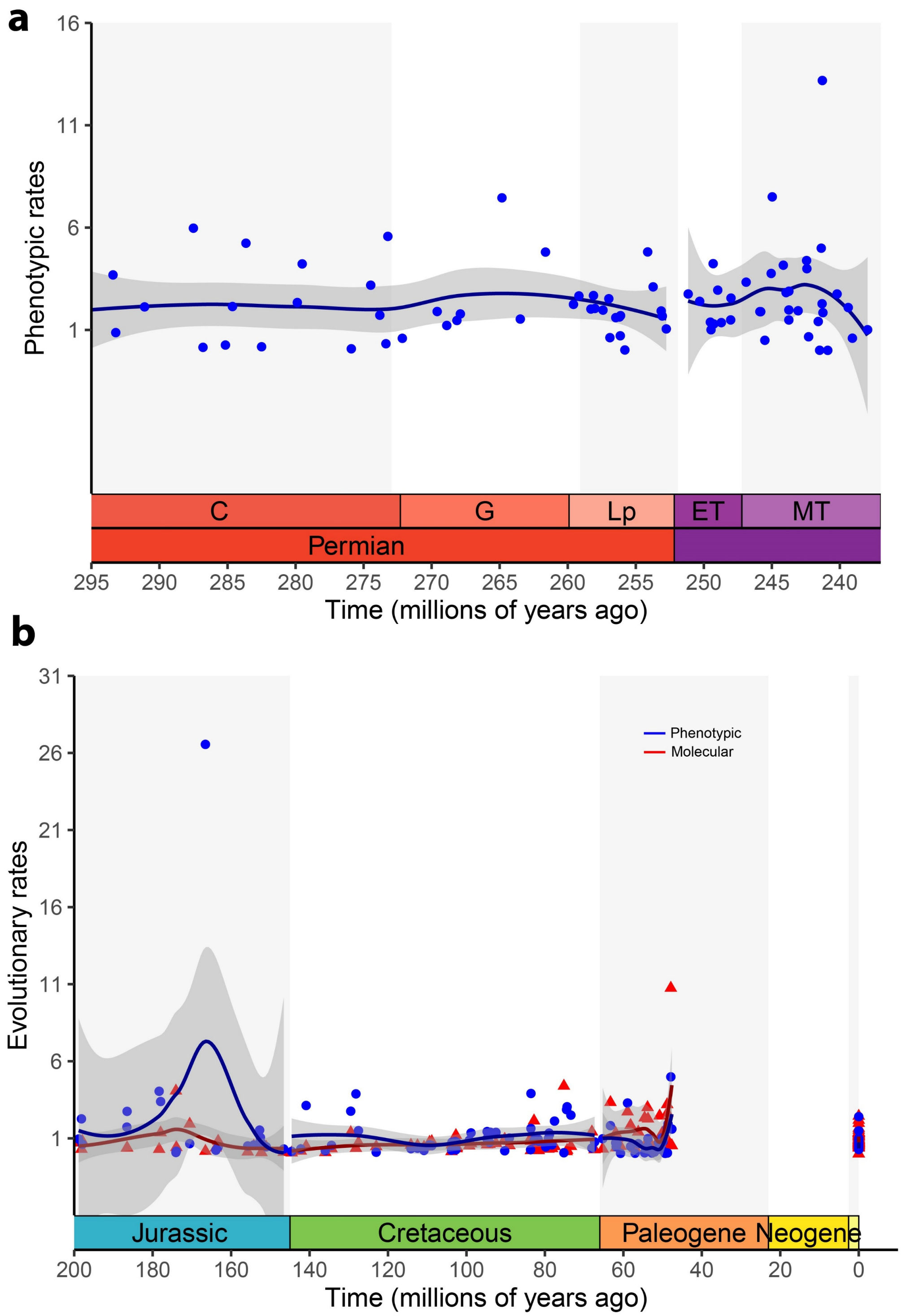


Supplementary Fig. 15. **a**, phenotypic evolutionary rates in early diapsids (n=79 phenotypic evolutionary rates pooled from the maximum compatible tree from Mr. Bayes). Grey area represents 95% CI around LOESS regression line segmented by geological time bins. **b**, phenotypic (blue) and molecular (red) evolutionary rates in lepidosaurs (n=146 for molecular and phenotypic evolutionary rates pooled from the maximum compatible tree from Mr. Bayes). Grey area represents 95% CI around LOESS regression line segmented by geological time bins (phenotypic = dark blue and molecular = dark red).

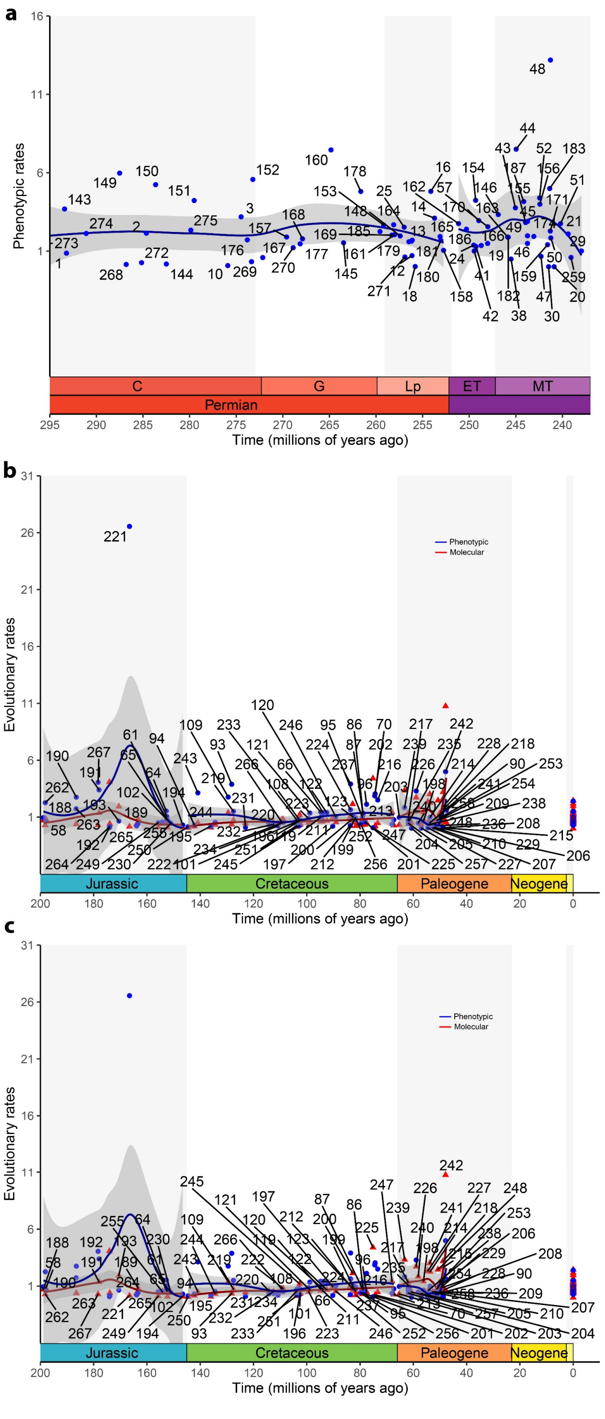


Supplementary Fig. 16. Node numbers from the maximum compatible tree from MrBayes for the plots in Suppl. Fig. 15. **a**, phenotypic evolutionary rates in early diapsids (n=79 phenotypic evolutionary rates pooled from the maximum compatible tree from Mr. Bayes). Grey area represents 95% CI around LOESS regression line segmented by geological time bins. **b** and **c**, phenotypic (blue) and molecular (red) evolutionary rates in lepidosaurs (n=146 for molecular and phenotypic evolutionary rates pooled from the maximum compatible tree from Mr. Bayes). ). Grey area represents 95% CI around LOESS regression line segmented by geological time bins (phenotypic = dark blue and molecular = dark red).

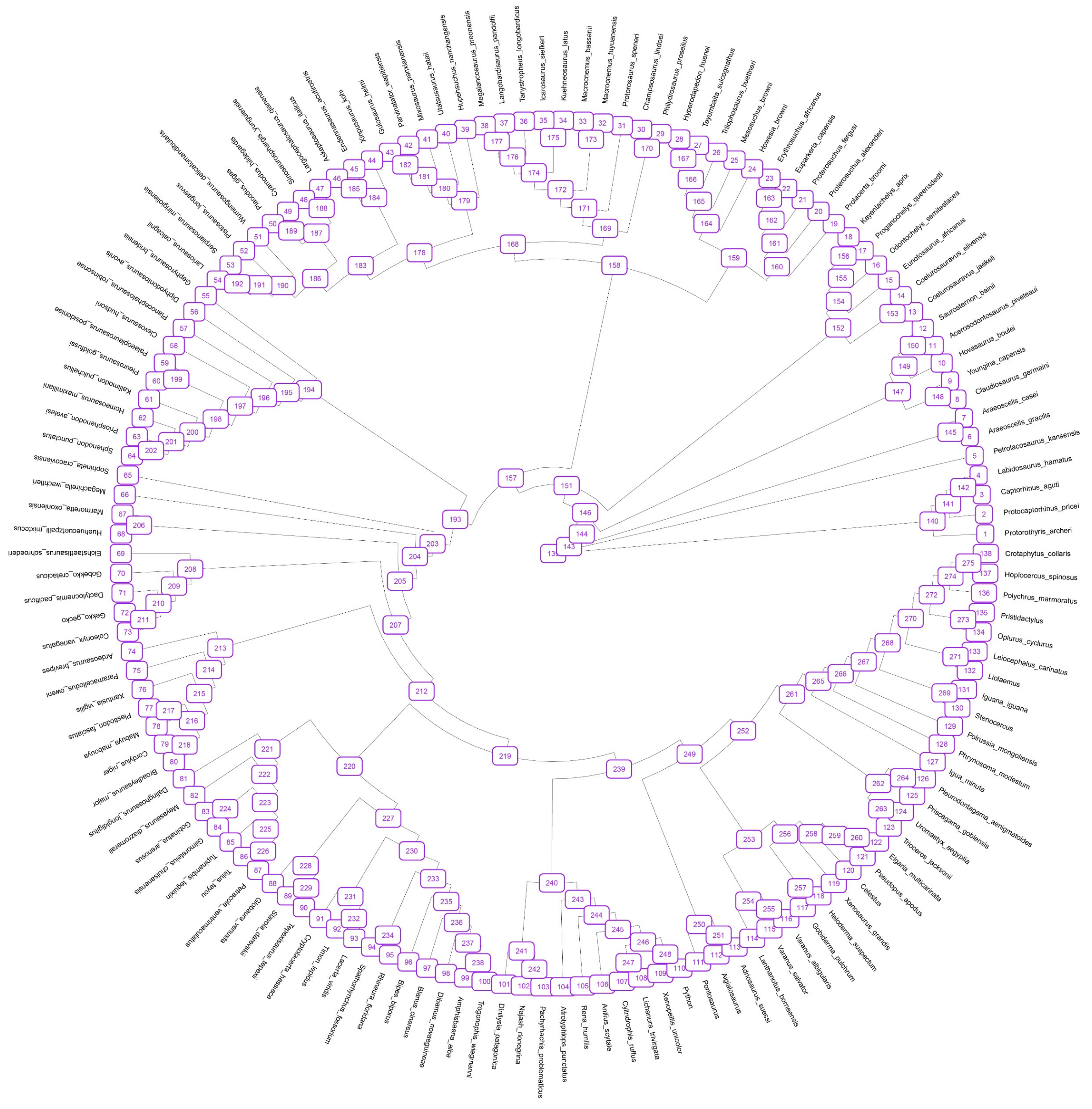

Supplementary Fig. 17. Node numbers for the maximum compatible tree from MrBayes.

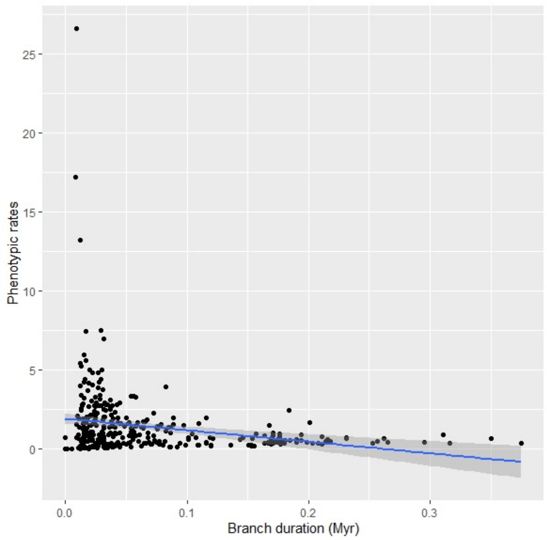


Supplementary Fig. 18. Linear regression between phenotypic rates and relative branch durations for all data points—early diapsids and lepidosaurs (n=347 unique bipartitions from the posterior trees; R-squared: 0.0543; p-value: 1.157e-05; two-sided regression with no data transformation). Shaded gray area = 95% CI. There is a significant (although weak) tendency for short branches to experience high phenotypic rates in the present data set, and longer branches to exhibit slower rate values. This corresponds to the usual expectation of exponentially distributed rates of phenotypic character change used for modeling morphological characters [1,2].

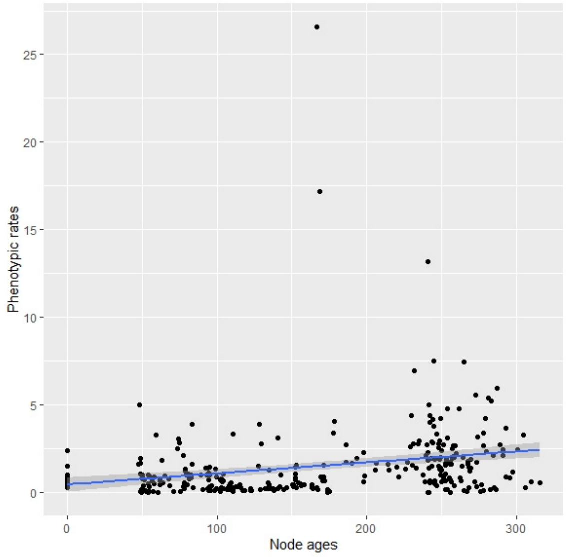


Supplementary Fig. 19. Linear regression between phenotypic rates and node ages for all data points—early diapsids and lepidosaurs (n=347 unique bipartitions from the posterior trees; R-squared: 0.07823; p-value: 1.174e-07; two-sided regression with no data transformation). Shaded gray area = 95% CI. There is a significant (although weak) tendency for older nodes (at the origin and early evolution of the major diapsid clades) to have higher phenotypic evolutionary rates compared to younger nodes (pooled from lepidosaur data), as also detected from data in Fig.1a,b.

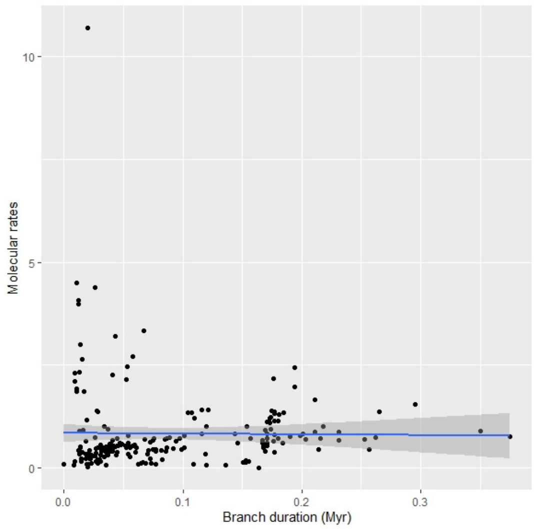


Supplementary Fig. 20. Linear regression between molecular rates and relative branch durations (extant tips and internal nodes) (n=211 unique bipartitions from the posterior trees; R-squared: 0.0001569 ; p-value: 0.8548; two-sided regression with no data transformation). Shaded gray area = 95% CI. No detectable correlation between duration of each branch and inferred molecular rates among lepidosaurs.

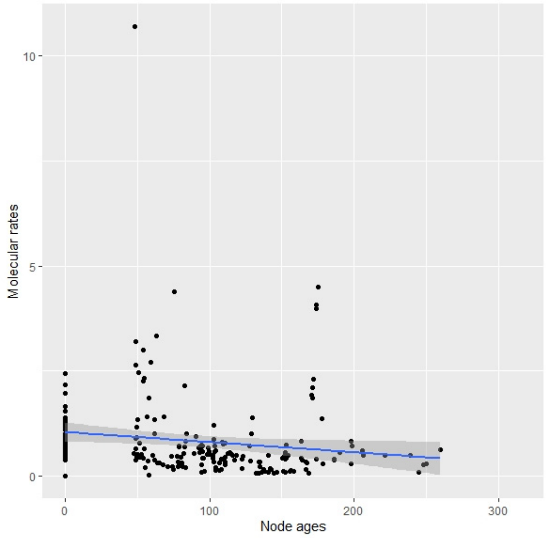


Supplementary Fig. 21. Linear regression between molecular rates and node ages for extant taxa (extant tips and internal nodes) (n=211 unique bipartitions from the posterior trees; R-squared: 0.01794; p-value: p-value: 0.02747; two-sided regression with no data transformation). Shaded gray area = 95% CI. There is a significant (although weak) tendency for older nodes (at the origin and early evolution of lepidosaurs) to have lower molecular evolutionary rates compared to younger nodes (as also detected from data in Fig.3a). This supports the decoupling of morphological and molecular rates at comparable nodes in lepidosaurs, as indicated in Fig 4.

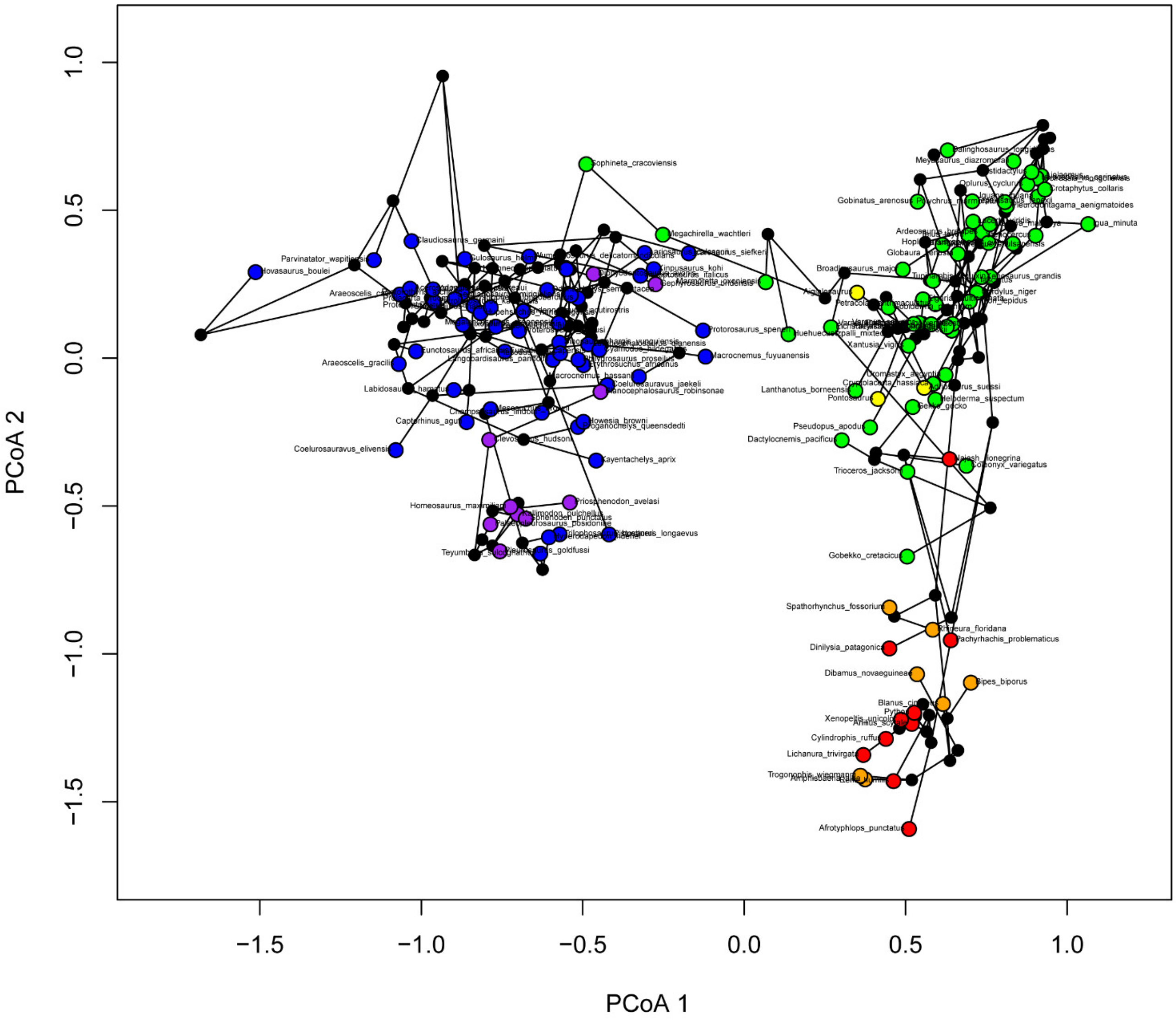


Supplementary Fig. 22. Phylomorphospace depicting all sampled species, showing individual taxon names.

# Supplementary Methods

The analyses were conducted following the same procedures as outlined in the original publication of this data set (Simões *et al.*[46]). Details on taxonomic sampling, observed specimens, morphological character construction, character list, age calibrations, analytical procedures, among others, can be found on the Methods section and, in substantial detail, in the Supplementary Information file available for Simões *et al.*[46]. Below, we detail modifications or additions to those procedures that were performed for the analyses conducted for the present study. Tree files, including all node and parameter values can be found online at: https://dataverse.harvard.edu/privateurl.xhtml?token=9f850dff-0ab7-44fb-a183-7b0d01d04ae3.

**Additional sampled taxa**

**Fossil taxa**

Stratigraphy and age for the newly included fossil taxa used for tip-dating calibrations. Calibration ages for the remaining fossil taxa can be found in the Supplementary Information file (Supplementary Table 2) of Simões *et al.*[46]

***Pleurosaurus goldfussi*** Meyer, 1831

**Age.** Latest Kimmeridgian-Tithonian, Late Jurassic [3-5].

**Horizon/Locality.** Solnhofen Plattenkalk—Solnhofen, Eichstätt, Monheim, Sappenfeld, Dailing and Wintershof, Bavaria, Germany; Calcaires Blancs (upper Kimmeridgian or lower Tithonian)—Quarry situated near Aiguines village (lower Tithonian), Canjuers, Var, France; Calcaire Lithographique—Cerin, Ain, France. [3,4,6].

**Holotype.** BSPG 1925 I 18 (observed)

**Observed referred materials.** BSPG 1978 I 7, MNHN 1983-4-CNJ 80.

**Main bibliography.** Meyer[7]; Münster[8]; Fitzinger[9]; Wagner[10]; Zittel[11]; Watson[12]; Broili[13]; Huene[14]; Hoffstetter[15]; Cocude-Michel[3]; Cocude-Michel[16]; Kuhn[17]; Carroll[18]; Carroll[19]; Carroll and Wild[20]; Dupret[4]; Jones[21]; Evans and Jones[22].

**Remarks**. Specimen BSPG 1978 I 7 labeled as *P. ginsburgi* is assigned here to *P. goldfussi* based on the skull morphology. The specimen has a fused and relatively elongate postorbitofrontal, followed by an anteroposteriorly short squamosal. This condition is also seen in *P. goldfussi* (BSPG 1925 I 18) and differs from *P. ginsburgi* (BSPG 1977 XIX 40), which has separate postorbital and posfrontals, as well as a comparatively longer squamosal.

***Cryptolacerta hassiaca*** Müller et al., 2011

**Age.** Lutetian, Eocene, Paleogene [23].

**Horizon/Locality.** West of Quarry 2, 50 cm above Level beta—Messel Pit World Heritage Site, Hesse, Germany [23].

**Holotype.** SMF ME 2604 (observed).

**Main bibliography.** Müller *et al.*[23]; Tałanda[24].

**Remarks.** Most of our interpretation agrees with the description provided by Müller *et al.*[23]. However, Müller *et al.*[23] stated that a lacrimal is absent in *Cryptolacerta*. However, in both left and right sides the region where the lacrimal would be located (between the prefrontal, maxilla

and jugal) is badly damaged. On the left side of the skull, a gap between the prefrontal and the jugal indicates a lacrimal could, in fact, have been present. Therefore, we re-interpret the lacrimal condition as a missing data for this specimen. Additionally, the limited preservation of the pectoral girdle elements [even in ventral view, as illustrated by the CT scan data by Müller *et al.*[23]] indicates it is hard to assess whether or not the interclavicle was present.

***Gobiderma pulchrum*** Borsuk-Bialynicka, 1984
**Age.** Campanian-earliest Maastrichtian, Late Cretaceous [25,26].
**Horizon/Locality.** Barun Goyot (Khermeen Tsav and Khulsan) and Djadochta Formation (Udan Sayr, Ukhaa Tolgod, Bayn Dzak and Tugrugeen Shireh), Nemegt Basin—Gobi Desert, Mongolia; Djadokhta Formation, Nemegt Basin—Bayan Mandahu redbeds, Inner Mongolia, China [27,28].
**Holotype.** ZPAL MgR-III/64 (observed)
**Observed referred materials.** ZPAL MgR-III/65, ZPAL MgR-III/66, ZPAL MgR-I/63.
**Main bibliography.** Borsuk-Białynicka[29]; Gao and Norell[27]; Conrad *et al.*[28].

**Extant taxa**
*Tupinambis teguixin*: FMNH 140193, CM 112011, CM 121599; *Celestus stenurus*: AMNH R-154690; *Varanus albigularis*: AMNH R-47726, UAMZ 947; *Amphisbaena alba*: UAMZ 399, *Trogonophis wiegmanni*: FMNH 2089, FMNH 265674, FMNH 266484; *Lichanura trivirgatta*: UAMZ 3819, *Python regius*: UAMZ 3818, *Rena humilis*: NMNH 222795, *Afrotyphlops punctatus*: NMNH 320704.

# Supplementary Tables

**Supplementary Table 1.** Loci accession numbers. Additional taxa sampled herein relative to Simões *et al.*[46] are highlighted in red.

| Taxon | BDNF | CAND1 | C-*mos* | CXCR4 |
|---|---|---|---|---|
| *Afrotyphlops punctatus* | GU902395.1 | | | |
| *Amphisbaena alba* | FJ441888.1 | | FJ441763.1 | |
| *Anilius scytale* | EU402625.1 | GU432630.1 | AF544722.1 | JN702453.1 |
| *Bipes biporus* | JN654794.1 | JN881176.1 | AF039482.1 | JN702381.1 |
| *Blanus cinereus* | | | AY444019.1 | |
| *Broadleysaurus major* | HM160588.1 | | EU366459.1 | |
| *Celestus enneagrammus* | GU457853.1 | GU432601.1 | | JN702361.1 |
| *Coleonyx variegatus* | HQ876231.1 | JF818522.1 | EU116676.1 | JN702309.1 |
| *Cordylus niger* | KT941174.1 | | KT941211.1 | |
| *Crotaphytus collaris* | JF806021.1 | JF818552.1 | AY987985.1 | JN702405.1 |
| *Cylindrophis ruffus* | EU402635.1 | JF818530.1 | AF471133.1 | JN702366.1 |
| *Dactylocnemis pacificus* | | | | |
| *Dibamus novaeguineae* | GU457863.1 | GU432611.1 | EF450999.1 | JN702424.1 |
| *Elgaria multicarinata* | GU457854.1 | GU432602.1 | AF039479.1 | JN702462.1 |
| *Gekko gecko* | EU402614.1 | GU432614.1 | EU366455.1 | JN702441.1 |
| *Heloderma suspectum* | GU457856.1 | GU432604.1 | AY487348.1 | |
| *Hoplocercus spinosus* | | | | |
| *Iguana iguana* | KR350713.1 | | AF148708.1 | |
| *Lacerta viridis* | GU457875.1 | GU432624.1 | DQ097132.1 | JN702397.1 |
| *Lanthanotus borneensis* | GU457859.1 | GU432607.1 | AY662564.1 | JN702444.1 |
| *Leiocephalus carinatus* | AY987970.1 | | | |
| *Lichanura trivirgata* | EU402649.1 | JF818531.1 | AF544687.1 | JN702320.1 |
| *Liolaemus signifier** | | | JN683118.1 | |
| *Mabuya mabouya* | | | | |
| *Oplurus cyclurus* | GU457850.1 | GU432597.1 | EU099679.1 | JN702378.1 |
| *Petracola ventrimaculatus* | | | AY507910.1 | |
| *Phrynosoma modestum* | DQ385325.1 | KR360318.1 | | KR359901.1 |
| *Plestiodon fasciatus* | HQ876228.1 | JF818524.1 | HQ655218.1 | JN702435.1 |
| *Polychrus marmoratus* | HQ876222.1 | JF818569.1 | AY987983.1 | JN702359.1 |
| *Pristidactylus scapulatus** | JF806025.1 | JF818559.1 | KT342956.1 | JN702459.1 |
| *Pseudopus apodus* | GU457851.1 | GU432599.1 | | |
| *Python molurus* | EU402658.1 | JN881207.1 | GQ225667.1 | JN702316.1 |
| *Rena humilis* | EU402648.1 | GU432633.1 | AY099979.1 | JN702409.1 |
| *Rhineura floridana* | GU457878.1 | GU432628.1 | AY444021.1 | JN702310.1 |
| *Sphenodon punctatus* | GU457846.1 | GU432592.1 | AF039483.1 | JN702443.1 |
| *Stenocercus scapularis** | HQ876224.1 | JF818571.1 | | JN702380.1 |

| | | | | |
|---|---|---|---|---|
| *Teius teyou* | JN654803.1 | JN881211.1 | | JN702400.1 |
| *Timon lepidus* | | | EF632290.1 | |
| *Trioceros jacksonii* | KC507666.1 | | AF137528.1 | |
| *Trogonophis wiegmanni* | GU457879.1 | GU432629.1 | AY444025.1 | JN702395.1 |
| *Tupinambis teguixin* | JN654806.1 | JN881217.1 | | JN702389.1 |
| *Uromastyx aegyptia* | | | AF137531.1 | |
| *Varanus albigularis* | JQ845033.1 | | | |
| *Varanus salvator* | EU402618.1 | GU432610.1 | AF435017.1 | JN702430.1 |
| *Xantusia vigilis* | EU402620.1 | JF818525.1 | EU116833.1 | JN702337.1 |
| *Xenopeltis unicolor* | EU402668.1 | GU432635.1 | AF544689.1 | JN702383.1 |
| *Xenosaurus grandis* | GU457858.1 | GU432606.1 | AY662567.1 | JN702341.1 |

| Taxon | NGFB | NTF3 | PDC | R35 |
|---|---|---|---|---|
| *Afrotyphlops punctatus* | | GU902567.1 | | |
| *Amphisbaena alba* | GU432741.1 | | HQ426249.1 | |
| *Anilius scytale* | EU437988.1 | AY988055.1 | | HQ876355.1 |
| *Bipes biporus* | JN662833.1 | JN568335.1 | | HQ876353.1 |
| *Blanus cinereus* | | EU108015.1 | | |
| *Broadleysaurus major* | | EU636222.1 | | HM161062.1 |
| *Celestus enneagrammus* | GU432719.1 | GU456009.1 | | JN703087.1 |
| *Coleonyx variegatus* | JF818314.1 | JF804539.1 | EF534817.1 | HQ876371.1 |
| *Cordylus niger* | | | | KT941339.1 |
| *Crotaphytus collaris* | JF818338.1 | JF804542.1 | | JF804586.1 |
| *Cylindrophis ruffus* | EU437999.1 | EU390915.1 | | JF804588.1 |
| *Dactylocnemis pacificus* | | | GU459586.1 | |
| *Dibamus* | GU432728.1 | JF804544.1 | HQ426251.1 | |
| *Elgaria multicarinata* | GU432720.1 | GU456010.1 | | HQ876338.1 |
| *Gekko gecko* | EU437977.1 | EU390898.1 | EF534854.1 | HQ876378.1 |
| *Heloderma suspectum* | GU432722.1 | GU456012.1 | HQ426254.1 | HQ876340.1 |
| *Hoplocercus spinosus* | | | | |
| *Iguana iguana* | | HM352530.1 | | KR350699.1 |
| *Lacerta viridis* | GU432740.1 | GU456031.1 | | JF804593.1 |
| *Lanthanotus borneensis* | GU432725.1 | GU456015.1 | | |
| *Leiocephalus carinatus* | | AY987999.1 | | KU979291.1 |
| *Lichanura trivirgata* | EU438013.1 | DQ465578.1 | | HQ876361.1 |
| *Liolaemus signifier** | | | | |
| *Mabuya mabouya* | JF498230.1 | | | KJ574880.1 |
| *Oplurus cyclurus* | GU432716.1 | GU456006.1 | | HQ876332.1 |
| *Petracola ventrimaculatus* | | | | |
| *Phrynosoma modestum* | KR360303.1 | KR360083.1 | | KJ124012.1 |
| *Plestiodon fasciatus* | JF498300.1 | JF804547.1 | | HQ907629.1 |
| *Polychrus marmoratus* | JF818355.1 | JF804564.1 | | HQ876335.1 |
| *Pristidactylus scapulatus** | JF818345.1 | JF804565.1 | | JF804601.1 |
| *Pseudopus apodus* | GU432717.1 | GU456007.1 | | JN703073.1 |
| *Python molurus* | EU438022.1 | | | JN703057.1 |
| *Rena humilis* | EU438012.1 | EU390928.1 | | HQ876364.1 |
| *Rhineura floridana* | GU432743.1 | GU456034.1 | EU293714.1 | DQ119613.1 |
| *Sphenodon punctatus* | GU432712.1 | GU456002.1 | HQ426257.1 | HQ876320.1 |
| *Stenocercus scapularis** | JF818357.1 | JF804570.1 | | HQ876337.1 |
| *Teius teyou* | JN662830.1 | JN568323.1 | | JN568511.1 |
| *Timon lepidus* | | | KX080818.1 | |
| *Trioceros jacksonii* | | AY988006.1 | | |
| *Trogonophis wiegmanni* | GU432744.1 | | | HQ876354.1 |

| | | | | |
|---|---|---|---|---|
| *Tupinambis teguixin* | JN662831.1 | JN568333.1 | | JN568496.1 |
| *Uromastyx aegyptia* | | | | |
| *Varanus albigularis* | | JQ844940.1 | | |
| *Varanus salvator* | EU437981.1 | EU390902.1 | | JN568500.1 |
| *Xantusia vigilis* | EU437983.1 | EU390904.1 | HQ426258.1 | HQ876351.1 |
| *Xenopeltis unicolor* | EU438032.1 | DQ465562.1 | | |
| *Xenosaurus grandis* | GU432724.1 | GU456014.1 | | JN703069.1 |

| Taxon | RAG1 | ND2 | ZEB2 | FSHR |
|---|---|---|---|---|
| *Afrotyphlops punctatus* | GU902645.1 | HQ113933.1 | | |
| *Amphisbaena alba* | AY444042.1 | FJ441949.1 | | |
| *Anilius scytale* | EU402834.1 | NC_014343.1 | EU390857.1 | EU391110.1 |
| *Bipes biporus* | HQ876445.1 | NC_006287.1 | JN568546.1 | |
| *Blanus cinereus* | EU108523.1 | NC_012433.1 | | |
| *Broadleysaurus major* | | KF717422.1 | | |
| *Celestus enneagrammus* | GU457976.1 | AF085607.1 | GU456231.1 | GU455975.1 |
| *Coleonyx variegatus* | HQ876448.1 | NC_008774.1 | JF804620.1 | JF804389.1 |
| *Cordylus niger* | KT941374.1 | AY519699.1 | | |
| *Crotaphytus collaris* | JF806206.1 | U82681.1 | JF804623.1 | JF804392.1 |
| *Cylindrophis ruffus* | EU402842.1 | AB179619.1 | EU390866.1 | EU391120.1 |
| *Dactylocnemis pacificus* | GU459392.1 | GU459794.1 | | |
| *Dibamus novaeguineae* | GU457986.1 | FJ195390.1 | | |
| *Elgaria multicarinata* | GU457977.1 | AF085620.1 | GU456232.1 | GU455976.1 |
| *Gekko gecko* | EU402824.1 | NC_007627.1 | EU390847.1 | EU391100.1 |
| *Heloderma suspectum* | GU457979.1 | NC_008776.1 | GU456234.1 | GU455978.1 |
| *Hoplocercus spinosus* | AY662592.1 | U82683.1 | | |
| *Iguana iguana* | KR350706.1 | JF498123.1 | | |
| *Lacerta viridis* | GU457997.1 | NC_008328.1 | GU456253.1 | GU455996.1 |
| *Lanthanotus borneensis* | GU457982.1 | AF407541.1 | GU456237.1 | GU455981.1 |
| *Leiocephalus carinatus* | AY662598.1 | AF049864.1 | | |
| *Lichanura trivirgata* | EU402852.1 | GQ200595.1 | EU390880.1 | EU391134.1 |
| *Liolaemus signifier** | | AF099266.1 | | |
| *Mabuya mabouya* | | JF498123.1 | | |
| *Oplurus cyclurus* | GU457973.1 | | GU456228.1 | GU455972.1 |
| *Petracola ventrimaculatus* | | | | |
| *Phrynosoma modestum* | KR360097.1 | AY297484.1 | KR360287.1 | KR360399.1 |
| *Plestiodon fasciatus* | HQ876444.1 | AY607299.1 | JF804627.1 | JF804396.1 |
| *Polychrus marmoratus* | HQ876438.1 | NC_012839.1 | JF804644.1 | JF804413.1 |
| *Pristidactylus scapulatus** | JF806210.1 | AF528732.1 | JF804645.1 | JF804414.1 |
| *Pseudopus apodus* | GU457974.1 | AF085623.1 | GU456229.1 | GU455973.1 |
| *Python molurus* | | NC_015812.1 | | JN702974.1 |
| *Rena humilis* | EU402851.1 | AB079597.1 | | |
| *Rhineura floridana* | GU458000.1 | NC_006282.1 | GU456256.1 | GU455999.1 |
| *Sphenodon punctatus* | GU457969.1 | KP996625.1 | GU456224.1 | GU455968.1 |
| *Stenocercus scapularis** | HQ876440.1 | DQ080223.1 | JF804650.1 | JF804419.1 |
| *Teius teyou* | JN654865.1 | JN700172.1 | JN568560.1 | JN568475.1 |
| *Timon lepidus* | EF110996.1 | DQ902256.1 | | |
| *Trioceros jacksonii* | JQ073211.1 | AF448753.1 | | |
| *Trogonophis wiegmanni* | GU458001.1 | AY662542.1 | GU456257.1 | GU456000.1 |

| | | | | |
|---|---|---|---|---|
| *Tupinambis teguixin* | JN654868.1 | JN700173.1 | JN568569.1 | JN568481.1 |
| *Uromastyx aegyptia* | | AB619817.1 | | |
| *Varanus albigularis* | KT721075.1 | HQ234911.1 | | |
| *Varanus salvator* | EU402828.1 | NC_010974.1 | EU390851.1 | EU391104.1 |
| *Xantusia vigilis* | EU402830.1 | EU130279.1 | EU390853.1 | EU391106.1 |
| *Xenopeltis unicolor* | EU402870.1 | NC_007402.1 | EU390897.1 | EU391152.1 |
| *Xenosaurus grandis* | GU457981.1 | U71333.2 | GU456236.1 | GU455980.1 |

| Taxon | TRAF6 | FSTL5 | 12S | 16S |
|---|---|---|---|---|
| *Afrotyphlops punctatus* | | | HQ113893.1 | |
| *Amphisbaena alba* | | | | KR815887 |
| *Anilius scytale* | EU391058.1 | EU402785.1 | NC_014343.1 | NC_014343.1 |
| *Bipes biporus* | JN568459.1 | JN654834.1 | NC_006287.1 | NC_006287.1 |
| *Blanus cinereus* | | | NC_012433.1 | NC_012433.1 |
| *Broadleysaurus major* | | | AJ416921.1 | AJ416922.1 |
| *Celestus enneagrammus* | GU456154.1 | GU457946.1 | | |
| *Coleonyx variegatus* | JF804342.1 | JF806108.1 | NC_008774.1 | NC_008774.1 |
| *Cordylus niger* | | | HQ167106.1 | HQ167217.1 |
| *Crotaphytus collaris* | JF804345.1 | JF806134.1 | L40439.1 | L41443.1 |
| *Cylindrophis ruffus* | EU391069.1 | EU402795.1 | AB179619.1 | AB179619.1 |
| *Dactylocnemis pacificus* | | | | GU459993.1 |
| *Dibamus novaeguineae* | | | FJ195390.1 | FJ195390.1 |
| *Elgaria multicarinata* | GU456155.1 | GU457947.1 | AY649110.1.1 | |
| *Gekko gecko* | EU391048.1 | EU402775.1 | NC_007627.1 | NC_007627.1 |
| *Heloderma suspectum* | GU456157.1 | GU457948.1 | NC_008776.1 | NC_008776.1 |
| *Hoplocercus spinosus* | | | | |
| *Iguana iguana* | | | NC_002793.1 | NC_002793.1 |
| *Lacerta viridis* | GU456176.1 | GU457964.1 | NC_008328.1 | NC_008328.1 |
| *Lanthanotus borneensis* | GU456160.1 | GU457951.1 | | |
| *Leiocephalus carinatus* | | | | |
| *Lichanura trivirgata* | EU391083 | | GQ200595.1 | GQ200595.1 |
| *Liolaemus signifier** | | | KF969090.1 | |
| *Mabuya mabouya* | | | JF497871.1 | AY070357.1 |
| *Oplurus cyclurus* | GU456151.1 | GU457943.1 | U39585.1 | |
| *Petracola ventrimaculatus* | | | KJ948193.1 | KJ948144.1 |
| *Phrynosoma modestum* | KR360111.1 | KR360139.1 | DQ385397.1 | L41455.1 |
| *Plestiodon fasciatus* | JF804349.1 | JF806110.1 | AY315505.1 | AY308199.1 |
| *Polychrus marmoratus* | JF804366.1 | JF806144.1 | NC_012839.1 | NC_012839.1 |
| *Pristidactylus scapulatus** | JF804367.1 | JF806145.1 | KT342931.1 | |
| *Pseudopus apodus* | GU456152.1 | GU457944.1 | AF380955.1 | JX987420.1 |
| *Python molurus* | JN703148.1 | EU402816.1 | NC_015812.1 | NC_015812.1 |
| *Rena humilis* | | | AB079597.1 | AB079597.1 |
| *Rhineura floridana* | GU456179.1 | GU457967.1 | NC_006282.1 | NC_006282.1 |
| *Sphenodon punctatus* | GU456147.1 | GU457940.1 | KP996625.1 | KP996625.1 |
| *Stenocercus scapularis** | JF804372.1 | JF806148.1 | | L41481.1 |
| *Teius teyou* | JN568447.1 | JN654844.1 | AY046461.1 | AY046503.1 |
| *Timon lepidus* | | | GQ142071.1 | AF378949.1 |
| *Trioceros jacksonii* | | | DQ397240.1 | JN165401.1 |
| *Trogonophis wiegmanni* | GU456180.1 | GU457968.1 | EF545732.1 | EF545735.1 |

| | | | | |
|---|---|---|---|---|
| *Tupinambis teguixin* | JN568441.1 | JN654848.1 | KU894533.1 | KU894543 |
| *Uromastyx aegyptia* | | | FJ639656.1 | FJ639621.1 |
| *Varanus albigularis* | | | | |
| *Varanus salvator* | EU391052.1 | JF806113.1 | NC_010974.1 | NC_010974.1 |
| *Xantusia vigilis* | EU391054.1 | EU402781.1 | AY218042.1 | KC621482.1 |
| *Xenopeltis unicolor* | EU391099.1 | EU402823.1 | NC_007402.1 | NC_007402.1 |
| *Xenosaurus grandis* | GU456159.1 | GU457950.1 | | |

**Supplementary Table 2**. Estimates of posterior divergence times—lower and upper ranges of 95% highest posterior densities (HPD)—for five selected nodes from distinct regions of the posterior trees under different tree prior parameters. Even with a relatively large tree and abundant fossil calibration data, divergence times are, in general, widely distributed over a broad range of time, especially at the oldest nodes (root and ingroup) when compared to nodes lying more crownward on the tree (Gekkota, Toxicofera and Iguania). This indicates that even data sets with considerable calibration information may not escape deep root attraction (DRA). Applying a moderate penalty for ghost lineages on diversification or fossil sampling priors, such as the turnover rate *r* [30], provides considerable improvement on reducing the gap between divergence time estimates and fossil calibration data, also providing more precise divergence times. Abbreviations: Max., Maximum value for 95% HPD for divergence time; Min., Minimum value for 95% HPD for divergence time; *r*, turnover rate. Range represents the total range of the 95% HPD.

| | **Mr. Bayes** | | | | | | **BEAST2** | | | | | |
|---|---|---|---|---|---|---|---|---|---|---|---|---|
| | ***r*=beta(1,1)** | | | ***r*=beta(1,100)** | | | ***r*=Uniform(-∞;∞)** | | | ***r*=beta(1,100)** | | |
| | **Min.** | **Max.** | **Range** | **Min.** | **Max.** | **Range** | **Min.** | **Max.** | **Diff.** | **Min.** | **Max.** | **Range** |
| **Root** | 350.5 | 403.64 | 53.14 | 318 | 332.31 | 14.31 | 333.75 | 398.79 | 65.04 | 331.83 | 392.71 | 60.88 |
| **Diapsida (ingroup)** | 344.51 | 392.8 | 48.29 | 307.7 | 325.04 | 17.34 | 329.54 | 390.13 | 60.59 | 326.69 | 383.09 | 56.4 |
| **Gekkota** | 155.12 | 197.5 | 42.38 | 150 | 171.15 | 21.15 | 150.36 | 204.83 | 54.47 | 150 | 202.2 | 52.2 |
| **Toxicofera** | 171.11 | 193.29 | 22.18 | 167.11 | 175.49 | 8.38 | 173.86 | 193.16 | 19.3 | 172.68 | 189.97 | 17.29 |
| **Iguania** | 113.76 | 156.86 | 43.1 | 88.23 | 121.06 | 32.83 | 95.69 | 158.45 | 62.76 | 92.9 | 158.98 | 66.08 |

Supplementary Table 3. Pooled sample of relative phenotypic rates at each time bin for early diapsids.

| Epoch | n | mean | sd | min | Q1 | median | Q3 | max |
|---|---|---|---|---|---|---|---|---|
| **Cisuralian** | 21 | 2.411 | 1.986 | 0.076 | 0.323 | 2.34 | 3.37 | 5.97 |
| **Guadalupian** | 18 | 1.812 | 1.789 | 0.06 | 0.725 | 1.495 | 1.968 | 7.45 |
| **Lopingian** | 23 | 1.557 | 1.108 | 0.018 | 0.68 | 1.63 | 2.01 | 4.81 |
| **Early Triassic** | 18 | 1.868 | 0.941 | 0.642 | 1.292 | 1.7 | 2.513 | 4.24 |
| **Middle Triassic** | 29 | 2.629 | 2.643 | 0.001 | 0.698 | 1.97 | 3.33 | 13.2 |

Supplementary Table 4. Pooled sample of relative phenotypic rates at each time bin for lepidosaurs.

| Epoch | n | mean | sd | min | Q1 | median | Q3 | Max |
|---|---|---|---|---|---|---|---|---|
| **J1 (Hatte-Aelen)** | 16 | 1.348 | 1.233 | 0.029 | 0.505 | 0.932 | 1.862 | 4.05 |
| **J2 (Bajo-Tithon)** | 22 | 2.498 | 6.455 | 0.178 | 0.318 | 0.445 | 0.814 | 26.6 |
| **Early Cretaceous** | 46 | 0.658 | 0.887 | 0.081 | 0.196 | 0.308 | 0.698 | 3.89 |
| **LK1 (Ceno-Sant)** | 20 | 0.67 | 0.504 | 0.089 | 0.23 | 0.561 | 1.038 | 1.44 |
| **LK2 (Camp-Maast)** | 21 | 1.277 | 1.048 | 0.066 | 0.581 | 0.989 | 1.64 | 3.91 |
| **Paleocene** | 12 | 0.88 | 0.891 | 0.027 | 0.472 | 0.658 | 0.931 | 3.29 |
| **Early Eocene** | 24 | 0.778 | 1.065 | 0.021 | 0.06 | 0.446 | 0.998 | 4.99 |
| **Extant** | 47 | 0.564 | 0.36 | 0.272 | 0.378 | 0.441 | 0.632 | 2.41 |

Supplementary Table 5. Pooled sample of relative molecular rates at each time bin for lepidosaurs.

| Epoch | n | mean | sd | min | Q1 | median | Q3 | Max |
|---|---|---|---|---|---|---|---|---|
| **J1 (Hatte-Aelen)** | 16 | 1.624 | 1.447 | 0.292 | 0.409 | 1.095 | 2.158 | 4.49 |
| **J2 (Bajo-Tithon)** | 22 | 0.349 | 0.215 | 0.078 | 0.127 | 0.4 | 0.443 | 0.825 |
| **Early Cretaceous** | 46 | 0.432 | 0.301 | 0.06 | 0.181 | 0.396 | 0.558 | 1.39 |
| **LK1 (Ceno-Sant)** | 20 | 0.574 | 0.232 | 0.099 | 0.494 | 0.588 | 0.714 | 1.01 |
| **LK2 (Camp-Maast)** | 21 | 0.731 | 0.96 | 0.151 | 0.235 | 0.45 | 0.7 | 4.39 |
| **Paleocene** | 12 | 1.13 | 1.053 | 0.017 | 0.35 | 0.75 | 1.528 | 3.34 |
| **Early Eocene** | 24 | 1.562 | 2.159 | 0.201 | 0.496 | 0.728 | 2.277 | 10.7 |
| **Extant** | 47 | 0.955 | 0.497 | 0.002 | 0.638 | 0.833 | 1.26 | 2.44 |

Supplementary Table 6. Pooled sample of bootstraped mophological disparity values at each time bin for early diapsids.

| Epoch | n | mean | sd | min | Q1 | median | Q3 | max |
|---|---|---|---|---|---|---|---|---|
| **Cisuralian** | 100 | 18.503 | 0.294 | 17.583 | 18.332 | 18.502 | 18.694 | 19.122 |
| **Early Triassic** | 100 | 17.657 | 0.875 | 13.843 | 17.121 | 17.892 | 18.202 | 19.32 |
| **Guadalupian** | 100 | 17.257 | 0.907 | 13.908 | 16.728 | 17.584 | 17.962 | 18.851 |
| **Lopingian** | 100 | 18.372 | 0.407 | 17.214 | 18.108 | 18.404 | 18.674 | 19.109 |
| **Middle Triassic** | 100 | 19.186 | 0.21 | 18.71 | 19.05 | 19.185 | 19.316 | 19.59 |

Supplementary Table 7. Pooled sample of bootstraped mophological disparity values at each time bin for lepidosaurs.

| Epoch | n | mean | sd | min | Q1 | median | Q3 | max |
|---|---|---|---|---|---|---|---|---|
| **J1 (Hetta-Aalen)** | 100 | 18.052 | 0.763 | 15.693 | 17.65 | 18.219 | 18.56 | 19.162 |
| **J2 (Bajo-Titho)** | 100 | 18.14 | 0.657 | 15.827 | 17.837 | 18.297 | 18.585 | 19.22 |
| **Early Cretaceous** | 100 | 18.259 | 0.332 | 17.279 | 18.01 | 18.269 | 18.473 | 19.025 |
| **LK1 (Ceno-Sant)** | 100 | 18.282 | 0.706 | 16.176 | 17.852 | 18.386 | 18.751 | 19.589 |
| **LK2 (Camp-Maast)** | 100 | 18.912 | 0.28 | 17.899 | 18.706 | 18.934 | 19.11 | 19.485 |
| **Paleocene** | 100 | 17.946 | 0.777 | 14.936 | 17.512 | 18.09 | 18.578 | 19.143 |
| **Eocene** | 100 | 18.872 | 0.299 | 17.669 | 18.726 | 18.902 | 19.092 | 19.419 |
| **Extant** | 100 | 19.965 | 0.115 | 19.507 | 19.908 | 19.972 | 20.047 | 20.188 |

Supplementary Table 8. Pairwise t-tests (two-sided) of phenotypic rates across time bins for early diapsids. Bold values are significant (p ≤ 0.05).

| | Cisuralian | Guadalupian | Lopingian | Early Triassic |
|---|---|---|---|---|
| **Guadalupian** | 0.861716 | NA | NA | NA |
| **Lopingian** | 0.858289 | 0.892796 | NA | NA |
| **Early Triassic** | 0.934988 | 0.861716 | 0.858289 | NA |
| **Middle Triassic** | 0.892796 | 0.858289 | 0.858289 | 0.892796 |

Supplementary Table 9. Pairwise Wilcoxon rank sum (Mann-Whitney) tests (two-sided) of phenotypic rates across time bins for lepidosaurs. Values in bold are significant (p ≤ 0.05).

| | J1 (Hatte-Aelen) | J2 (Bajo-Tithon) | Early Cretaceous | LK1 (Ceno-Sant) | LK2 (Camp-Maast) | Paleocene | Early Eocene |
|---|---|---|---|---|---|---|---|
| **J2 (Bajo-Tithon)** | 0.417792 | NA | NA | NA | NA | NA | NA |
| **Early Cretaceous** | 0.212601 | 0.212601 | NA | NA | NA | NA | NA |
| **LK1 (Ceno-Sant)** | 0.43402 | 0.738575 | 0.494622 | NA | NA | NA | NA |
| **LK2 (Camp-Maast)** | 0.986053 | 0.220719 | **0.038567** | 0.288567 | NA | NA | NA |
| **Paleocene** | 0.52818 | 0.52818 | 0.350619 | 0.986053 | 0.350619 | NA | NA |
| **Early Eocene** | 0.350619 | 0.6128 | 0.995064 | 0.661069 | 0.136851 | 0.83368 | NA |
| **Extant** | 0.136851 | 0.995064 | 0.136851 | 0.986053 | **0.038567** | 0.243536 | 0.876402 |

Supplementary Table 10. Pairwise Wilcoxon rank sum (Mann-Whitney) tests (two-sided) of molecular rates across time bins for lepidosaurs. Bold values are significant (p ≤ 0.05).

| | J1 (Hatte-Aelen) | J2 (Bajo-Tithon) | Early Cretaceous | LK1 (Ceno-Sant) | LK2 (Camp-Maast) | Paleocene | Early Eocene |
|---|---|---|---|---|---|---|---|
| **J2 (Bajo-Tithon)** | **0.003508** | NA | NA | NA | NA | NA | NA |
| **Early Cretaceous** | **0.003131** | 0.48788 | NA | NA | NA | NA | NA |
| **LK1 (Ceno-Sant)** | 0.133946 | **0.008481** | **0.034769** | NA | NA | NA | NA |
| **LK2 (Camp-Maast)** | **0.034338** | 0.168577 | 0.431729 | 0.382623 | NA | NA | NA |
| **Paleocene** | 0.431729 | 0.089725 | 0.089725 | 0.597384 | 0.286101 | NA | NA |
| **Early Eocene** | 0.902304 | **0.000186** | **0.000258** | 0.203337 | **0.032357** | 0.523821 | NA |
| **Extant** | 0.56262 | **2.32E-06** | **6.35E-07** | **0.003508** | **0.003508** | 0.775915 | 0.837841 |

Supplementary Table 11. Pairwise Wilcoxon rank sum (Mann-Whitney) tests of phenotypic disparity across time bins for early diapsids. Bold values are significant (p ≤ 0.05).

| | Cisuralian | Guadalupian | Lopingian | Early Triassic |
|---|---|---|---|---|
| **Guadalupian** | **1.60E-27** | NA | NA | NA |
| **Lopingian** | **0.026101** | **3.29E-22** | NA | NA |
| **Early Triassic** | **1.12E-16** | **0.001066** | **1.54E-11** | NA |
| **Middle Triassic** | **1.09E-30** | **3.16E-33** | **1.29E-30** | **3.08E-32** |

Supplementary Table 12. Pairwise Wilcoxon rank sum (Mann-Whitney) tests (two-sided) of phenotypic disparity across time bins for lepidosaurs. Bold values are significant (p ≤ 0.05).

| | J1 (Hetta-Aalen) | J2 (Bajo-Titho) | Early Cretaceous | LK1 (Ceno-Sant) | LK2 (Camp-Maast) | Paleocene | Eocene |
|---|---|---|---|---|---|---|---|
| **J2 (Bajo-Titho)** | 0.681771 | NA | NA | NA | NA | NA | NA |
| **Early Cretaceous** | 0.431259 | 0.925053 | NA | NA | NA | NA | NA |
| **LK1 (Ceno-Sant)** | 0.08743 | 0.19504 | 0.312467 | NA | NA | NA | NA |
| **LK2 (Camp-Maast)** | **1.52E-21** | **1.08E-21** | **9.83E-26** | **1.20E-12** | NA | NA | NA |
| **Paleocene** | 0.291441 | 0.102794 | **0.017518** | **0.006147** | **1.01E-22** | NA | NA |
| **Eocene** | **3.06E-20** | **3.06E-20** | **1.10E-23** | **1.39E-11** | 0.438097 | **1.66E-21** | NA |
| **Extant** | **1.12E-33** | **1.12E-33** | **1.12E-33** | **1.12E-33** | **1.12E-33** | **1.12E-33** | **1.12E-33** |

# Supplementary References


1 Goloboff, P. A., Torres, A. & Arias, J. S. Weighted parsimony outperforms other methods of phylogenetic inference under models appropriate for morphology. *Cladistics* **34**, 407-437 (2017).

2 Puttick, M. N., O'Reilly, J. E., Pisani, D. & Donoghue, P. C. Probabilistic methods outperform parsimony in the phylogenetic analysis of data simulated without a probabilistic model. *Palaeontology* **62**, 1-17 (2019).

3 Cocude-Michel, M. Les Rhynchocephales et les Sauriens des calcaires lithographiques (Jurassique Superieur) d'Europe occidentale. *Nouvelles archives du Muséum d'Histoire Naturelle de Lyon* **7**, 1-187 (1963).

4 Dupret, V. The pleurosaurs: anatomy and phylogeny. *Revue de Paléobiologie, Genève* **9**, 61-80 (2004).

5 Schweigert, G. Ammonite biostratigraphy as a tool for dating Upper Jurassic lithographic limestones from South Germany–first results and open questions. *Neues Jahrbuch für Geologie und Paläontologie-Abhandlungen* **245**, 117-125 (2007).

6 Reynoso, V.-H. *Early Cretaceous lepidosaurs (Reptilia: Diapsida) from Central Mexico and the phylogeny of lepidosauromorphs* PhD thesis, McGill University (1996).

7 Meyer, H. v. Neue fossile Reptilien aus der Ordnung der Saurier. *Nova acta physico-medica Academiae Caesareae Leopoldino-Carolinae Naturae Curiosum* **15**, 171-200 (1831).

8 Münster, G. Ueber einige Petrefakten-Kunde in den lithographischen Schiefern von Baiern. *Neues Jahrbuch für Mineralogie, Geologie und Paläontologie*, 676-682 (1839).

9 Fitzinger, L. *Systema Reptilium* (Braumüller et Seidel, 1843).

10 Wagner, J. A. Vergleichung der urweltlichen Fauna des lithographischen Schiefers von Cirin mit den gleichnamigen Ablagerungen im Frankischen Jura. *Gelehrten Anzeiger der Königlich Bayerischen Akademie der Wissenschaften* **48**, 390-391 (1860).

11 Zittel, K. (Druck und Verlag Von R. Oldenbourg, 1887).

12 Watson, D. M. S. *Pleurosaurus* and the homologies of the bones of the temporal region of the lizard's skull. *Annals and Magazine of Natural History* **14**, 84-95 (1914).

13 Broili, F. Ueber ein neu entdecktes Exemplar von *Pleurosaurus goldfussi* H. von Meyer aus dem Malm Frankens. *Forschung und Fortschritt* **2**, 105-107 (1926).

14 Huene, F. v. Revision der Gattung *Pleurosaurus* auf Grund neuer und alter Funde. *Palaeontog. Abt. A Palaeozool.-Strat.*, 167-200 (1952).

15 Hoffstetter, R. in *Traité de Paleontologie* (ed J. Piveteau) 556–576 (Masson et Cie, 1955).

16 Cocude-Michel, M. Revision des Rhynchocephales de la collection du Musee Teyler de Haarlem (Pays-Bas) I. *Proceedings of the Koninklijke Nederlandse Akademie van Wetenschappen. Series B (Physicai Sciences)* **34**, 538-546 (1967).

17 Kuhn, O. *Handbuch der Paläoherpetologie-Part 9: Proganosauria, Bolosauria, Placodontia, Araeoscelidia, Trilophosauria, Weigeltisauria, Millerosauria, Rhynchocephalia, Protorosauria* (Verlag Dr. Friedrich Pfeil, 1969).

18 Carroll, R. L. Evolutionary constraints in aquatic diapsid reptiles. *Spec. Pap. Palaeontol.* **33**, 145-155 (1985).

19 Carroll, R. L. A pleurosaur from the Lower Jurassic and the taxonomic position of the Sphenodontida. *Palaeontog. Abt. A Palaeozool.-Strat.* **189**, 1-28 (1985).

20 Carroll, R. L. & Wild, R. in *In the shadow of the dinosaurs: early Mesozoic tetrapods* (eds N. C. Fraser & H. D. Sues) 70-83 (Cambridge University Press, 1994).

21 Jones, M. E. H. Skull shape and feeding strategy in *Sphenodon* and other Rhynchocephalia (Diapsida: Lepidosauria). *J. Morphol.* **269**, 945-966 (2008).
22 Evans, S. E. & Jones, M. E. H. in *New Aspects of Mesozoic Biodiversity* Vol. 132 *Lecture Notes in Earth Sciences* (ed Saswati Bandyopadhyay) Ch. 2, 27-44 (Springer Berlin Heidelberg, 2010).
23 Müller, J. *et al.* Eocene lizard from Germany reveals amphisbaenian origins. *Nature* **473**, 364-367 (2011).
24 Tałanda, M. Cretaceous roots of the amphisbaenian lizards. *Zool. Scr.* **45**, 1-8 (2016).
25 Dashzeveg, D. *et al.* New Stratigraphic Subdivision, Depositional Environment, and Age Estimate for the Upper Cretaceous Djadokhta Formation, Southern Ulan Nur Basin, Mongolia. *Am. Mus. Novit.*, 1-31 (2005).
26 Dingus, L. *et al.* The Geology of Ukhaa Tolgod (Djadokhta Formation, Upper Cretaceous, Nemegt Basin, Mongolia). *Am. Mus. Novit.*, 1-40 (2008).
27 Gao, K.-Q. & Norell, M. A. Taxonomic composition and systematics of Late Cretaceous lizard assemblages from Ukhaa Tolgod and adjacent localities, Mongolian Gobi Desert. *Bull. Am. Mus. Nat. Hist.* **249**, 1-118 (2000).
28 Conrad, J. L., Rieppel, O., Gauthier, J. A. & Norell, M. A. Osteology of *Gobiderma pulchrum* (Monstersauria, Lepidosauria, Reptilia). *Bull. Am. Mus. Nat. Hist.*, 1-88 (2011).
29 Borsuk-Białynicka, M. Anguimorphans and related lizards from the Late Cretaceous of the Gobi Desert, Mongolia. *Palaeontol. Pol.* **46**, 5-105 (1984).
30 Ronquist, F., Lartillot, N. & Phillips, M. J. Closing the gap between rocks and clocks using total-evidence dating. *Phil. Trans. R. Soc. B* **371**, 20150136 (2016).
