# Supplementary material for: Megaevolutionary dynamics and the timing of evolutionary innovation in reptiles
Source: Nat Commun. 2020 Jul 3;11:3322. doi: 10.1038/s41467-020-17190-9 (PMC7335191; doi:10.1038/s41467-020-17190-9)
Supplement: Supplementary file 3 — Reporting Summary [file 41467_2020_17190_MOESM3_ESM.pdf]

nature research

Corresponding author(s): Tiago R. Simões

Last updated by author(s): Jun 10, 2020

Reporting Summary

Nature Research wishes to improve the reproducibility of the work that we publish. This form provides structure for consistency and transparency in reporting. For further information on Nature Research policies, see Authors & Referees and the Editorial Policy Checklist .

Statistics

For all statistical analyses, confirm that the following items are present in the figure legend, table legend, main text, or Methods section.

n/a	Confirmed	
☐	☒	The exact sample size (*n*) for each experimental group/condition, given as a discrete number and unit of measurement
☐	☒	A statement on whether measurements were taken from distinct samples or whether the same sample was measured repeatedly
☐	☒	The statistical test(s) used AND whether they are one- or two-sided<br>*Only common tests should be described solely by name; describe more complex techniques in the Methods section.*
☒	☐	A description of all covariates tested
☐	☒	A description of any assumptions or corrections, such as tests of normality and adjustment for multiple comparisons
☐	☒	A full description of the statistical parameters including central tendency (e.g. means) or other basic estimates (e.g. regression coefficient) AND variation (e.g. standard deviation) or associated estimates of uncertainty (e.g. confidence intervals)
☐	☒	For null hypothesis testing, the test statistic (e.g. *F*, *t*, *r*) with confidence intervals, effect sizes, degrees of freedom and *P* value noted<br>*Give P values as exact values whenever suitable.*
☐	☒	For Bayesian analysis, information on the choice of priors and Markov chain Monte Carlo settings
☒	☐	For hierarchical and complex designs, identification of the appropriate level for tests and full reporting of outcomes
☒	☐	Estimates of effect sizes (e.g. Cohen's *d*, Pearson's *r*), indicating how they were calculated

Our web collection on statistics for biologists contains articles on many of the points above.

Software and code

Policy information about availability of computer code

Data collection	No software was used for data collection
Data analysis	Our data was compiled in Mesquite (v. 3.04); molecular alignments were performed with MAFFT (Multiple Sequence Alignment Software Version 7); our analysis were performed in the phylogenetic software Mr, Bayes (v. 3.2.6) and BEAST2. Disparity analyses, morphospace, and tree plots were produced using R, using core functions and additional functions available in the packages ggplot2, ggtree, Claddis and disparity.

For manuscripts utilizing custom algorithms or software that are central to the research but not yet described in published literature, software must be made available to editors/reviewers. We strongly encourage code deposition in a community repository (e.g. GitHub). See the Nature Research guidelines for submitting code & software for further information.

Data

Policy information about availability of data

All manuscripts must include a data availability statement. This statement should provide the following information, where applicable:

- Accession codes, unique identifiers, or web links for publicly available datasets
- A list of figures that have associated raw data
- A description of any restrictions on data availability

All morphological and molecular data generated and analyzed, along with trees, log files, prior parameters and posterior parameter values described in the results and figures, and detailed results of statistical tests is available online as Supplementary Data files S1-S5 at Harvard's Dataverse Repository (DOI: 10.7910/DVN/ZONWD0)

Field-specific reporting

Please select the one below that is the best fit for your research. If you are not sure, read the appropriate sections before making your selection.

☐ Life sciences ☐ Behavioural & social sciences ☒ Ecological, evolutionary & environmental sciences

For a reference copy of the document with all sections, see nature.com/documents/nr-reporting-summary-flat.pdf

Ecological, evolutionary & environmental sciences study design

All studies must disclose on these points even when the disclosure is negative.

Study description	We utilized morphological (personally collected in museum collections) and molecular (available online) data from all major lineages of diapsid reptiles and utilized recently developed phylogenetic-based macroevolutionary techniques to detect rates of morphological and molecular evolution, besides shifts in morphological disparity across geological time.
Research sample	The collected data from 47 species of extant lepidosaurs and 91 species of fossil reptiles from over 50 different museum collections across the world, spanning in geological time from 307 myr to the present.
Sampling strategy	Our samples were obtained in a way to maximize the representation of the modern diversity of reptiles, as well as the representation of fossil lineages, focusing on the earliest and most complete fossils from each reptile clades studied here.
Data collection	Data was collected using digital photography and CT scanning technologies for the morphological data, whereas molecular data was obtained from GenBank.
Timing and spatial scale	N/A
Data exclusions	No data was excluded from the analyses.
Reproducibility	Some of the analyses conducted were repeated multiple times, yielding similar results in terms of posterior parameter distributions.
Randomization	For the morphospace plot, groups were assigned according to their taxonomic classification.
Blinding	Blinding was not relevant to this data set, and set of analyses.

Did the study involve field work? ☐ Yes ☒ No

Reporting for specific materials, systems and methods

We require information from authors about some types of materials, experimental systems and methods used in many studies. Here, indicate whether each material, system or method listed is relevant to your study. If you are not sure if a list item applies to your research, read the appropriate section before selecting a response.

Materials & experimental systems

n/a	Involved in the study	
☒	☐	Antibodies
☒	☐	Eukaryotic cell lines
☐	☒	Palaeontology
☐	☒	Animals and other organisms
☒	☐	Human research participants
☒	☐	Clinical data

Methods

n/a	Involved in the study	
☒	☐	ChIP-seq
☒	☐	Flow cytometry
☒	☐	MRI-based neuroimaging

Palaeontology

Specimen provenance	The provenance of every single specimens used here is available on the supplementary information, as well as on the supplementary information of ref. 17
Specimen deposition	All specimens included here were observed in publicly accessible museum and university collections across different countries.
Dating methods	No new dates were provided

☒ Tick this box to confirm that the raw and calibrated dates are available in the paper or in Supplementary Information.

Animals and other organisms

Policy information about studies involving animals; ARRIVE guidelines recommended for reporting animal research

Laboratory animals	No laboratory animals were used
Wild animals	No wild animals were used
Field-collected samples	All personally collected data from specimens of extant species are housed in publicly accessible museum and university collections as dry/skeletonized materials.
Ethics oversight	No ethical approval or guidance was required as all extant specimens had already been euthanized, skeletonized and deposited at institutional collections prior to this project.

Note that full information on the approval of the study protocol must also be provided in the manuscript.
