# Supplementary material for: Megaevolutionary dynamics and the timing of evolutionary innovation in reptiles
Source: Nat Commun. 2020 Jul 3;11:3322. doi: 10.1038/s41467-020-17190-9 (PMC7335191; doi:10.1038/s41467-020-17190-9)
Supplement: Supplementary file 4 — Description of Additional Supplementary Files [file 41467_2020_17190_MOESM4_ESM.pdf]

Description of Additional Supplementary Files

File name: Supplementary Data 1
Description: Data sets for phylogenetic and disparity analyses, including Mr. Bayes blocks within files and XML files for Beast2 analyses.

File name: Supplementary Data 2
Description: Mr. Bayes log files, tree files and stats files.

File name: Supplementary Data 3
Description: BEAST2 trees and log files.

File name: Supplementary Data 4
Description: Prior parameter files (effective priors) for the final analyses.

File name: Supplementary Data 5
Description: Detailed statistical tests.
