# Supplementary material for: Megaevolutionary dynamics and the timing of evolutionary innovation in reptiles
Source: Nat Commun. 2020 Jul 3;11:3322. doi: 10.1038/s41467-020-17190-9 (PMC7335191; doi:10.1038/s41467-020-17190-9)
Supplement: Supplementary file 2 — Peer Review File [file 41467_2020_17190_MOESM2_ESM.pdf]

Reviewers' comments:

Reviewer #1 (Remarks to the Author):

Simoes and colleagues present a study investigating the rates of phenotypic and genetic evolution across diapsids. Using morphological characters based on a previously published phylogenetic dataset (138 taxa, 105 characters) and sequence data from GenBank, they tackle the challenging topic of assessing the presence of adaptive radiations in a phenotypically and taxonomically diverse group of diapsid reptiles. The authors propose that rates of morphological and genetic evolution are largely decoupled and elevated rates are not especially restricted to origins of new bodyplans. Additionally, they find convergent evolution of similar traits through different rates of evolution. The strengths of the study includes the incorporation of many fossils in the dataset, investigation into correlation between phenotypic and molecular rates of evolution, and use of two different software to assess convergence in results. The topics explored in the study is certainly of great interest to the field of evolutionary biology and the taxonomic range of the study is notable.

At the same time, I found the experimental design to be inadequate for addressing the main questions of the study. For example, it analyzes a dataset with very low taxonomic sampling with respect to the study's scope and objectives, makes a number of nested assumptions, does not fully take into account uncertainties, and lack statistical rigor when making claims. Consequently, the approaches used in this study are disjointed from the narrative scope and aims of the study. While I enjoyed reading the paper, I was ultimately not convinced of the robustness and reliability of the results and conclusions being presented. Please see below for my major comments on the paper, followed by more minor, specific comments.

GENERAL COMMENTS

1. The analysis contains many nested assumptions, most of which remain unverified for this dataset/group. While assumptions are unavoidable in any study, here is a compounding list of key assumptions that I have noted from data collection to analysis:

a) Taxonomic sampling contains 47 extant lepidosaur taxa for which both molecular and morphological data could be collected. This does not seem to be sufficient sampling for characterizing such a phenotypically diverse clade. More importantly, it is not clear to me how such a low taxonomic sampling could be used to reliably estimate taxonomic diversification which is used as a criterion for identifying adaptive radiations. Clear justifications for the taxonomic sampling must be provided.

b) Related to (a), the taxonomic sampling here prioritizes including species with distinct body plans. However, rich taxonomic sampling of phenotypically similar taxa is important for accurately calculating rates of morphological evolution. Sampling only phenotypically distinct species will inflate the evolutionary rates for that clade.

c) Is the molecular dataset analyzed in this study a good representation of overall genetic evolution? There is insufficient description on the molecular data used in the study. What were the genes sampled? Are these non-coding genes?

d) The morphological data only includes synapomorphies, which will inflate the rates of phenotypic evolution. The manuscript states that "Having no remaining autapomorphies in the data set is another way of having a uniform distribution of autapomorphies" (Ln 493–494). That autapomorphies and invariant morphological characters are uniformly distributed across such a broad taxonomic scale is a major (and in my opinion, unreasonable) assumption.

e) The manuscript seems to advocate the use of Bayesian analysis due to its ability to account for

uncertainty at multiple levels of analysis. Howevever, the uncertainties are all reduced to point values when analyzing rates and disparity. This counters the strength of Bayesian analyses! For example, in the Methods section, the authors state that "estimates of divergence times and evolutionary rates can be averaged across the posterior sample of trees so that estimates take phylogenetic uncertainty into consideration." But by averaging, the authors are doing just the opposite.

f) Related to (e), the comparisons of rates and disparity are based on mean/mode of values. In fact, the manuscript reports on differences in disparity and evolutionary rates across geologic time and taxa, but Fig. 2 and 3 show that when the 95% confidence intervals are considered, there may not have been any significant changes to rates nor disparity throughout the course of diapsid and lepidosaur evolution.

g) The Methods section mentions that the phylogenetic tree with the highest posterior probability in order to remove ambiguity (Ln 328–332). Why weren't all trees after burn-in used to account for uncertainty in phylogenetic reconstruction?

h) The Methods section states that BEAST2 did not reach stationarity, but it is not mentioned in the text whether MrBayes analyses reached stationarity. How was stationarity in these results observed and ensured?

i) Whether Mkv model is able to model morphological evolution accurately for the taxonomic scope of the study should to be addressed. I understand that there isn't an alternative to Mkv model for performing phylogenetic analyses on morphological data under a Bayesian framework. Lack of existing methods, however, should not preclude justifying that Mkv is a reliable and robust model for the dataset.

j) By using total evidence approach under the decoupled phenotypic-molecular rates, is there a risk of enhancing phenotypic and genotic differences simply because that is the model used in the phylogenetic analysis? In my mind, this approach seems to be circular. If different models are used (although they are not the most likely model), does it still yield consistent results?

k) Certain methodological decisions are made because they improve "precision" (Ln 99, 447). Results could be precise, but inaccurate. How do we know that the results presented here are also accurate?

l) What is considered a "major" change in bauplan that are used as branches on phylogenetic trees where phenotypic and molecular rates are compared?

m) It was not clear to me whether polymorphisms were converted into NA scores for the total-evidence phylogenetic reconstruction. Based on my knowledge, polymorphic character coding should have been retained, but converted for disparity analysis.

n) "This number of characters is more than sufficient to provide reasonable estimates of disparity" (Ln 498–499). This statement is given without any justification. The paper that is referenced focuses on which type of morphometric measurements yield more robust results against bootstrap resampling, and is not directly applicable to the dataset used in this study.

o) Authors claim that the dataset being analyzed is the "largest." However, bigger does not mean better. How is the dataset used here better than other datasets?

2. Although "novelty" is subjective, I personally did not find this study to provide "novel" approaches or insights to evolutionary biology. In my opinion, the datasets, results, and conclusions presented do not provide sufficiently fresh insights. The authors claim that the "study is one of the very few assessing such megaevolutionary dynamics over broad taxonomic and chronological scales" but there

are certainly far more than "very few" published studies in the literature. In addition, the authors also claim that the study analyzed the "largest available data set on diapsid reptiles" (Ln 19). It is not clear to me which criterion this statement is based on. It is certainly not based on taxonomic sampling nor number of characters. The text needs to clarify what makes the dataset "largest."

For the study to be truly "novel," it would address questions such as: what type of genes undergo the fastest evolution in diapsids/lepidosaurs? Which morphological characters are evolving the fastest? Interrogating such topics would allow one to conclude how and why molecular and morphological evolution are "decoupled," which would bring a more nuanced discussion that the scope and aims of this study warrants. At the moment, the study lacks analyses that would lead to fresh insights. For instance, there is a discussion on transposable elements and microsats in snakes (Ln 283–301), but I believe that the genetic data used in this study did not include these elements. As such, the experimental design of the study does not allow one to address why morphological and molecular data sampled in this study are decoupled.

3. The hypotheses (listed in Ln 75–79) are not tested with statistical rigor, especially when comparing disparity and evolutionary rates across geologic time and taxa. Examples include:

a) "[...] phenotypic rates of evolution in early diapsids are consistently accelerated (above one and well above modal values) during most of the Permian, indicating elevated rates of evolution at the origin of major lineages of diapsid reptiles" (Ln 131–133). This is not statistically tested. The rates of evolution during the Permian seem to be very similar to the Triassic (Fig. 2a) or at least difficult to tell how different they are.

b) "This is coupled with relatively high rates of phenotyphic disparity" (Ln 134). I feel that this is not supported by what is shown visually in Fig. 2b. I feel that an explicit statistical test is needed here.

c) "[P]hylogenetic branches with the highest phenotypic rates are frequently those within the early branches of newly evolving clades" (Ln 228–229). Could this be tested via regression analysis on branch lengths and divergence time of preceding node?

4. In my opinion, the text does not provide sufficient justification on why diapsid reptiles are ideal taxa for investigating this topic. How are the results and conclusions of this study generalizable to other metazoans?

MINOR COMMENTS

> Ln 25: could remove "that such events are characterized by".
> Ln 27: "transforming" should be "transform".
> Ln 47–49: This sentence is vague. I would make it more specific (i.e., what do these previous studies support exactly?)
> Ln 50–51: How could the same references (#1, #2) be used as seminal literature on adaptive radiation as well as examples of "recent challenges" to these definitions of adaptive radiation?
> Ln 75: "major deep time evolutionary patterns" seems a bit redundant. I suggest "What are the major patterns of macroevolutionary rates and phenotypic diversity?"
> Ln 76: evolutionary rates do not "expand." I would use a different verb.
> Ln 77–78: This is an incomplete sentence.
> Ln 122: What is the unit for "~2.0" value? Same comment for Ln 124, 132.
> Ln 131–133, "[...] phenotypic rates of evolution in early diapsids are consistently accelerated (above one and well above modal values) [...]." Fig. 2a shows that the evolutionary rates do not fluctuate much during the Permian and is not elevated compared to Triassic levels. It's unclear to me how the rates during the Permian could be considered "accelerated." Also, the text does not explain

what "above one" means.
> Ln 177: It is not immediately clear what is meant by "modal levels".
> Ln 277: Change to "most genomic changes".
> Ln 283–284: High phenotypic rates do not necessarily suggest distinct morphology. Taxa could evolve quickly among existing body plans.
> Ln 343: Remove underscore in "Pleurosaurus_goldfussi".
> Ln 375–370: "To ensure that each independent single chain run in BEAST2 reached stationarity, we increased length of each analysis to 200 million generations": Increasing the length of the analysis does not ensure stationarity. This statement is better placed few paragraphs down (Ln 382–287).
> Ln 375–376: "The vast majority of our calibrations were based on tip-dating [...]." Were there clades that did not incorporate calibration via tip-dating approaches?
> Ln 476–478: "To reduce the negative impact of missing data, we removed all characters with more than 30% of missing data from the dataset, which resulted in a total of 19% missing data on the final data set--safely below the threshold of 25%". How do the authors know that 19% missing data is "safely" below the threshold? What is the cutoff between "safe" and "unsafe" levels of missing values?
> Ln 488: Change "bias distance matrices" to "biases in distance matrices".
> Ln 520–521: "only post-ordination methods can be used to produce a morphospace projection." I am unclear on what this statement is trying to say. First, ordination methods are not necessary for morphospace to exist (e.g., one can plot two trait values). Second, stating that projection of data requires a method for projecting data on new axes sounds tautalogical to me.
> Ln 521–523, "post-ordination methods have been more widely used in the literature making our results more directly comparable to previous studies." I do not believe this is true both theoretically and practially speaking. PC scores are notorious for its inability to be comparable across different sampling schemes.
> Ln 528–530: "Nonmetric multidimensional scaling was not used to preserve the metric properties of the dissimilarity matrix." But how is this helpful when the dissimilarity matrix is based on non-meristic phylogenetic characters?
> Fig. 1c: What are the outlier points? I would like to see mentions of these points in the text.
> Fig. 2–4: I recommend using a larger color spectrum to denote rate values on the phylogeny. It's very difficult to see which branches have elevated vs. reduced rates.

Reviewer #2 (Remarks to the Author):

This is an intriguing and worthwhile paper, but I have a few concerns the authors could address.

The main comment relates to the framing of "adaptive radiation" as the central metaphor. The authors do not make it clear what their concept of AR is, or how they test it relative to other evolutionary models. Despite this, they make reference in the Discussion to periods where the data "fit" AR vs fitting "other" evolutionary processes.

The concept of AR as an explanatory mechanism has been criticized heavily as reifying a collection of patterns based on an arbitrary threshold of continuous variation in evolutionary rates (Olson and Arroyo-Santos 2009; https://www.ncbi.nlm.nih.gov/pubmed/19921661).

Furthermore, the ecological theory of AR (Schluter 2000) includes explicit criteria including phenotype-environment correlation to conclude that AR has taken place.

How does this integrate with the timescales the authors are examining? We are looking here at tens of millions of years; at diversification patterns across entire ordinal lineages. How can that be linked mechanistically (in a philosophical sense) to the same patterns of AR via EO that are examined in the Losos/Stroud sense?

In an empirical sense, what phylogenetic or other models are being used to test the presence of AR

quantitatively? What are the contrasting models?

Ultimately, I think the data and results can stand alone without the theory-laden baggage of the AR metaphor. The authors are examining molecular/phenotypic rate disparities, particularly around major evolutionary events such as mass extinctions.

Specific comments:

There are a lot of outliers on the plots that seem to influence the overall conclusions; these need to be highlighted and discussed more. Figs. 1-3 all have massive outliers that seem to dominate the statistical results; can you assure us these are genuine?

Also, please use a different color scheme gradient other than red-purple-blue; it doesn't show up very well. Perhaps look at viridis, inferno, or magma.

Reviewer #3 (Remarks to the Author):

General comments

This paper by Simoes and collaborators explores the macroevolutionary patterns underlying the radiation of reptiles. The main goal of the paper was to explore if the emergence of major clades is congruent with patterns suggested by the adaptive radiation theory, such as increases in phenotypic rates and morphological disparity after mass extinctions. This was done basically by estimating the phenotypic rates and morphological disparity in diapsid reptiles. More specifically, using a total evidence approach and an updated phylogenetic dataset, the authors estimated morphological and molecular rates of evolution across reptiles and compared the morphological disparity among clades and across time. By describing major patterns of molecular and morphological evolutionary rates and morphological disparity variation, the authors found some of these to be consistent with patterns expected for adaptive radiations taking place after mass extinctions, an uncoupling between morphological and molecular rates and that clades with similar morphology are associated with different rates of morphological evolution.

Overall, I enjoyed the reading. Although some of the methods are not simple the authors explain most of it in such a way that make the paper easy to follow. However, while reading this manuscript I was constantly asking myself what was the central message of the paper. This is definitely not clear in its current form. I described some of my concerns below that if addressed might help to clarify what are the main findings of the paper and major implications for the field.

- The definition of what is an adaptive radiation needs to be clearer. This will be important to the interpretation of the rest of the paper. Some would argue that AR would involve fast diversifying lineages occupying distinct adaptive zones (including GG Simpson, see references below). This would also require to explore taxonomic diversification/speciation rates (see Cantalapiedra et al. 2017 Science which I was surprised was not cited by the authors).
- Since you have focused on one potential trigger of adaptive radiations (e.g. mass extinctions), how are you able to say that other increases in morphological rates (for example, snakes) do not conform AR? What about the evolution of key innovations or colonization of new areas? (see lines 200-203 or lines 303-305).
- The role of why to explore molecular rates of evolution here is not clear throughout the manuscript.

More specific comments

Evolutionary rates: please always specify the rates you are talking about. I also think the authors might want to explain a bit more how morphological/molecular rates were estimated.

I suggest the authors to include references regarding the different definitions of AR. The reader should be aware that the authors are choosing one of those definitions but that there are plenty of work on this matter even arguing that AR might not be a useful concept. See:

Givnish 2015 New Phytol 207
Losos & Mahler 2010 Chapter
Soulebeau et al 2015 Org Div Evol 15
Olson & Arroyo-Santos 2009 BioEssays 31
Glor 2010 Ann Rev Ecol Evol Syst

And references therein.

Lines 32-35 Mass extinctions represent only one among other “events” that could provide ecological opportunities

Line 44 and 45 Please define which rates are you referring here

Lines 57-64 I was not sure how these arguments above prevent adaptive radiations to be responsible for the origin of most biological diversity. Couldn’t constructive or episodic radiations still be “adaptive”? Maybe my concern here is related to the several different definitions of AR that should be clarified in the text.

Lines 76-78 Awkward wording

Lines 78-79 What are the expectations for radiations to conform the classic AR model?

Lines 79-82 I suggest the authors to state their main finding regarding their questions indicated in lines 75-79. Additionally, I do not see how their introduction links with their finding regarding phenotypic novelties with similar functions but different rates. And where rates of molecular evolution come into regarding the questions and what will be explored in the paper? What is the goal of investigating rates of molecular evolution here? More specifically, how rates of molecular evolution relate to the theory of adaptive radiations?

Figure 1a,b. I’m not sure what this figure represents. The authors should make clearer how they pooled these rates

Detailed point-by-point response to comments raised by referees

Author responses are in blue. In text citations are provided at the end of this document.

Referee #1 (Remarks to the Author):

Simoes and colleagues present a study investigating the rates of phenotypic and genetic evolution across diapsids. Using morphological characters based on a previously published phylogenetic dataset (138 taxa, 105 characters) and sequence data from GenBank, they tackle the challenging topic of assessing the presence of adaptive radiations in a phenotypically and taxonomically diverse group of diapsid reptiles. The authors propose that rates of morphological and genetic evolution are largely decoupled and elevated rates are not especially restricted to origins of new bodyplans. Additionally, they find convergent evolution of similar traits through different rates of evolution. The strengths of the study includes the incorporation of many fossils in the dataset, investigation into correlation between phenotypic and molecular rates of evolution, and use of two different software to assess convergence in results. The topics explored in the study is certainly of great interest to the field of evolutionary biology and the taxonomic range of the study is notable.

We thank the referee for their comments on the novelty and ambitious nature of our work.

It is important to clarify our taxonomic and character sampling: our phylogenetic analysis contains 347 morphological and 11,532 nuclear/mitochondrial characters, while our disparity analysis used a modified version of this dataset with fewer characters (105 characters) to comply with the methodology used (detailed in our Methods). Further, we would like to note that we did not find that "elevated rates are not especially restricted to origins of new bodyplans" as the referee suggests, but instead that elevated rates are associated with the origin of new body plans.

At the same time, I found the experimental design to be inadequate for addressing the main questions of the study. For example, it analyzes a dataset with very low taxonomic sampling with respect to the study's scope and objectives, makes a number of nested assumptions, does not fully take into account uncertainties, and lack statistical rigor when making claims. Consequently, the approaches used in this study are disjointed from the narrative scope and aims of the study. While I enjoyed reading the paper, I was ultimately not convinced of the robustness and reliability of the results and conclusions being presented. Please see below for my major comments on the paper, followed by more minor, specific comments.

Although we appreciate the referee's concerns, we strongly disagree with their overall assessment of our dataset and the conclusions that can be drawn. We detail our position further below.

GENERAL COMMENTS

1. The analysis contains many nested assumptions, most of which remain unverified for this dataset/group. While assumptions are unavoidable in any study, here is a compounding list of key assumptions that I have noted from data collection to analysis:

a) Taxonomic sampling contains 47 extant lepidosaur taxa for which both molecular and morphological data could be collected. This does not seem to be sufficient sampling for characterizing such a phenotypically diverse clade. More importantly, it is not clear to me how such a low taxonomic sampling could be used to reliably estimate taxonomic diversification which is used as a criterion for identifying adaptive radiations. Clear justifications for the taxonomic sampling must be provided.

It is important to clarify here that we did not "estimate taxonomic diversification" as the referee suggests. Our study was specifically designed to examine phenotypic and molecular rates of evolution as well as phenotypic disparity in deep geological time. We compared our data to periods of major taxonomic diversification from two recent and comprehensive studies assessing the taxonomic diversification in both early diapsid reptiles and lepidosaurs (Cleary *et al.* 2018; Close *et al.* 2019)—references 27 and 34 in the MS.

Original Line 213-216: *"…In contrast, both phenotypic and molecular evolutionary rates were stable and at low levels during the Cretaceous, the period where most of the first examples of modern lineages of squamates show up in the fossil record: the peak of Mesozoic taxonomic richness is reached at the end of the Cretaceous27,34".*

Concerning taxon sampling, in addition to having 47 extant taxa represented, we also have 33 species of fossil lepidosaurs in our dataset, totaling 80 lepidosaur taxa across all major living and extinct families. Both extant and fossil taxa are fundamental to retrieve more accurate diversification times and trees (Guillerme & Cooper 2016; Luo *et al.* 2020) and, therefore, evolutionary rates and other macroevolutionary patterns during the early stages of clade evolution.

It is also important to note that morphological datasets including fossils require observing key specimens spread out across the world in various institutions— there is no equivalent repository such as GenBank where all that data can be extracted from. Thus, it is incredibly time-consuming building morphological datasets and even more so when rare fossil samples are included. Our dataset includes the best-preserved fossil material from 51 institutions across 20 countries and currently represents the largest one available to explore broad-scale phylogenetic relationships among all major clades of diapsid reptiles. The few studies including all of those major diapsid reptile clades (living and extinct) have no more than 41 taxa (Chen *et al.* 2014), whereas our dataset has 138 taxa.

To assure the referee, our taxonomic sampling approach is consistent with several other important studies addressing evolutionary rates and/or diversification times across similar broad scales of time and taxonomic representation in evolutionary palaeobiology. For a few examples see the following in our attached reference list: Fischer *et al.* (2016); Brusatte *et al.* (2014); Close *et al.* (2015); Lee *et al.* (2013).

We now reinforce the strength and further details on our dataset composition in the Methods section (Lines 468-486).

b) Related to (a), the taxonomic sampling here prioritizes including species with distinct body plans. However, rich taxonomic sampling of phenotypically similar taxa is important for accurately

calculating rates of morphological evolution. Sampling only phenotypically distinct species will inflate the evolutionary rates for that clade.

To clarify, we did not claim to have prioritized the sampling of distinct body plans during dataset construction. In fact, we included all available fossil taxa that could provide insights on the early evolutionary stages of the clades sampled, irrespective of any preconceptions on their degree of morphological disparity. Therefore, the referee's statement on "sampling only phenotypically distinct species will inflate the evolutionary rates for that clade." is not applicable to our data set. Furthermore, the statement in itself is not supported by any reference from the referee, and, in fact, there is a study on bird evolution directly contradicting the referee's statement that morphologically disparate should match rates of evolution: Brusatte *et al.* (2014).

Our major goal was to focus taxonomic sampling on (i) having representatives from all major lineages of early diapsid reptiles and lepidosaurs (fossils); (ii) periods of time encompassing previously proposed periods of adaptive radiation; and (iii) taxa representing the earliest stages on the evolution of major lineages. Aspects (i) and (iii) are especially important for retrieving well supported phylogenetic hypotheses and accurate diversification times.

We have now made this aspect of our sampling protocol clearer in the revised MS –Lines 468-486.

c) Is the molecular dataset analyzed in this study a good representation of overall genetic evolution? There is insufficient description on the molecular data used in the study. What were the genes sampled? Are these non-coding genes?

The protein coding genes sampled and all loci accession numbers are available in our Supplementary Information: Supplementary Table S1. The molecular data were obtained from GenBank and provided in Supplementary Data 2. The procedure for molecular data collection, alignment, and analysis were also described in the Methods (original lines 351-387). Further, we discussed the nature of the fit of different gene types in our discussion and how they impact our conclusions (original lines 269-301).

Since the tree inferred using molecular data only (provided as Supplementary Fig. S1) has the exactly same structure concerning the relationships of the major lepidosaur families as a recently published genomic study (Streicher & Wiens 2017)—ref. 25 in the MS—we consider our phylogenetic signal to be consistent with the latest molecular hypotheses. We detailed this in our manuscript (original lines 111-113): *"Our initial non-clock Bayesian inference results with lepidosaurs indicate strong topological similarity between our molecular tree and a recent phylogenomic studies of lepidosaurs25*".

d) The morphological data only includes synapomorphies, which will inflate the rates of phenotypic evolution. The manuscript states that "Having no remaining autapomorphies in the dataset is another way of having a uniform distribution of autapomorphies" (Ln 493–494). That autapomorphies and invariant morphological characters are uniformly distributed across such a broad taxonomic scale is a major (and in my opinion, unreasonable) assumption.

Contrary to the referee's interpretation, we did sample autapomorphies for the phylogenetic analysis and subsequent rates. We made that statement in our methods: "*In the present dataset, there are some*

directly observed autapomorphies…" (original lines 491-492). The line quoted above by the referee is not a description of our dataset, but only a commentary on the behavior of measures of morphological disparity in the absence of autapomorphies. Autapomorphies were removed for the disparity analyses for reasons mentioned in our Methods. See Methods, subsection "Dataset adaptation for morphological disparity analyses".

We now make another mention of autapomorphies in the Methods (Line 473).

e) The manuscript seems to advocate the use of Bayesian analysis due to its ability to account for uncertainty at multiple levels of analysis. Howevever, the uncertainties are all reduced to point values when analyzing rates and disparity. This counters the strength of Bayesian analyses! For example, in the Methods section, the authors state that "estimates of divergence times and evolutionary rates can be averaged across the posterior sample of trees so that estimates take phylogenetic uncertainty into consideration." But by averaging, the authors are doing just the opposite.

The output of Bayesian inference analyses has tens of thousands of trees, each one with hundreds of nodes, each node containing its own set of parameters. Overall, this represents hundreds of thousands of data points in over 10 GB of data. It is unfeasible to illustrate and consider all of that data for the analyses in R. The alternative is filtering the data for a subsample or taking central tendency measures calculated by Mr. Bayes during the analysis, which does take into account all of those hundreds of thousands of sampled trees and is the method we employ here. One major advantage of central tendency measures calculated by the software is that it considers all trees sampled from the posterior distribution, which would not be the case if we performed further data filtering.

Furthermore, from a statistical point of view the referee is incorrect in stating that a central tendency measure does not reflect the variation available in the data. The median or mean of the parameters are influenced by all of the trees from the posterior, therefore being influenced by all of the data. In fact, mean values are influenced by data distribution.

Importantly, the only other available approach—implemented in R packages and previously taken by other similar studies, e.g. (Lloyd et al. 2012; Brusatte et al. 2014; Wang & Lloyd 2016)— would not consider data from various trees, but only provide single point estimates of those parameters based on a single tree topology (usually estimated from maximum parsimony).

Details of how data were extracted from Bayesian results were previously available in our original Supplementary Methods, but these have now been moved to the main Methods to prevent confusion (lines 637-657).

f) Related to (e), the comparisons of rates and disparity are based on mean/mode of values. In fact, the manuscript reports on differences in disparity and evolutionary rates across geologic time and taxa, but Fig. 2 and 3 show that when the 95% confidence intervals are considered, there may not have been any significant changes to rates nor disparity throughout the course of diapsid and lepidosaur evolution.

Statistical tests of significant changes in disparity across time bins were in our original Supplementary Information— previously Supplementary Fig. S18 (pairwise Mann-Whitney tests). Our results show a significant change in disparity across important time bins, such as in early diapsid evolution during the

Permian (Cisuralian-Guadalupian and Guadalupian-Lopingian), and in the Permian-Triassic, as well as early Triassic-middle Triassic transitions. In early lepidosaur evolution, statistical tests cannot detect a significant difference, which is why in our results and discussion we indicate no considerable gain in morphological disparity in early lepidosaur evolution until the late Cretaceous— one of the big reasons to reject the model of adaptive radiation in the early evolution of lepidosaurs.

We have now also provided additional statistical tests for both rates and disparity across time bins, the details of which are provided in the Methods. The results are provided as Supplementary Tables S3-12 and Supplementary Data S5.

g) The Methods section mentions that the phylogenetic tree with the highest posterior probability in order to remove ambiguity (Ln 328–332). Why weren't all trees after burn-in used to account for uncertainty in phylogenetic reconstruction?

Contrary to the referee's statement, we did not use the "phylogenetic tree with the highest posterior probability". We used the summary tree produced from the maximized product of the clades posterior probabilities. The line quoted by the referee was taken from a passage where we indicated the benefits of using summary trees from Bayesian inference, and the different ways available to obtain such summary trees.

Our summary trees were calculated with standard output tree procedures available in Mr. Bayes and BEAST2: the tree maximizing the product of the posterior probabilities taken from all the trees among the sampled posterior trees (as argued by the referee), which generates the maximum clade credibility tree (MCC) in BEAST2 and the maximum compatibility tree (MCT) in Mr. Bayes. Majority-rule consensus trees are also provided in the Supplementary Data if the reviewer or readers would like to check topological uncertainty.

We now make tree summary procedures clearer in the Methods (lines 653-657).

h) The Methods section states that BEAST2 did not reach stationarity, but it is not mentioned in the text whether MrBayes analyses reached stationarity. How was stationarity in these results observed and ensured?

Mr. Bayes reached stationarity and we now make that statement explicit in the Methods (line 565).

We would also like to note that all the typical diagnostic reports for Bayesian inference analysis, and cited in the Method section (ESS, ASDSF, PSRF, and log files), are available in our Supplementary Data, and were stated in our original Methods:

"Convergence of independent runs was assessed using: average standard deviation of split frequencies (ASDSF ~ 0.01), potential scale reduction factors [PSRF ≈ 1 for all parameters] and effective sample size (ESS) for each parameter was greater than 200 for Mr. Bayes. Independent BEAST runs were combined using LogCombiner v2.5.1 (available with the BEAST2 package) and checked for stationarity and convergence in Tracer v. 1.7.175. The ESS value of each parameter was greater than 200 and ASDSF < 0.01."

and

"...log files, prior parameters and posterior parameter values described in the results and figures can be found online at: https://dataverse.harvard.edu/privateurl.xhtml?token=9f850dff-0ab7-44fb-a183-7b0d01d04ae3." (Original lines 540-545).

i) Whether Mkv model is able to model morphological evolution accurately for the taxonomic scope of the study should to be addressed. I understand that there isn't an alternative to Mkv model for performing phylogenetic analyses on morphological data under a Bayesian framework. Lack of existing methods, however, should not preclude justifying that Mkv is a reliable and robust model for the dataset.

The Mkv model is only a small aspect of how morphological data is modeled (specifically, the Q matrix only). Several additional variables affecting evolutionary rates and divergence times are modeled by other prior parameters. For instance: variation on rates among characters (modeled by a gamma prior probability distribution); among lineage rate variation (modeled by the best fitting clock model); calibration times (given by the fossil ages and stratigraphic ranges); the inclusion of autapomorphic characters (performed here); among others.

All of those additional model specifications were already described in our original Methods (Original lines 363-466).

Finally, recent studies indicate that the mismatch between morphological data and the Mkv model of character evolution seems to be unproblematic in total evidence datasets, such as ours: (https://www.biorxiv.org/content/10.1101/679084v1).

j) By using total evidence approach under the decoupled phenotypic-molecular rates, is there a risk of enhancing phenotypic and genotic differences simply because that is the model used in the phylogenetic analysis? In my mind, this approach seems to be circular. If different models are used (although they are not the most likely model), does it still yield consistent results?

There is no risk. Independent modeled parameters (such as evolutionary rates for each data partition) have no effect upon each other in relaxed uncorrelated clock models (used here, as detailed in Methods) (original lines 399-424). Rates inferred for the morphological and molecular partitions can be exactly the same, or quite different, depending on the data and the evolutionary models for that particular partition, but not other partitions. Such partitioning schemes with models appropriate for each partition are quite standard in phylogenetics, especially in molecular phylogenetics (Posada 2008; Lanfear *et al.* 2016).

k) Certain methodological decisions are made because they improve "precision" (Ln 99, 447). Results could be precise, but inaccurate. How do we know that the results presented here are also accurate?

This is an extremely broad and open question. With empirical data, in which the true answer (or true tree, in this case) is not known, it is virtually impossible to know how accurate any analysis is. However, as we have detailed above and made clearer in our Methods, we have taken several approaches that are known from the methodological literature to improve the accuracy of phylogenetic inference, and subsequently inferred divergence times and evolutionary rates. Check original lines 376-466. For just a couple of examples:

E.g.: "Accounting for "diversity" sampling impacts tree priors[20], affecting divergence time precision and accuracy [21,54]."

"The vast majority of our calibrations were based on tip-dating, which accounts for the uncertainty in the placement of fossil taxa and avoids the issue of constraining priors on taxon relationships when implementing bound estimates for node-based age calibrations[54]. The range of the stratigraphic occurrence of the fossils used for tip-dating here were used to inform the uniform prior distributions on the age of those same fossil tips (thus allowing for uncertainty on the age of the fossils)."

Importantly, as mentioned above in section "1a" on data sampling, our dataset followed a long series of guidelines to ensure its quality of construction: personal collection of data, avoiding logical and biological biases, etc. This is an approach rarely taken in morphological phylogenetics — those practices have been discussed at length in our previous publication on this topic that had been referenced in our methods section (Simões *et al.* 2017).

For clarity, we summarized further aspects of our approach above for dataset construction in Methods (Lines 472-486).

l) What is considered a "major" change in bauplan that are used as branches on phylogenetic trees where phenotypic and molecular rates are compared?

To clarify, we never made reference to "major change in body plan" (or bauplan) in our MS. We make reference to the origin of major clades and the origin and evolution of new body plans.

A body plan is a subjectively defined term in the biological literature, as it is often based on a collection of key "typical" features that make a particular clade unique and quite distinct from others. Given the subjective nature of defining body plans, we make reference to only a few convergently evolved body plans that hardly any morphologists would contest, such as the protective morphotype of placodonts and turtles or the snake-like (serpentiform) body plan of snakes and amphisbaenians.

m) It was not clear to me whether polymorphisms were converted into NA scores for the total-evidence phylogenetic reconstruction. Based on my knowledge, polymorphic character coding should have been retained, but converted for disparity analysis.

It is explicitly stated in the original MS that they were converted to NAs for the disparity analysis:

"Polymorphisms were converted into NA scores, following previous recommendations and based on the reasoning above 57,65" (original lines 486-487).

n) "This number of characters is more than sufficient to provide reasonable estimates of disparity" (Ln 498–499). This statement is given without any justification. The paper that is referenced focuses on which type of morphometric measurements yield more robust results against bootstrap resampling, and is not directly applicable to the dataset used in this study.

The referee is mistaken. The reference mentioned—Ciampaglio *et al.* (2001) [Ref. 62 in the MS]—does not assess morphometric characters. This study assesses the performance of discrete

morphological characters (as used in phylogenetic datasets), as stated in their methods, and also when they sample discrete morphological characters from Foote's 1999 dataset for their empirical analysis.

o) Authors claim that the dataset being analyzed is the "largest." However, bigger does not mean better. How is the dataset used here better than other datasets?

See answer to point 1a above. Also, in the Methods section, there is a reference to the protocol used for the morphological dataset construction (Simões *et al.* 2017), which outlines all the rigorous criteria used when assembling morphological dataset. As mentioned in the previous point (n), we outline our dataset guidelines in the Methods (lines 472-486).

2. Although "novelty" is subjective, I personally did not find this study to provide "novel" approaches or insights to evolutionary biology. In my opinion, the datasets, results, and conclusions presented do not provide sufficiently fresh insights. The authors claim that the "study is one of the very few assessing such megaevolutionary dynamics over broad taxonomic and chronological scales" but there are certainly far more than "very few" published studies in the literature. In addition, the authors also claim that the study analyzed the "largest available dataset on diapsid reptiles" (Ln 19). It is not clear to me which criterion this statement is based on. It is certainly not based on taxonomic sampling nor number of characters. The text needs to clarify what makes the dataset "largest."

The referee does not provide any citations for their claim that there are "certainly far more" studies like ours in their taxonomic and chronological breadth. Without proper reference, this is simply open conjecture without any support, making it impossible for us to rebut.

In our Introduction (original lines 53-64), we made reference to the few studies quantitatively assessing evolutionary rates and/or morphological disparity in deep geological time scales (covering hundreds of millions of years). As we note there, however, no study has previously attempted to gather information on all major clades of early diapsid reptiles and lepidosaurs. Even fewer have data collected across sufficiently broad scales of time that are necessary to answer the questions we seek to address.

We reiterate our novel approach and findings below:
(1) Despite adaptive radiations being invoked several times to explain evolutionary patterns in reptiles, we found only one instance in which that claim could be supported, after the PTME.

(2) Morphological or molecular evolutionary rates had never been previously explored for all major lineages of lepidosaurs across millions of years. Therefore, any observed results would be necessarily new. Importantly, our main results support a recently proposed evolutionary radiation model, which is an alternative to the classical adaptive radiation model as described by Simpson.

(3) We further found that lineages developing functionally similar solutions to common adaptive problems do that at quite distinct morphological rates of evolution.

(4) Unexpectedly, we also found important negative consequences to the utilization of the widely used software package BEAST2 to the estimate of divergence times using total evidence dating (TED),

which, by itself, will be of great importance to several researchers in systematics and evolution biology conducting divergence time estimates.

In terms of dataset size and composition, please see our response to points 1a,b,n,o above.

For the study to be truly "novel," it would address questions such as: what type of genes undergo the fastest evolution in diapsids/lepidosaurs? Which morphological characters are evolving the fastest? Interrogating such topics would allow one to conclude how and why molecular and morphological evolution are "decoupled," which would bring a more nuanced discussion that the scope and aims of this study warrants. At the moment, the study lacks analyses that would lead to fresh insights. For instance, there is a discussion on transposable elements and microsats in snakes (Ln 283–301), but I believe that the genetic data used in this study did not include these elements. As such, the experimental design of the study does not allow one to address why morphological and molecular data sampled in this study are decoupled.

First, we would like to vigorously disagree that our study is any less novel than the one the referee proposes. Second, what the referee suggests would require a very distinct kind of dataset than the one collected here: morphometric characters instead of discrete morphological characters; and both regulatory and protein coding genes instead of protein coding genes only. We cannot address this with our current dataset for the following reasons:

On the morphological side, phylogenetically inferred evolutionary rates do not provide rate values for individual characters. It only provides rate values for data partitions in the dataset (e.g., morphological vs molecular partitions). It is theoretically possible to subdivide the morphological data into more partitions (e.g., cranial vs postcranial characters), but this comes at the cost of overparameterization and several other issues associated with the low number of characters in morphological datasets compared to molecular datasets. Methods to address this are still required. The alternative option, and most commonly implemented by neontologists, is the utilization of a few specific morphometric characters (e.g., body length, limb length, etc.) to estimate the rate of evolution on those continuous traits a posteriori on a given phylogenetic tree. Two major problems arise here: i) such a posteriori procedures have caveats compared to the approach we take here— already discussed in detail in our Methods and ii) we do not have morphometric data available, which would require a considerable amount of time to be obtained from all fossil data.

On the molecular side, such regulatory genes are rarely sampled for molecular systematics, and therefore hardly available on online repositories (e.g., GenBank), requiring a long-term project to sequence genomic data from all of the extant species included in this dataset.

Most importantly, such specific comparison between morphological and molecular traits are not necessary to answer our primary questions on adaptive radiations that we put forward in our Introduction: see Original lines 75-79. Importantly, we do not consider such questions raised by the referee to be superior or “more novel” than the questions we asked and results we presented here (see response to previous comment).

As a proof of concept, novelty and relevance of the questions that we do address in our study, we refer to recently published studies addressing similar questions in other paleobiological systems: Erwin *et al.* (2011); Hopkins & Smith (2015); Close *et al.* (2015)

3. The hypotheses (listed in Ln 75–79) are not tested with statistical rigor, especially when comparing disparity and evolutionary rates across geologic time and taxa. Examples include:

a) "[...] phenotypic rates of evolution in early diapsids are consistently accelerated (above one and well above modal values) during most of the Permian, indicating elevated rates of evolution at the origin of major lineages of diapsid reptiles" (Ln 131–133). This is not statistically tested. The rates of evolution during the Permian seem to be very similar to the Triassic (Fig. 2a) or at least difficult to tell how different they are.

The referee did not include the reference cited in these lines that were quoted: *"phenotypic rates of evolution in early diapsids are consistently accelerated (above one[26] and well above modal values)….*". Reference 26 is the manual of Mr. Bayes which explicitly states that evolutionary rates above 1.0 estimated with this software are considered to be "accelerated", whereas values below 1.0 are indicative of decelerating rates.

The rates in the Early and Middle Triassic follow a continuously ascending trendline that reaches its peak in the Middle Triassic, before the start decreasing from the mid-Middle Triassic onwards (Fig. 2), reaching the highest mean and median values for rates of evolution than at any other timeframe (new Supplementary Table S3).

We now include further statistical tests, including evolutionary rates across geological time bins, as suggested. See Supplementary Tables S3-12. Importantly, all our raw data files were also made available as part of our Supplementary Data, which can be accessed by any reader to repeat and verify our results. Further discussions are included in the main text (lines 197-222).

b) "This is coupled with relatively high rates of phenotyphic disparity" (Ln 134). I feel that this is not supported by what is shown visually in Fig. 2b. I feel that an explicit statistical test is needed here.

Please see our statistical test of disparity across time bins provided in the original Supplementary Fig. S18: Pairwise Mann-Whitney tests of phenotypic disparity across time bins. The middle Triassic has the highest absolute values for both evolutionary rates and morphological disparity among early diapsids (as depicted in the graphs and available in our supplementary data) and are statistically significant.

c) "[P]hylogenetic branches with the highest phenotypic rates are frequently those within the early branches of newly evolving clades" (Ln 228–229). Could this be tested via regression analysis on branch lengths and divergence time of preceding node?

We now provide those regression analyses as Supplementary Figures S18-21 (morphological and molecular rates vs node ages and branch duration in millions of years).

To summarize our results, we don't see any well supported trends, although there is a slight tendency for older nodes (at the origin of the major diapsid clades) to have higher evolutionary rates (as we had previously noted in the main text).

4. In my opinion, the text does not provide sufficient justification on why diapsid reptiles are ideal taxa for investigating this topic. How are the results and conclusions of this study generalizable to other metazoans?

It is unclear to us why the results must be comparable or generalizable to other metazoans. However, our protocols can be applied to many other study systems in future work. Further, we never claimed that diapsid reptiles are the "ideal taxa for investigating this topic". The choice of a study group relates to several aspects of our research program. Our research program is centered on reptile evolution in deep time, and such questions have never been properly asked or tested among all major lineages of reptiles— this is our primary reason to investigate them. Considering reptiles have survived through more than 300 million years, surviving 3 of the largest mass extinctions on Earth's history, and compose a substantial part of modern vertebrate diversity (with 10,500 species of lepidosaurs alone), understanding major evolutionary patterns in reptile evolution is highly relevant.

Reviewer #2 (Remarks to the Author):

This is an intriguing and worthwhile paper, but I have a few concerns the authors could address.

We are encouraged that the referee found our work intriguing and worthwhile.

The main comment relates to the framing of "adaptive radiation" as the central metaphor. The authors do not make it clear what their concept of AR is, or how they test it relative to other evolutionary models. Despite this, they make reference in the Discussion to periods where the data "fit" AR vs fitting "other" evolutionary processes.

In our work, we are specifically testing the main predictions of the classical theory of adaptive radiation *sensu* Simpson (1953) (as referenced and indicated in Introduction, original lines 25-32)) and compare this to alternative evolutionary models. Although we outlined the predictions and cited Simpson in our original Introduction, it seems that we did not make this clear enough.

To ensure there is no confusion, we have reworked the Introduction and indicate Simpson's adaptive radiation model more explicitly (lines 26-37).

The concept of AR as an explanatory mechanism has been criticized heavily as reifying a collection of patterns based on an arbitrary threshold of continuous variation in evolutionary rates (Olson and Arroyo-Santos 2009; https://www.ncbi.nlm.nih.gov/pubmed/19921661).

We value the insights from the opinion piece by Olson and Arroyo-Santos (2009). In fact, we agree with their opinion that this term has been used loosely by some authors in the recent literature. However, as noted by Olson and Arroyo-Santos (2009), such conflicts in the definitions of adaptive

radiations emerged much after the work that essentially coined the modern definition and its corollaries (Simpson 1953). This is why in our manuscript we made a statement in the first paragraph that we follow Simpson's definition and, based on that definition, what parameters we should investigate to detect adaptive radiations: "*The classical theory of adaptive radiation predicts that such events are characterized by high rates of phenotypic evolution, in combination with an expansion in phenotypic disparity and taxonomic diversity*" (original lines 25-27).

We disagree, however, with Olson and Arroyo-Santos opinion that the term "adaptive radiation" should be avoided altogether. Most widespread terms in biology suffer from similar problems of widespread mischaracterization of definitions, such as species, or homology. Yet, those concepts are still central to evolutionary biology, and unifying concepts can be reached—e.g. De Queiroz (2007). Further, the utilization of the term adaptive radiation has continued uninterrupted in the evolutionary literature in the past decade, indicating that abandoning the term has not been accepted by most evolutionary biologists—which we follow here.

Furthermore, the ecological theory of AR (Schluter 2000) includes explicit criteria including phenotype-environment correlation to conclude that AR has taken place. How does this integrate with the timescales the authors are examining? We are looking here at tens of millions of years; at diversification patterns across entire ordinal lineages. How can that be linked mechanistically (in a philosophical sense) to the same patterns of AR via EO that are examined in the Losos/Stroud sense?

Most of the criteria and ideas from Schluter (2000) were not developed to test adaptive radiations (AR) at broad taxonomic and geological scales as explored here, or in most paleontological studies addressing similar questions [e.g.,see (Lloyd *et al.* 2012; Brusatte *et al.* 2014; Close *et al.* 2015)], and as originally envisioned by the creators of the classic model of adaptive radiation (Osborn 1902; Simpson 1953). They were developed to address adaptive radiations at ecological (recent) timescales. Such differences in timescales are fundamentally important and, for that reason, Osborne (1902) proposed a fundamental distinction between "general" (i.e. geological timescales) and "local" (i.e. ecological timescales) adaptive radiations. Slater & Friscia (2019) more recently proposed that such distinctions should be recognized more often in evolutionary biology. Therefore, although several advances have been made on understanding modern adaptive radiations at ecological scales using approaches proposed and reviewed by Schlutter and Stroud & Losos (2016), those are usually restricted to recent timescales only.

Nevertheless, the role of mass extinctions in creating ecological opportunity (EO) during adaptive radiations in geological timescales is one of the few aspects of "general AR" covered in recent reviews on the ecological model of adaptive radiation (Stroud & Losos 2016). In our study, the one instance that we have evidence to support an adaptive radiation in its classical "Simpsonian" sense (during the Triassic) follows a major mass extinction (Permian-Triassic mass extinction). However, the ecological opportunity provided to reptiles after the extinction event (due to the demise of many early synapsid lineages) has already been explored in several papers and books [e.g., (Benton 2003; Chen & Benton 2012; Erwin 2015)], and are therefore not explored in detail here.

Taking the referee's opinion into account, we now make a clearer mention of the role of ecological opportunity in our discussions when addressing the Triassic radiation of reptiles (although not in great detail for the reasons above). We also added potential reasons for some clades to not radiate using

some of the ideas on ecological opportunity proposed and discussed by Schlutter and Stroud & Losos (2016).

In an empirical sense, what phylogenetic or other models are being used to test the presence of AR quantitatively? What are the contrasting models?

As mentioned in the comments above and in our original Introduction, the classical "Simpsonian" adaptive radiation (AR) model has specific predictions: *"high rates of phenotypic evolution, in combination with an expansion in phenotypic disparity and taxonomic diversity*" (original lines 25-27). We tested previous hypotheses that the origin of major clades on the aftermath of mass extinctions should be characterized by adaptive radiations, and therefore, have the specific evolutionary patterns proposed by Simpson. We performed analyses on evolutionary rates, morphological disparity, and compared those with recent data on diversification rates. Our results support the hypothesis of adaptive radiation in one instance (Triassic reptiles) but refutes that as an explanatory factor at others time frames (origin of early diapsids and early squamate clades).

The contrasting models were previously identified in our original Introduction in paragraphs 2 and 3 and include: challenges to the Simpsonian model include such alternatives as (1) episodic radiations, (2) constructive radiations. Constructive radiation was named and discussed in the Discussion, and referenced but not explicitly named in the Introduction.

To ensure there is no confusion about what alternative scenarios we explore, we have now reframed the Introduction to address episodic and constructive radiations (lines 63-70).

Ultimately, I think the data and results can stand alone without the theory-laden baggage of the AR metaphor. The authors are examining molecular/phenotypic rate disparities, particularly around major evolutionary events such as mass extinctions.

We appreciate the positive feedback on our data and results and we also appreciate the insightful comments on adaptive radiation concepts and definitions. However, we respectfully disagree that removing the discussion on adaptive radiations altogether from the manuscript would be of benefit to our study. The results gathered here have direct implications concerning previous hypotheses made by other paleontologists, specifically linking adaptive radiations to reptile evolution in the Triassic and the origin of the major diapsid and lepidosaur clades. Our results provide insights, validating one but refuting other hypotheses. Considering we used Simpson's classical definition of adaptive radiation and the high relevance of AR in the evolutionary and paleontological literature, not addressing AR when examining the origin of major lineages would leave us (justifiably) open to criticism by our peers.

Specific comments:

There are a lot of outliers on the plots that seem to influence the overall conclusions; these need to be highlighted and discussed more. Figs. 1-3 all have massive outliers that seem to dominate the statistical results; can you assure us these are genuine?

The largest outlier is represented by the evolutionary rates on the branch leading to snakes among lepidosaurs. We highlight this (lines 229-232), discussing how it may impact morphological phylogenies (lines 232-235) and provided potential explanations for the genomic basis for such drastic rates in snakes (lines 391-410). One of the other major outliers are represented by early sauropterygians, especially placodonts, during the early Triassic. We had mentioned that in the original text, but now we further detail its relevance in the revised manuscript (revised MS lines 336-349).

The detected outliers do not correspond to any unusual behavior of any of our analytical procedures and therefore we can affirm that they are "genuine". The fastest detected evolutionary rates make biological sense since they correspond to some of the most unique body plans observed among reptiles (armored, serpentiform and aquatic morphotypes), which seemed to evolve not too long after the divergence of those clades. Those changes include fast changes to both skull and postcranial morphologies, besides necessary changes in behavior and function, among all detected outliers.

Also, please use a different color scheme gradient other than red-purple-blue; it doesn't show Up very well. Perhaps look at viridis, inferno, or magma.

Response: We thank the referee for the suggestion. We tried multiple color schemes that are friendly to colorblindness, including the suggested ones. However, those specific color schemes have extreme positive values on the "yellow" side of the spectrum, making the observation of important outliers difficult to observe in a white background (see test below using "plasma"). The color scheme we utilize for branch rates (from dark blue to strong red), however, follows the same spectrum as observed using the "plasma" color scheme (= friendly to colorblindness), with the only difference that it stops at the red point of the spectrum, not going beyond into the hard-to-see yellow colors. Therefore, our color scheme is friendly to colorblindness as the "plasma" scheme, but without yellow tones. It further offers better contrast between branches for the lepidosaur tree (right).

We also note that the red to blue spectrum plotted on the tree diagrams is the standard for most studies trying to represent rates of trait evolution that we are aware of: e.g., https://onlinelibrary.wiley.com/doi/epdf/10.1111/pala.12432 . However, we are happy to modify the color scheme to other ones in case the editors or copyeditors think it will make for better contrast.

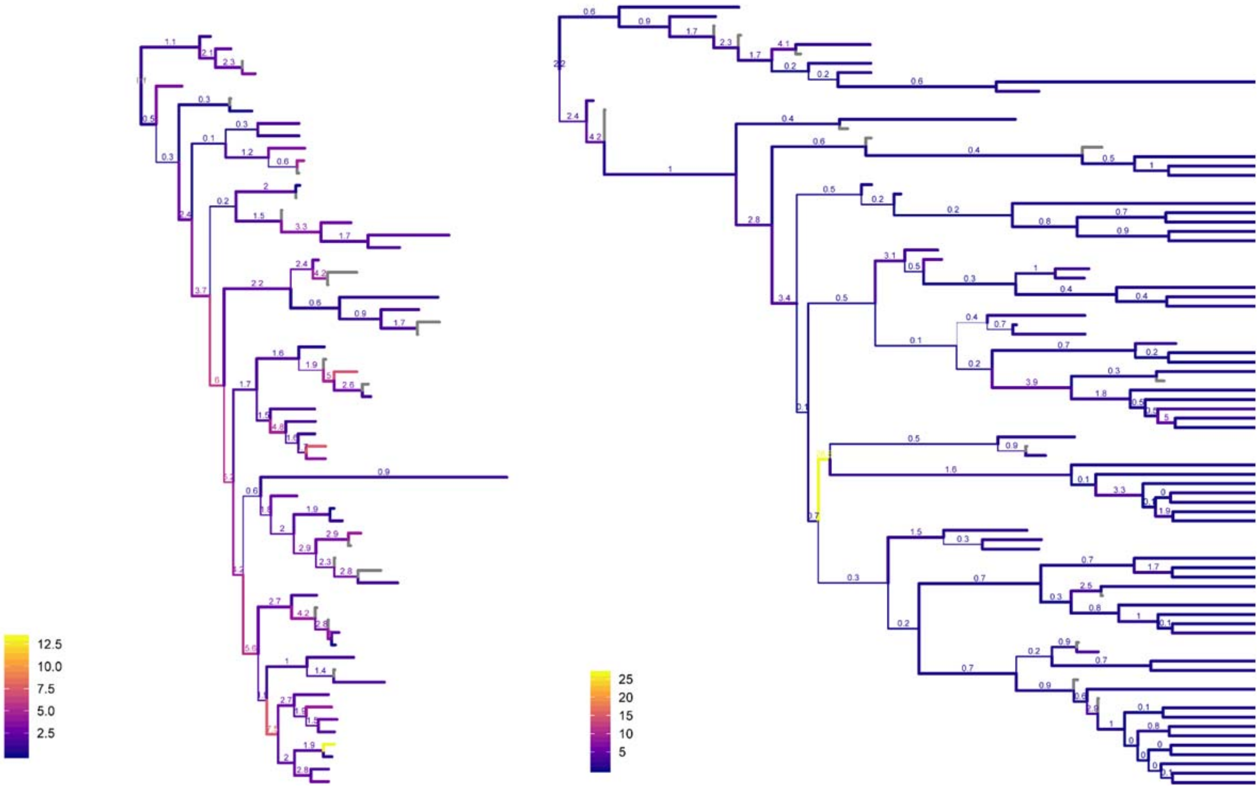
12.5
10.0
7.5
5.0
2.5
25
20
15
10
5

Reviewer #3 (Remarks to the Author):

General comments

This paper by Simoes and collaborators explores the macroevolutionary patterns underlying the radiation of reptiles. The main goal of the paper was to explore if the emergence of major clades is congruent with patterns suggested by the adaptive radiation theory, such as increases in phenotypic rates and morphological disparity after mass extinctions. This was done basically by estimating the phenotypic rates and morphological disparity in diapsid reptiles. More specifically, using a total evidence approach and an updated phylogenetic dataset, the authors estimated morphological and molecular rates of evolution across reptiles and compared the morphological disparity among clades and across time. By describing major patterns of molecular and morphological evolutionary rates and morphological disparity variation, the authors found some of these to be consistent with patterns expected for adaptive radiations taking place after mass extinctions, an uncoupling between morphological and molecular rates and that clades with similar morphology are associated with different rates of morphological evolution.

Thank you for providing this very accurate representation of the major goals and results of our study.

Overall, I enjoyed the reading. Although some of the methods are not simple the authors explain most of it in such a way that make the paper easy to follow. However, while reading this manuscript I was constantly asking myself what was the central message of the paper. This is definitely not clear in its current form. I described some of my concerns below that if addressed might help to clarify what are the main findings of the paper and major implications for the field.

We thank the referee for their comments and commending our manuscript. We are also happy that our methods were well explained. In our revised manuscript, we have focused our efforts on making sure the central message of the study is clearer, answering to your comments below.

- The definition of what is an adaptive radiation needs to be clearer. This will be important to the interpretation of the rest of the paper. Some would argue that AR would involve fast diversifying lineages occupying distinct adaptive zones (including GG Simpson, see references below). This would also require to explore taxonomic diversification/speciation rates (see Cantalapiedra et al. 2017 Science which I was surprised was not cited by the authors).

This comment about defining the term adaptive radiation is similar to the comment above by referee 2. As explained to referee 2, we tested the predictions of the classical theory of adaptive radiation *sensu* Simpson (1953) and have now made this clearer in the Introduction (see full reply to referee 2 above, and edited Introduction for specific changes: lines 26-37). Please note that we do mention fast diversification rates and new adaptive zones in the definition.

We have now added the reference to Cantalapiedra et al. 2017.

- Since you have focused on one potential trigger of adaptive radiations (e.g. mass extinctions), how are you able to say that other increases in morphological rates (for example, snakes) do not conform AR?

As mentioned in the comments above to referee 2, we follow Simpson's adaptive radiation (AR) model, which predicts: *"high rates of phenotypic evolution, in combination with an expansion in phenotypic disparity and taxonomic diversity*" (original lines 25-27). Thus, ARs are not defined exclusively by the occurrence of mass extinctions. The early evolution of snakes does not include all three of the predictions by the Simpsonian model, only increase in evolutionary rates. Therefore, it does not constitute AR.

What about the evolution of key innovations or colonization of new areas? (see lines 200-203 or lines 303-305).

Concerning the detected adaptive radiation in the Triassic (original lines 200-203), the long proposed explanatory cause for adaptive radiations following mass extinctions has been the occupation of vacant niches by surviving lineages (Simpson 1953). This is still the best explanation available, as indicated by recent reviews on the topic (Stroud & Losos 2016). This explanation has also been previously invoked for the reptile radiation in the Triassic [e.g., (Benton 2003; Chen & Benton 2012; Erwin 2015)], which we concur.

Now we have made this potential explanatory cause more explicit in our Discussion (revised MS lines 273-280; and 288-296).

Concerning the apparent limiting role of adaptive radiations on the deployment of most new body plans in diapsid reptile evolutionary history (original lines 303-305): this is evidenced by the fact that no other timeframe in the early evolution of diapsid reptiles matches all the three criteria outlined above. Therefore, they can be recognized as adaptive radiations *sensu* Simpson (see our points above on those criteria and definition). Indeed, it is considered that ecological opportunity provided by key innovations and colonization of new areas may trigger of adaptive radiations (Stroud & Losos 2016). However, even if those ecological factors were present during early diapsid evolution (especially the evolution of key innovations, as already discussed thoroughly in our Discussion), they did not result in changes to all three parameters predicted by the Simpsonian model.

- The role of why to explore molecular rates of evolution here is not clear throughout the manuscript.

There has been a decades long debate on the molecular drivers of fast phenotypic innovation, characteristic of adaptive radiations following Simpson's definition. Such molecular/genomic basis has been debated by many: Mayr to Gould to Carroll—thoroughly discussed in Mayr (1954); Gould (2002); etc. One possible way to explore that relationship is through the analysis of evolutionary rates on both datatypes, to understand if changes at the molecular level are coupled with changes at the phenotypic level. Only a handful of studies have utilized morphological and molecular data to address relationships between genome and phenotype in deep geological time, and so, we explored that potential correlation here for lepidosaurs (the only clade with a large enough representation of extant taxa providing both datatypes). As with nearly all other studies with combined morphological and molecular datasets, we have utilized nuclear and mitochondrial genes in our molecular sample. We

provide one of the few direct evidences indicating a strong mismatch on evolutionary rates between phenotype and nuclear/mitochondrial loci, which is extremely relevant to help elucidating the matters above. This is why we provided a potential explanation for that mismatch based on recent theories on genomic evolution on the second last paragraph of our Discussion (original lines 269-301).

We thank the referee for raising awareness over this, and have now added a brief statement on this topic in the Introduction as well (lines 74-89) for clarity on the importance of assessing molecular evolutionary rates.

More specific comments

Evolutionary rates: please always specify the rates you are talking about. I also think the authors might want to explain a bit more how morphological/molecular rates were estimated.

Rates are now specified in figure captions, and explained in further detail in Methods (lines 637-652)

To clarify, we already devote considerable text in the Methods to explaining the procedures used for clock rate estimation, divergence times, and tree typology. Evolutionary rates (morphological and molecular) are jointly estimated with the evolutionary tree (see first paragraph of Methods-lines 424-446), by utilizing molecular and morphological clocks in MrBayes and BEAST2. We also define how our estimates of evolutionary rates differ from other approaches commonly applied through phylogenetic comparative methods (lines 424-446). Our methods currently cover some of the basic foundations on clock analysis, but mostly explain parameters that tend to vary across different studies, such as our decisions for priors and advances on most current implementation, which are of great interest for the specialist reader (Method section entitled "Divergence time estimates and evolutionary rates", lines 590-657). Covering more on the foundations of clock analysis would be redundant with most of the well-established literature (Lemey *et al.* 2009; Yang & Rannala 2012; Ho & Duchêne 2014; Yang 2014). These references are now included in the first paragraph of our Methods (lines 433-434) section if readers want to explore the literature further.

I suggest the authors to include references regarding the different definitions of AR. The reader should be aware that the authors are choosing one of those definitions but that there are plenty of work on this matter even arguing that AR might not be a useful concept. See:

Givnish2015NewPhytol207
Losos&Mahler2010Chapter
Soulebeauetal 2015 Org Div Evol 15
Olson & Arroyo-Santos 2009 BioEssays 31
Glor 2010 Ann Rev Ecol Evol Syst

And references therein.

Following similar suggestion by referee 2, we have made it explicit what definition of adaptive radiation we use in the MS (first paragraph of Introduction). Now we also mentioned the criticism from Olson & Arroyo-Santos 2009, as well as two review papers from by (Losos 2010; Stroud & Losos 2016). We hope it is now clear in the Introduction which definition we follow (Simpson's—as

mentioned above) and acknowledge some of the literature on it. However, we consider that a more extensive discussion or debate on concepts and definitions of AR to be specific to specialist readers, and better addressed in specific evolutionary journals or review oriented papers.

Lines 32-35 Mass extinctions represent only one among other "events" that could provide ecological opportunities

Sentence edited.

Line 44 and 45 Please define which rates are you referring here

Sentence edited.

Lines 57-64 I was not sure how these arguments above prevent adaptive radiations to be responsible for the origin of most biological diversity. Couldn't constructive or episodic radiations still be "adaptive"? Maybe my concern here is related to the several different definitions of AR that should be clarified in the text.

We have now further clarified the Simpsonian definition of AR that we follow, along with the alternative models of constructive and episodic radiations in the Introduction (lines 26-37; 63-73).

As demonstrated by Hopkins & Smith (2015), what sometimes may be perceived as the outcome of a single adaptive radiation event (e.g. a highly diverse and heterogenic clade), implying an early burst in evolutionary rates, may actually be the outcome of a series of smaller episodic events—thus refuting the early burst model associated with Simpson's definition of adaptive radiations (Harmon *et al.* 2010).

Lines 76-78 Awkward wording

Sentence edited.

Lines 78-79 What are the expectations for radiations to conform the classic AR model?

As mentioned in the comments above, we follow Simpson's adaptive radiation (AR) model, which predicts: *"high rates of phenotypic evolution, in combination with an expansion in phenotypic disparity and taxonomic diversity*" (original lines 25-27) [first paragraph of Introduction]

Lines 79-82 I suggest the authors to state their main finding regarding their questions indicated in lines 75-79.

We now include more of our main findings right after the questions presented.

Additionally, I do not see how their introduction links with their finding regarding phenotypic novelties with similar functions but different rates. And where rates of molecular evolution come into regarding the questions and what will be explored in the paper? What is the goal of investigating rates

of molecular evolution here? More specifically, how rates of molecular evolution relate to the theory of adaptive radiations?

To the first point, before conducting our analyses we were not specifically predicting convergent functional morphotypes to evolve at different rates. These results were unexpected and we believe they merit discussion.

For the role of investigating molecular rates to understand adaptive radiations, see previous comments above.

Figure 1a,b. I'm not sure what this figure represents. The authors should make clearer how they pooled these rates.

Rates are now specified in figure captions.

REFERENCES

Benton, M. J. 2003. *When life nearly died: the greatest mass extinction of all time*. Thames & Hudson.

Brusatte, S. L., Lloyd, G. T., Wang, S. C. & Norell, M. A. 2014. Gradual Assembly of Avian Body Plan Culminated in Rapid Rates of Evolution across the Dinosaur-Bird Transition. *Current Biology*, **24**(20), 2386-2392.

Chen, X.-h., Motani, R., Cheng, L., Jiang, D.-Y. & Rieppel, O. 2014. The Enigmatic Marine Reptile *Nanchangosaurus* from the Lower Triassic of Hubei, China and the Phylogenetic Affinities of Hupehsuchia. *PLoS ONE*, **9**(7), e102361.

Chen, Z.-Q. & Benton, M. J. 2012. The timing and pattern of biotic recovery following the end-Permian mass extinction. *Nature Geosciences*, **5**(6), 375-383.

Ciampaglio, C. N., Kemp, M. & McShea, D. W. 2001. Detecting changes in morphospace occupation patterns in the fossil record: characterization and analysis of measures of disparity. *Paleobiology*, **27**(4), 695-715.

Cleary, T. J., Benson, R. B., Evans, S. E. & Barrett, P. M. 2018. Lepidosaurian diversity in the Mesozoic–Palaeogene: the potential roles of sampling biases and environmental drivers. *Royal Society Open Science*, **5**(3), 171830.

Close, R. A., Benson, R. B. J., Alroy, J., Behrensmeyer, A. K., Benito, J., Carrano, M. T., Cleary, T. J., Dunne, E. M., Mannion, P. D., Uhen, M. D. & Butler, R. J. 2019. Diversity dynamics of Phanerozoic terrestrial tetrapods at the local-community scale. *Nature Ecology & Evolution*.

Close, R. A., Friedman, M., Lloyd, G. T. & Benson, R. B. 2015. Evidence for a mid-Jurassic adaptive radiation in mammals. *Current Biology*, **25**(16), 2137-2142.

De Queiroz, K. 2007. Species concepts and species delimitation. *Systematic Biology*, **56**(6), 879-886.

Erwin, D. H. 2015. *Extinction–How Life on Earth Nearly Ended 250 Million Years Ago. Updated Edition*. Princeton University Press, Princeton.

Erwin, D. H., Laflamme, M., Tweedt, S. M., Sperling, E. A., Pisani, D. & Peterson, K. J. 2011. The Cambrian Conundrum: Early Divergence and Later Ecological Success in the Early History of Animals. *Science*, **334**(6059), 1091-1097.

Fischer, V., Bardet, N., Benson, R. B. J., Arkhangelsky, M. S. & Friedman, M. 2016. Extinction of fish-shaped marine reptiles associated with reduced evolutionary rates and global environmental volatility. *Nature Communications*, **7**(1), 10825.

Gould, S. J. 2002. *The Structure of Evolutionary Theory*. Harvard University Press, Cambridge, MA & London, 1433 pp.

Guillerme, T. & Cooper, N. 2016. Effects of missing data on topological inference using a Total Evidence approach. *Molecular Phylogenetics and Evolution*, **94, Part A**, 146-158.

Harmon, L. J., Losos, J. B., Jonathan Davies, T., Gillespie, R. G., Gittleman, J. L., Bryan Jennings, W., Kozak, K. H., McPeek, M. A., Moreno-Roark, F., Near, T. J., Purvis, A., Ricklefs, R. E., Schluter, D., Schulte Ii, J. A., Seehausen, O., Sidlauskas, B. L., Torres-Carvajal, O., Weir, J. T. & Mooers, A. Ø. 2010. Early bursts of body size and shape evolution are rare in comparative data. *Evolution*, **64**(8), 2385-2396.

Ho, S. Y. & Duchêne, S. 2014. Molecular□clock methods for estimating evolutionary rates and timescales. *Molecular Ecology*, **23**(24), 5947-5965.

Hopkins, M. J. & Smith, A. B. 2015. Dynamic evolutionary change in post-Paleozoic echinoids and the importance of scale when interpreting changes in rates of evolution. *Proceedings of the National Academy of Sciences*, **112**(12), 3758-3763.

Lanfear, R., Frandsen, P. B., Wright, A. M., Senfeld, T. & Calcott, B. 2016. PartitionFinder 2: New Methods for Selecting Partitioned Models of Evolution for Molecular and Morphological Phylogenetic Analyses. *Molecular Biology and Evolution*.

Lee, M. S. Y., Soubrier, J. & Edgecombe, G. D. 2013. Rates of phenotypic and genomic evolution during the Cambrian explosion. *Current Biology*, **23**(19), 1889-1895.

Lemey, P., Salemi, M. & Vandamme, A.-M. 2009. *The phylogenetic handbook: a practical approach to DNA and protein phylogeny*. Cambridge University Press, Cambridge.

Lloyd, G. T., Wang, S. C. & Brusatte, S. L. 2012. Identifying heterogeneity in rates of morphological evolution: Discrete character change in the evolution of lungfish (Sarcopterygii; Dipnoi). *Evolution*, **66**(2), 330-348.

Losos, J. B. 2010. Adaptive Radiation, Ecological Opportunity, and Evolutionary Determinism. *The American Naturalist*, **175**(6), 623-639.

Luo, A., Duchêne, D. A., Zhang, C., Zhu, C.-D. & Ho, S. Y. W. 2020. A Simulation-Based Evaluation of Tip-Dating Under the Fossilized Birth–Death Process. *Systematic Biology*, **69**(2), 325–344.

Mayr, E. 1954. Change of genetic environment and evolution.

Osborn, H. F. 1902. The law of adaptive radiation. *The American Naturalist*, **36**(425), 353-363.

Posada, D. 2008. jModelTest: phylogenetic model averaging. *Molecular Biology and Evolution*, **25**(7), 1253-1256.

Schluter, D. 2000. Ecological Character Displacement in Adaptive Radiation. *The American Naturalist*, **156**(S4), S4-S16.

Simões, T. R., Caldwell, M. W., Palci, A. & Nydam, R. L. 2017. Giant taxon-character matrices: quality of character constructions remains critical regardless of size. *Cladistics*, **33**(2), 198-219.

Simpson, G. G. 1953. *The Major Features of Evolution*. Columbia University Press, New York, 434 pp.

Slater, G. J. & Friscia, A. R. 2019. Hierarchy in adaptive radiation: a case study using the Carnivora (Mammalia). *Evolution*, **73**(3), 524-539.

Streicher, J. W. & Wiens, J. J. 2017. Phylogenomic analyses of more than 4000 nuclear loci resolve the origin of snakes among lizard families. *Biology Letters*, **13**(9).

Stroud, J. T. & Losos, J. B. 2016. Ecological opportunity and adaptive radiation. *Annual Review of Ecology, Evolution, and Systematics*, **47**, 507-532.

Wang, M. & Lloyd, G. T. 2016. Rates of morphological evolution are heterogeneous in Early Cretaceous birds. *Proceedings of the Royal Society of London B: Biological Sciences*, **283**(1828).

Yang, Z. 2014. *Molecular evolution: a statistical approach*. Oxford University Press.

Yang, Z. & Rannala, B. 2012. Molecular phylogenetics: principles and practice. *Nature Reviews Genetics*, **13**(5), 303-314.

Reviewers' comments:

Reviewer #1 (Remarks to the Author):

I appreciate the authors taking the time to address my and other reviewers' comments. Overall, the revised manuscript is improved, and in my mind, much clearer and easier to follow. I'd also like to apologize for some of the misinterpretations and misrepresentations of the authors' text in my previous review. It was not my intention to do so--it was an honest mistake arising from my effort to understand the details of such a large and complex study.

While the manuscript has been improved, there are general items that I'd like the authors to still address:

1. I am still a bit puzzled by the observation that "When observed across time, phenotypic rates of evolution in early diapsids are consistently accelerated relative to the base of rates (above one32, and well above mean, median and modal values) during most of the Permian, indicating elevated rates of evolution at the origin of the major lineages of diapsid reptiles (Fig. 2a,c, Supplementary Table S3)" (Ln 194–197). This statement, I believe, implies that early diapsids show greater rates of phenotypic evolution in the Permian than in the Triassic. When I look at Fig. 2a, I do not see greater values for "phenotypic evolutionary rates" in Permian compared to the Triassic. When I refer to Table S3, I see that median phenotypic rates of early diapsids are 2.34, 1.495, 1.63, 1.7, 1.97 in Permian, compared to 1.7, 1.97 in Triassic. These values to not seem to be notably different. It's likely that I am misunderstanding this part of the study, but I'd like to think that I will not be the only one confused with this observation. I suggest that the authors ensure that these concepts and results are clearly explained.

2. In my original comments concerning the "novelty" of the study, I pointed to how the molecular data used in the analysis did not capture the nuance of genetic evolution required to untangle "how they [rates of phenotypic evolution and disparity] relate to rates of molecular evolution" (Ln 15–18). I still think that 16 genes are not adequately representative of "molecular evolution." In response to my original comment on gene sampling, the authors respond that "On the molecular side, such regulatory genes are rarely sampled for molecular systematics, and therefore hardly available on online repositories (e.g., GenBank), requiring a long-term project to sequence genomic data from all of the extant species included in this dataset." To me, this is an unsatisfactory answer and illustrates how the experimental design do not quite match the biological question being investigated (refer to my original comment for an approach needed to robustly address the relationship between genetic and phenotypic rates of evolution). From my perspective, the authors' conclusion that molecular and phenotypic rates are "decoupled" is hyperbolic based on the data analyzed here. This outcome may be simply due to a mismatch between what the genes code and the phenotype being characterized. It may still be an intriguing and worthwhile result, but I suggest that the authors explicitly mention this caveat in the manuscript.

3. The methods section (335 lines long) is nearly the length of the preceding sections (402 lines long). Because there will already be a supplementary file, I suggest moving some methodological details to SI.

4. A more stylistic rather than conceptual criticism, but the term "markedly" is used often in the manuscript but it is a vague word. It should be replaced by more precise words that describe what is meant by "markedly."

5. I found more than a few typos and minor errors in the manuscript. I point to ones that I found below, but there may be more that I missed. I advise that the authors give a thorough proofreading.

MINOR COMMENTS:

> Ln 37: I think the word "promptly" is vague and unnecessary in this sentence.
> Ln 92–95: "If traditionally used protein coding nuclear and mitochondrial markers are in fact not the primary drivers of phenotypic diversification, then phenotypic rates of evolution would be expected to be decoupled from rates at protein coding loci." This statement is partially true because protein coding genes could have variable effects on phenotype, generating low correlation between genetic and phenotypic changes. This statement should be revised to more accurately reflect the complexity of genotype-phenotype linkage.
> Ln 125: "of change" or "of evolution" should be inserted after "molecular rates".
> Ln 144: It's unclear to me what "well calibrated" means. Either remove the word or briefly explain what is meant by it.
> Ln 148: "the" is repeated.
> Ln 182: As I commented in my last review, I would like to know what these rate values mean. For example, is it average character state changes per million years? Or is it relative rates that have been corrected by some standard value?
> Ln 320: remove "of" after "other".
> Ln 324: It's unclear to me what "capacitating role" means.
> Ln 336–338: "Key innovations" are typically defined by subsequent radiation (e.g., Rabosky 2017 Phil Trans Royal Soc B), so I feel that stating that some "key innovations" do not lead to adaptive radiation seems inaccurate by definition. If a morphological feature does not lead to radiations, then the feature simply ceases to be a "key innovation".
> Ln 506: Change "representativeness" to "representation".
> Ln 553: Reference should be given for the Mkv model (Lewis 2001?).

Fig 2–4
> Key for how branch thickness denotes posterior probability should be added. Even with a key, I think it would still be difficult to tell the thickness apart, so perhaps just indicating PP > 0.90 with a single thickness may be a better way to illustrate posterior support. Also I was not certain of what the posterior probability is for? Since it's not on the internal nodes, I assume it's not node support. Is it referring to posterior support for rate values? This should be clarified in text and/or figure captions.
> I noted last time (as well as one other reviewer) that the color scheme for the branches in Fig. 2–4 are very difficult to discern. I understand that the colors should be colorblind-friendly, but there must be a set of three colors that would work (e.g., blue, orange, and one other color). I know that the color palette used here is the default color scheme. As I'm sure the authors are aware, default settings are often not the best settings to use and this is a great example! I respectfully urge that the authors use a more discernible color palette.

Fig 3, 4
> Fix the typo "Banch width".

SUPPLEMENTARY INFORMATION
> p. 1: Correct the typo "Supplementaty".

Reviewer #2 (Remarks to the Author):

The authors have responded to most of my concerns. I recommend it be accepted/

I will say that I don't really agree with their optimistic embrace of the Simpson model. This is a 70 year old, subjective, qualitative, verbal model that is applied imprecisely across time and phylogeny, and is adaptable to telling any story.

In this case, we don't really know much (or at least the authors don't present) a robust picture of the

ecological context, certainly not enough to discriminate between alternative sources of novel adaptive zones.

I think the current analysis will provide a good footprint for future investigations, but I think the framing is stuck to the past.

Reviewer #3 (Remarks to the Author):

This is an improved version of this contribution and I thank the authors for responding my concerns. However, I still have one major concern: to not explicitly take taxonomic diversification into account. One of the goals of this study was to explore “which periods in early diapsid and lepidosaur evolution can be characterized as adaptive radiations as predicted by the Simpsonian model”. The paper is mostly built by discussing the predictions of the Simpsonian model of adaptive radiations. However, I’m afraid the analyses performed are not sufficient to allow them to reach part of their conclusions. A rapidly increase in taxonomic diversification is part of the predictions of the Simpsonian model but the authors do not perform any formal test regarding this macroevolutionary axis. So, basically, they are exploring if - and only if - phenotypic rates and disparity plus rates of molecular evolution change through time and among clades. Without analyzing the pace of taxonomic diversification, it is not possible to say whether or not clades correspond to adaptive radiations, even when applying the definitions claimed by the authors. Because of that I’m afraid this might prevent the authors to fully understand whether or not the analyzed lineages correspond to adaptive radiations.

Referee #1 (Remarks to the Author):

I appreciate the authors taking the time to address my and other reviewers' comments. Overall, the revised manuscript is improved, and in my mind, much clearer and easier to follow. I'd also like to apologize for some of the misinterpretations and misrepresentations of the authors' text in my previous review. It was not my intention to do so--it was an honest mistake arising from my effort to understand the details of such a large and complex study.

Response: We appreciate the positive feedback from the referee. We agree that our study is large and complex, and that it takes time to grasp all of its intricacies and broader impact. We are happy to learn that the current version makes the entire message much clearer.

While the manuscript has been improved, there are general items that I'd like the authors to still address:

1. I am still a bit puzzled by the observation that "When observed across time, phenotypic rates of evolution in early diapsids are consistently accelerated relative to the base of rates (above one32, and well above mean, median and modal values) during most of the Permian, indicating elevated rates of evolution at the origin of the major lineages of diapsid reptiles (Fig. 2a,c, Supplementary Table S3)" (Ln 194–197). This statement, I believe, implies that early diapsids show greater rates of phenotypic evolution in the Permian than in the Triassic. When I look at Fig. 2a, I do not see greater values for "phenotypic evolutionary rates" in Permian compared to the Triassic. When I refer to Table S3, I see that median phenotypic rates of early diapsids are 2.34, 1.495, 1.63, 1.7, 1.97 in Permian, compared to 1.7, 1.97 in Triassic. These values to not seem to be notably different. It's likely that I am misunderstanding this part of the study, but I'd like to think that I will not be the only one confused with this observation. I suggest that the authors ensure that these concepts and results are clearly explained.

Response: As a minor correction to the referee's statement, median rates for early diapsids in the Permian are 2.34, 1.495, 1.63. The relative rate values 1.7, 1.97 are for the Early and Middle Triassic, respectively.

In the statement quoted above by the referee we mentioned rates of evolution in the Permian only. We do not make any reference to the Triassic. Therefore, we are not comparing the rates in the Permian to the ones in the Triassic in that statement. As we mention in the quoted statement rates during the Permian "*are consistently accelerated relative to the base of rates (above one32, and well above mean, median and modal values*)". Rates above one are indicative of accelerated rates relative to the base (background) rate of evolution, which we indicated in that statement between parentheses and with reference 32, and also in our Method (lines 635-642 in current MS). As we mention explicitly, those rates are accelerated relative to the background rate, and we do not mention the Triassic, and we hope this clarifies the confusion now.

2. In my original comments concerning the "novelty" of the study, I pointed to how the molecular data used in the analysis did not capture the nuance of genetic evolution required to untangle "how they [rates of phenotypic evolution and disparity] relate to rates of molecular evolution" (Ln 15–18).

I still think that 16 genes are not adequately representative of "molecular evolution." In response to my original comment on gene sampling, the authors respond that "On the molecular side, such regulatory genes are rarely sampled for molecular systematics, and therefore hardly available on online repositories (e.g., GenBank), requiring a long-term project to sequence genomic data from all of the extant species included in this dataset." To me, this is an unsatisfactory answer and illustrates how the experimental design do not quite match the biological question being investigated (refer to my original comment for an approach needed to robustly address the relationship between genetic and phenotypic rates of evolution). From my perspective, the authors' conclusion that molecular and phenotypic rates are "decoupled" is hyperbolic based on the data analyzed here. This outcome may be simply due to a mismatch between what the genes code and the phenotype being characterized. It may still be an intriguing and worthwhile result, but I suggest that the authors explicitly mention this caveat in the manuscript.

Response: As we indicated in our introduction and previous response, *" such specific comparison between morphological and molecular traits are not necessary to answer our primary questions on adaptive radiations that we put forward in our Introduction: see Original lines 75-79*". Our primary research questions from lines 75-79 (97-103 in the current MS) were: "What are the major deep time evolutionary patterns concerning evolutionary rates and phenotypic disparity? Do most periods of expansion of evolutionary rates and/or morphological disparity occur at the origin of major clades and new body plans? Which periods in early diapsid and lepidosaur evolution can be characterized as adaptive radiations as predicted by the Simpsonian model? Can we identify a correlation between periods of fast increase in morphological and molecular evolutionary rates in lepidosaurs?"

As evident from the above, and our previous response to this topic, addressing the nuances of molecular and morphological correlation was not part of our study from the beginning, and this is why our methodological design was not aimed to address this question. However, as we do have both morphological and molecular data available, we consider it would be unreasonable to ignore the observed decoupling of morphological and molecular rates from the data we have available. We choose to describe these patterns, rather than ignoring them, even if this was not part of our initial question.

We hope to have made this clearer now, by including the statement "*decoupling between both rates in the context of the data available herein*" where we show this particular result (lines 155-156), so there is no misunderstanding. We believe that our conclusion concerning this topic (lines 373-384) also properly indicate that investigating the genetic basis of phenotypic evolution is a worthwhile topic, although outside the scope of the present work and ability of the current data set, and modern genomic theory support the hypothesis that evolutionary rates on protein coding genes and morphological evolution are expected to be decoupled: e.g., "*Although outside the scope of the present study, we consider that the assessment of rates of evolution on conserved regulatory regions and a higher sampling of protein coding genes to be a fundamental next step on the investigation of the genomic basis for fast phenotypic change in reptiles*" (Lines 386-389)"

3. The methods section (335 lines long) is nearly the length of the preceding sections (402 lines long). Because there will already be a supplementary file, I suggest moving some methodological details to SI.
Response: Considering the Referee missed large sections of our supplementary methods during the first round of review, the Senior Editor asked us to move this information into the main Methods section:"*there are no word limits in the Methods section so you are free to explain your approach fully. Please note that we require that all methods are described so that they can be understood without the reader needing to refer to previous work." (Dr. Furlong).*

4. A more stylistic rather than conceptual criticism, but the term "markedly" is used often in the manuscript but it is a vague word. It should be replaced by more precise words that describe what is meant by "markedly."
Response: We recognize that removing redundancies is important. We now use "*extremely*" in line 357 and "significantly" in line 742, along with other substitutions of the word "markedly".

5. I found more than a few typos and minor errors in the manuscript. I point to ones that I found below, but there may be more that I missed. I advise that the authors give a thorough proofreading.
Response: We appreciate the effort on catching the typos. We have corrected those accordingly.

MINOR COMMENTS:

> Ln 37: I think the word "promptly" is vague and unnecessary in this sentence.
Response: word deleted.

> Ln 92–95: "If traditionally used protein coding nuclear and mitochondrial markers are in fact not the primary drivers of phenotypic diversification, then phenotypic rates of evolution would be expected to be decoupled from rates at protein coding loci." This statement is partially true because protein coding genes could have variable effects on phenotype, generating low correlation between genetic and phenotypic changes. This statement should be revised to more accurately reflect the complexity of genotype-phenotype linkage.
Response: It is true that the genotype-phenotype linkage is complex (e.g., various number of genes operating on a single anatomical feature) and this may have an influence on the level of correlation between rates of specific molecular sites and phenotypic characters; however, as we utilized the overall mean of molecular and phenotypic rate (not a one-to-one gene-morpho mapping), such intricacies would be expected to be irrelevant at this level of analysis. We expect that to be relevant it should be accounted for in targeted analysis for specific molecular side-anatomical feature comparisons.

> Ln 125: "of change" or "of evolution" should be inserted after "molecular rates".
Response: words added.

> Ln 144: It's unclear to me what "well calibrated" means. Either remove the word or briefly explain what is meant by it.

Response: word deleted.

> Ln 148: "the" is repeated.
Response: word deleted.

> Ln 182: As I commented in my last review, I would like to know what these rate values mean. For example, is it average character state changes per million years? Or is it relative rates that have been corrected by some standard value?
Response: This has already been addressed in the previous submission. See meaning for absolute and relative rate values in Methods (lines 635-642 in current MS) and main text figure captions for figure 1 (*"Rates are relative to the mean posterior estimate of base of the clock rate value (= 0.00345 substitutions per character per Myr"*).

> Ln 320: remove "of" after "other".
Response: word deleted.

> Ln 324: It's unclear to me what "capacitating role" means.
Response: it means that ecological opportunity capacitates (make something capable of a particular action: obtained from Oxford dictionary) adaptive radiation following mass extinctions.

> Ln 336–338: "Key innovations" are typically defined by subsequent radiation (e.g., Rabosky 2017 Phil Trans Royal Soc B), so I feel that stating that some "key innovations" do not lead to adaptive radiation seems inaccurate by definition. If a morphological feature does not lead to radiations, then the feature simply ceases to be a "key innovation".
Response: The term key innovations can be highly subjective. To avoid confusion, we specify what we mean by key innovation. "*defined here as the appearance or subsequent change of morphological structures inferred to be of high adaptive value to explore a new ecological niche*" (lines 283-285 in current MS).

> Ln 506: Change "representativeness" to "representation".
Response: word corrected.

> Ln 553: Reference should be given for the Mkv model (Lewis 2001?).
Response: reference added.

Fig 2–4
> Key for how branch thickness denotes posterior probability should be added. Even with a key, I think it would still be difficult to tell the thickness apart, so perhaps just indicating PP > 0.90 with a single thickness may be a better way to illustrate posterior support. Also I was not certain of what the posterior probability is for? Since it's not on the internal nodes, I assume it's not node support. Is it referring to posterior support for rate values? This should be clarified in text and/or figure captions.

Response: The branch width relative to posterior probabilities (PP) is only meant to give an overall idea of branch support for the figures in the main text, as implemented in a recent study for similar purposes (Paterson *et al.* 2019). The actual posterior probabilities are detailed in the supplementary

information figures, for those who wish to investigate clade support accordingly. We indicate in the figure caption that full details for the tree are provided in the Supplementary material.

Importantly, morphological data sets do not have a single standard value for posterior probabilities (e.g., 0.9, as suggested) that can act as a standard single threshold, as it is often used in molecular data sets. Variance in PP is much greater in morphological data, and tends to be higher depending on the proportion of fossil taxa sampled, since the more fossil taxa/missing data added to a data set the greater the reduction in tree support and resolution (Luo *et al.* 2020). Additionally, as one portion of our data set combines both morphological and molecular data, that tree sector will always have a much higher support than the rest of the trees which is based on morphological data only.

To clarify, posterior probabilities are always necessarily associated with clades. They can be plotted either on nodes or branches at the base of those clades, but the difference is merely esthetic, and both options are available in most tree visualizations software (e.g., FigTree). We have made this clear in the figure caption.

> I noted last time (as well as one other reviewer) that the color scheme for the branches in Fig. 2–4 are very difficult to discern. I understand that the colors should be colorblind-friendly, but there must be a set of three colors that would work (e.g., blue, orange, and one other color). I know that the color palette used here is the default color scheme. As I'm sure the authors are aware, default settings are often not the best settings to use and this is a great example! I respectfully urge that the authors use a more discernible color palette.

Response: The current color scheme is not a default option (since there is no default option in the GGTREE R package used to compose the illustration). As indicated in our previous response to this topic, we tried several options of colorblind friendly schemes. However, most palettes (see previous response with an example embedded) result in the high end of the spectrum having a yellow color, which is even harder to observe. The color scheme we utilize (from dark blue to strong red) is a modification of the “plasma” color scheme (= friendly to colorblindness), with the only difference that it stops at the red point of the spectrum, not going beyond into the hard-to-see yellow colors. Therefore, our color scheme is friendly to colorblindness as the “plasma” scheme, but without yellow tones. We are happy to modify the color scheme to other ones that editors or copyeditors may eventually suggest. We further note that the color scheme here is only for improving the quality of the figure and regardless of the colour scheme used, there are numerical values for the evolutionary rates indicated above branches, so even if this picture is printed in black/white, readers can still read the rates values from the tree.

Fig 3, 4
> Fix the typo "Banch width".
Response: word corrected.

SUPPLEMENTARY INFORMATION
> p. 1: Correct the typo "Supplementaty".
Response: word corrected.

Referee #2 (Remarks to the Author):

The authors have responded to most of my concerns. I recommend it be accepted/

I will say that I don't really agree with their optimistic embrace of the Simpson model. This is a 70 year old, subjective, qualitative, verbal model that is applied imprecisely across time and phylogeny, and is adaptable to telling any story.

In this case, we don't really know much (or at least the authors don't present) a robust picture of the ecological context, certainly not enough to discriminate between alternative sources of novel adaptive zones.

I think the current analysis will provide a good footprint for future investigations, but I think the framing is stuck to the past.

Response: We thank the Referee for recommending this manuscript to be accepted. However, we strongly disagree with some of the provided statements:

I) As a point of correction to the Referee's last remark: Simpson's work did not constitute a "subjective, qualitative, verbal model". In fact, Simpson's two classical books (Simpson 1944; 1953) that give birth to the modern concept of adaptive radiation illustrate the results of multiple quantitative studies assessing morphological change in the fossil record across various timescales, which can be easily checked: chapters II and III of the 1944 book and chapters I and II of the 1953 book, plus dozens of tables with quantitative data on both).

As another point of correction, Simpson's model continues to be the most well-known and cited model to explain broadscale phenomena in vertebrate evolution as evidenced by multiple studies in the past two decades that continue to use it to explain the major patterns and processes in the fossil record. For just a few examples (Brusatte *et al.* 2014; Benton 2015; Close *et al.* 2015; Slater & Friscia 2019).

Even in the neontological literature, the so-called ecological model of evolution, and the entire concept of ecological opportunity for adaptive radiations, is in fact a co-opted version of Simpson's model, or Simpson's ecospace model, targeting extant populations instead of extinct ones (Benton 2015). As eloquently put by Stroud & Losos (2016): "*The notion of ecological opportunity as a prerequisite for adaptive radiation stems from the pioneering work of George G. Simpson (1953), in which he defined ecological opportunity as an environment experienced by an ancestral species that was previously "occupied by organisms for some reason competitively inferior to the entering group or must be empty" (p. 207).*". We reiterate that there is, and there has been only one predominant concept of adaptive radiation in the paleontological literature (Simpson's), variations of which have been adapted to extant organisms [e.g., (Schluter 2000)], but which are irrelevant to the questions raised here: understanding the evolution and origin of major clades in deep time as originally envisioned by Simpson.

II) Importantly, we are not testing the Simpsonian or any other evolution model per se. We are simply reporting our results and indicating which previously proposed evolutionary models (adaptive vs constructive radiations) offer the best explanations for the pattern in the results presented here. We have reframed our Introduction section to make it clearer.

III) Finally, we utilize recently developed quantitative tools to explore rates of evolution that have rarely been explored in the fossil record, besides demonstrating previously unrecognized limitations of the major software package for evolutionary analysis of this kind. We do not see how, in any context, this study is "framed in the past". We have also made this clearer in the Introduction.

Referee #3 (Remarks to the Author):

This is an improved version of this contribution and I thank the authors for responding my concerns. However, I still have one major concern: to not explicitly take taxonomic diversification into account. One of the goals of this study was to explore "which periods in early diapsid and lepidosaur evolution can be characterized as adaptive radiations as predicted by the Simpsonian model". The paper is mostly built by discussing the predictions of the Simpsonian model of adaptive radiations. However, I'm afraid the analyses performed are not sufficient to allow them to reach part of their conclusions. A rapidly increase in taxonomic diversification is part of the predictions of the Simpsonian model but the authors do not perform any formal test regarding this macroevolutionary axis. So, basically, they are exploring if - and only if - phenotypic rates and disparity plus rates of molecular evolution change through time and among clades. Without analyzing the pace of taxonomic
diversification, it is not possible to say whether or not clades correspond to adaptive radiations, even when applying the definitions claimed by the authors. Because of that I'm afraid this might prevent the authors to fully understand whether or not the analyzed lineages correspond to adaptive radiations.

Response:

I) We respectfully disagree with the statement above that we did not explicitly take taxonomic diversification into account.:

1) Diversity curves in the fossil record of reptiles have been abundant in the literature for the past 30 years, with several studies been published in the last decade (Sahney & Benton 2008; Benson *et al.* 2009; Benton 2010; Sahney *et al.* 2010; Irmis & Whiteside 2012; Benton *et al.* 2013; Cleary *et al.* 2018; Ezcurra & Butler 2018; Close *et al.* 2019). Many of those studies include a detailed assessment of diversity recovery in the Triassic after the Permian-Triassic mass extinction, all agreeing on a massive increase in reptile diversity in the early stages of the Triassic. This is has become common knowledge in the paleontological literature, and often referred to in several books and textbooks (Benton 2003; Benton 2005; Erwin 2015). Additionally, two very recent studies provide a large scale and detailed account of diversity

curves for all lepidosaurian reptiles specifically, from the Jurassic to the present time, thus encompassing the other major focal group we assess here (Cleary *et al.* 2018; Close *et al.* 2019).

2) The classical model of adaptive radiation (Simpsonian/ecological model) predicts an increase in taxonomic diversity, morphological diversity (disparity) and evolutionary rates—a point that we have made clear in our introduction since the beginning. Despite a huge collective effort on understanding diversity changes in the fossil record of reptiles, there has been comparatively a much lower effort on understanding broad scale changes in evolutionary rates and morphological disparity. This is the missing piece of the equation in order to effectively provide all necessary variables to test adaptive radiations for many groups of reptiles, especially early evolving diapsids and lepidosaurs, which we provide in our study.

3) Among all time periods and different lineages we studied, there is only one instance in which there is a concurrent fast increase in both morphological disparity and evolutionary rates, which are necessary to match the expectations of an adaptive radiation: the early and middle Triassic, following the aftermath of the Permian-Triassic mass extinction. As evidenced in point 1 above, this is one of the most well-studied time periods for diversity changes in reptile evolution. Since our morphological disparity and evolutionary rates results match this well-known increase in reptile diversity in the Triassic (explicitly estimated and modeled by all referenced studies above), we find the adaptive radiation model to be the best explanation for the results obtained here, as long argued by many (Benton 2003; Benton 2005; Erwin 2015).

4) All other timeframes studied here do not find a concurrent increase in morphological disparity and evolutionary rates for reptiles. Hence, these results suggest other evolutionary models may offer better explanations to our results (e.g., constructive radiation in the early evolution of lepidosaurs). Even if there was an increase in taxonomic diversity concurrent with periods of either increase in morphological disparity or rates of evolution, it becomes irrelevant in the face of the fact that it is necessary for all three variables to be in agreement with each other to verify an adaptive radiation.

As we hope to clarify here, the scientific evidence on reptile diversity curves necessary to support the conclusions is already available. Taking previously published results to complement ours and guide our discussions is an essential component to empirical research.

II) Following editorial advice, we have now made our points clear in the main text that i) we do have available data on the taxonomic diversity dynamics of reptiles in deep time; and ii) we have reframed our Introduction section to make it clear that we do not try to test the Simpsonian model of adaptive radiation itself. We are simply reporting our results and indicating which previously proposed evolutionary models (adaptive vs constructive radiations) offer the best explanations for the pattern in the results presented here for each individual timeframe.

References cited above:

Benson, R. B. J., Butler, R. J., Lindgren, J. & Smith, A. S. 2009. Mesozoic marine tetrapod diversity: mass extinctions and temporal heterogeity in geological megabiases affecting vertebrates. *Proceedings of the Royal Society. Biological Sciences*, **277**(1683), 829 - 834.
Benton, M. J. 2003. *When life nearly died: the greatest mass extinction of all time*. Thames & Hudson.
Benton, M. J. 2005. *Vertebrate Paleontology*. Blackwell, 455 pp.
Benton, M. J. 2010. The origins of modern biodiversity on land. *Philosophical Transactions of the Royal Society B: Biological Sciences*, **365**(1558), 3667-3679.
Benton, M. J. 2015. Exploring macroevolution using modern and fossil data. *Proceedings of the Royal Society B: Biological Sciences*, **282**(1810), 20150569.
Benton, M. J., Ruta, M., Dunhill, A. M. & Sakamoto, M. 2013. The first half of tetrapod evolution, sampling proxies, and fossil record quality. *Palaeogeography, Palaeoclimatology, Palaeoecology*, **372**, 18-41.
Brusatte, S. L., Lloyd, G. T., Wang, S. C. & Norell, M. A. 2014. Gradual Assembly of Avian Body Plan Culminated in Rapid Rates of Evolution across the Dinosaur-Bird Transition. *Current Biology*, **24**(20), 2386-2392.
Cleary, T. J., Benson, R. B., Evans, S. E. & Barrett, P. M. 2018. Lepidosaurian diversity in the Mesozoic–Palaeogene: the potential roles of sampling biases and environmental drivers. *Royal Society Open Science*, **5**(3), 171830.
Close, R. A., Benson, R. B. J., Alroy, J., Behrensmeyer, A. K., Benito, J., Carrano, M. T., Cleary, T. J., Dunne, E. M., Mannion, P. D., Uhen, M. D. & Butler, R. J. 2019. Diversity dynamics of Phanerozoic terrestrial tetrapods at the local-community scale. *Nature Ecology & Evolution*.
Close, R. A., Friedman, M., Lloyd, G. T. & Benson, R. B. 2015. Evidence for a mid-Jurassic adaptive radiation in mammals. *Current Biology*, **25**(16), 2137-2142.
Erwin, D. H. 2015. *Extinction–How Life on Earth Nearly Ended 250 Million Years Ago. Updated Edition*. Princeton University Press, Princeton.
Ezcurra, M. D. & Butler, R. J. 2018. The rise of the ruling reptiles and ecosystem recovery from the Permo-Triassic mass extinction. *Proc. R. Soc. B*, **285**(1880), 20180361.
Irmis, R. B. & Whiteside, J. H. 2012. Delayed recovery of non-marine tetrapods after the end-Permian mass extinction tracks global carbon cycle. *Proceedings of the Royal Society B: Biological Sciences*, **279**(1732), 1310-1318.
Luo, A., Duchêne, D. A., Zhang, C., Zhu, C.-D. & Ho, S. Y. W. 2020. A Simulation-Based Evaluation of Tip-Dating Under the Fossilized Birth–Death Process. *Systematic Biology*, **69**(2), 325–344.
Paterson, J. R., Edgecombe, G. D. & Lee, M. S. Y. 2019. Trilobite evolutionary rates constrain the duration of the Cambrian explosion. *Proceedings of the National Academy of Sciences*, **116**(10), 4394-4399.
Sahney, S. & Benton, M. J. 2008. Recovery from the most profound mass extinction of all time. *Proceedings of the Royal Society of London B: Biological Sciences*, **275**(1636), 759-765.
Sahney, S., Benton, M. J. & Ferry, P. A. 2010. Links between global taxonomic diversity, ecological diversity and the expansion of vertebrates on land. *Biology Letters*, **6**(4), 544-547.
Schluter, D. 2000. Ecological Character Displacement in Adaptive Radiation. *The American Naturalist*, **156**(S4), S4-S16.
Simpson, G. G. 1944. *Tempo and mode in evolution*. Columbia University Press.
Simpson, G. G. 1953. *The Major Features of Evolution*. Columbia University Press, New York, 434 pp.
Slater, G. J. & Friscia, A. R. 2019. Hierarchy in adaptive radiation: a case study using the Carnivora (Mammalia). *Evolution*, **73**(3), 524-539.
Stroud, J. T. & Losos, J. B. 2016. Ecological opportunity and adaptive radiation. *Annual Review of Ecology, Evolution, and Systematics*, **47**, 507-532.

REVIEWERS' COMMENTS:

Reviewer #3 (Remarks to the Author):

I thank the authors for clarifying my concerns regarding the pace of taxonomic diversification and I commend them for providing a detailed list of references to support their arguments. Previous versions of the ms simply did not comprise enough information to allow the reader to properly interpret the goals and main conclusions - although it has been clear that the authors dominate concepts and theory. It is important to bear in mind that Nature Communications has a very broad audience and the changes made by the authors in their main text were therefore essential. I am now happy to recommend this manuscript for publication in Nature Communications.
