# Supplementary material for: Megaevolutionary dynamics and the timing of evolutionary innovation in reptiles
Source: Nat Commun. 2020 Jul 3;11:3322. doi: 10.1038/s41467-020-17190-9 (PMC7335191; doi:10.1038/s41467-020-17190-9)
Supplement: Supplementary file 5 — Supplementary Data 1 − 5 [file 41467_2020_17190_MOESM5_ESM.zip › SD5_Stats.pdf]

Early diapsids

Morphological rates

Original data

	Epoch	n	mean	sd	min	Q1	median	Q3	max
1	Cisuralian	21	2.411	1.986	0.076	0.323	2.340	3.370	5.97
2	Guadalupian	18	1.812	1.789	0.060	0.725	1.495	1.968	7.45
3	Lopingian	23	1.557	1.108	0.018	0.680	1.630	2.010	4.81
4	Early Triassic	18	1.868	0.941	0.642	1.292	1.700	2.513	4.24
5	Middle Triassic	29	2.629	2.643	0.001	0.698	1.970	3.330	13.20

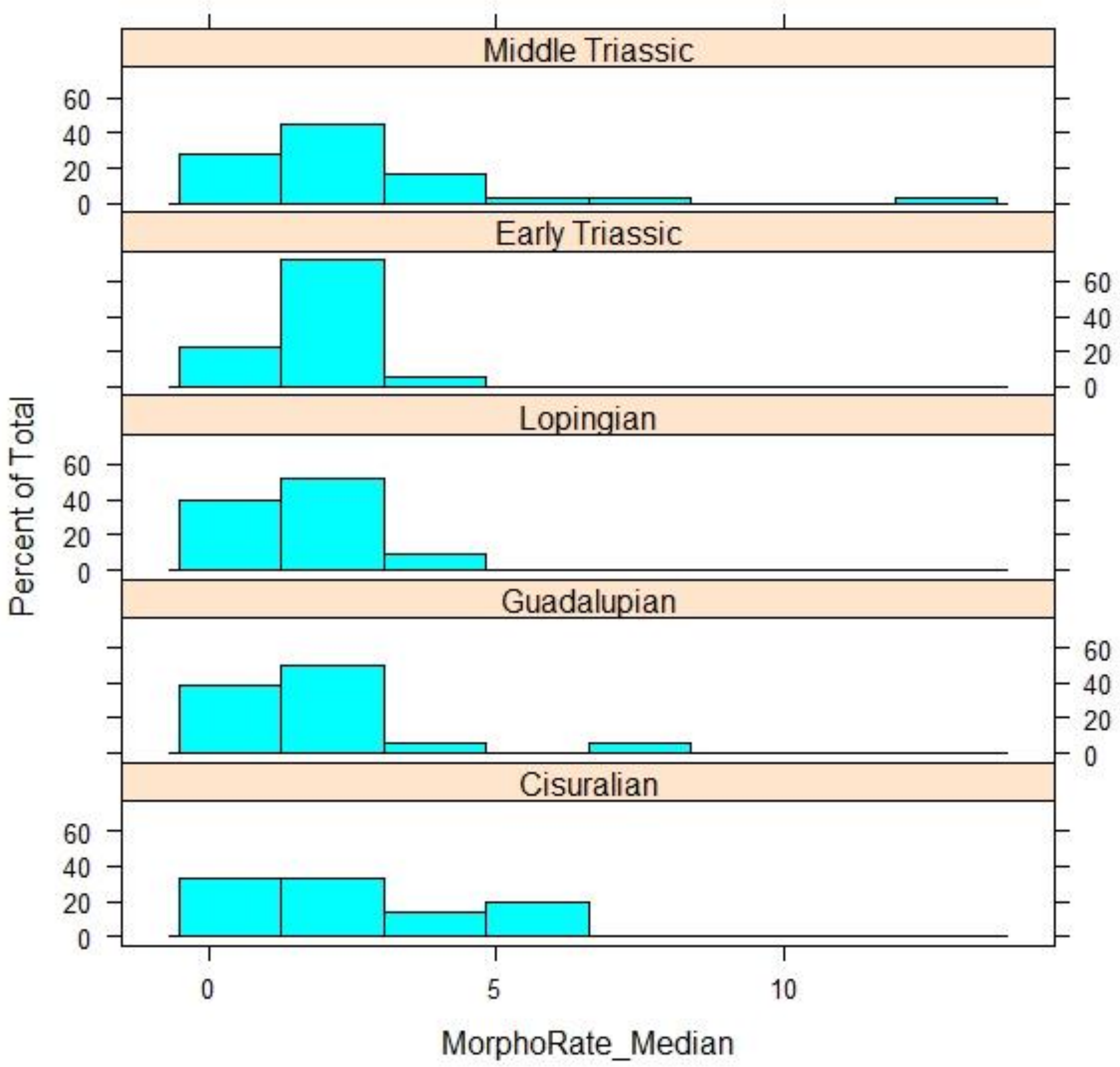

Shapiro-Wilk normality test (MorphoRates)

```
$Cisuralian

	Shapiro-Wilk normality test

data:  X[[i]]
W = 0.8998, p-value = 0.03469


$Guadalupian

	Shapiro-Wilk normality test

data:  X[[i]]
W = 0.76912, p-value = 0.0005744


$Lopingian

	Shapiro-Wilk normality test

data:  X[[i]]
W = 0.92187, p-value = 0.07306


$`Early Triassic`

	Shapiro-Wilk normality test

data:  X[[i]]
W = 0.93318, p-value = 0.2208


$`Middle Triassic`

	Shapiro-Wilk normality test

data:  X[[i]]
W = 0.7561, p-value = 1.498e-05
```

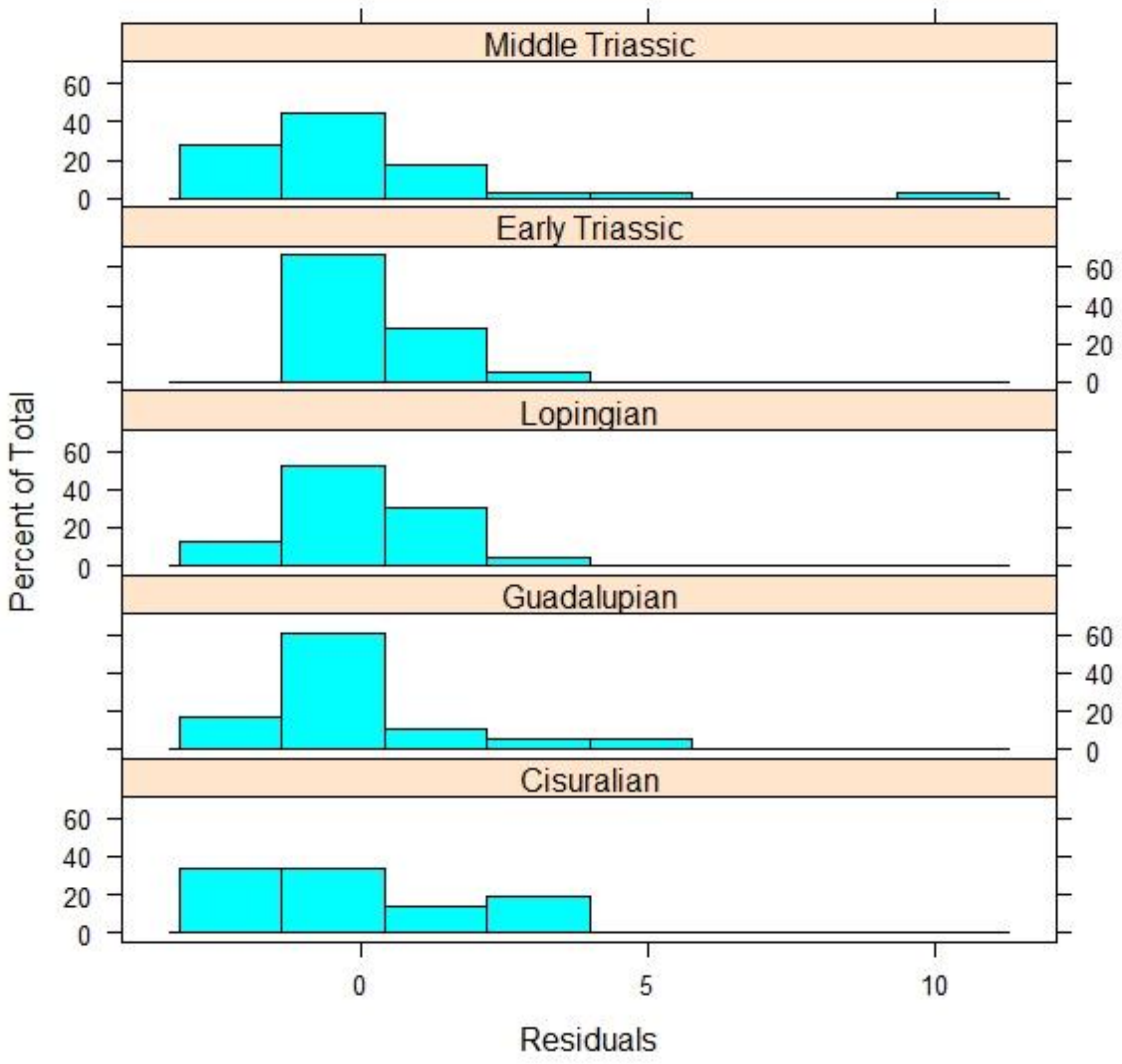

Shapiro-Wilk normality test (Residuals)

```
$Cisuralian

        Shapiro-Wilk normality test

data:  X[[i]]
W = 0.8998, p-value = 0.03469


$Guadalupian

        Shapiro-Wilk normality test

data:  X[[i]]
W = 0.76912, p-value = 0.0005744
```

```
$Lopingian

	Shapiro-Wilk normality test

data:  X[[i]]
W = 0.92187, p-value = 0.07306


$`Early Triassic`

	Shapiro-Wilk normality test

data:  X[[i]]
W = 0.93318, p-value = 0.2208


$`Middle Triassic`

	Shapiro-Wilk normality test

data:  X[[i]]
W = 0.7561, p-value = 1.498e-05
```

Fligner-Killeen test of homogeneity of variances

```
data:  Residuals by Epoch
Fligner-Killeen:med chi-squared = 9.5166, df = 4, p-value = 0.04941
```

Square Cube transformed data

	Epoch	n	mean	sd	min	Q1	median	Q3	max
1	Cisuralian	21	1.188	0.476	0.424	0.686	1.328	1.499	1.814
2	Guadalupian	18	1.106	0.381	0.391	0.897	1.143	1.253	1.953
3	Lopingian	23	1.074	0.332	0.263	0.879	1.177	1.262	1.688
4	Early Triassic	18	1.199	0.206	0.863	1.089	1.191	1.359	1.619
5	Middle Triassic	29	1.232	0.470	0.105	0.887	1.254	1.493	2.36

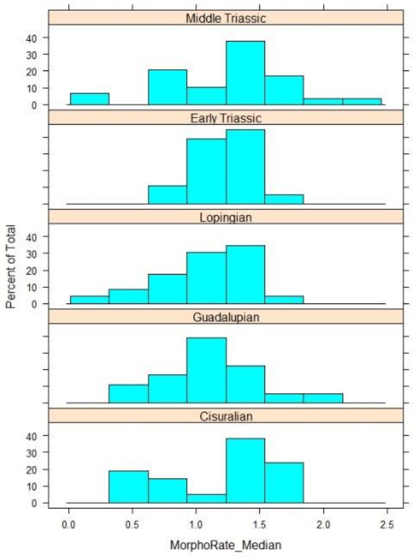

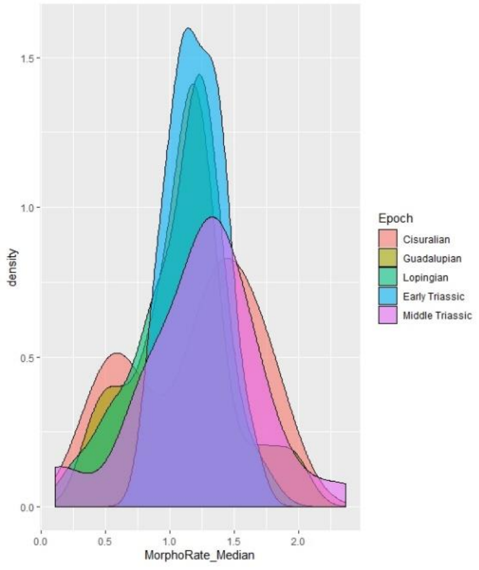

```
        Shapiro-Wilk normality test BY LEVEL (MorphoRate)


$Cisuralian

        Shapiro-Wilk normality test

data:  X[[i]]
W = 0.89004, p-value = 0.02254


$Guadalupian

        Shapiro-Wilk normality test

data:  X[[i]]
W = 0.96387, p-value = 0.6778


$Lopingian

        Shapiro-Wilk normality test

data:  X[[i]]
W = 0.94333, p-value = 0.2117


$`Early Triassic`

        Shapiro-Wilk normality test

data:  X[[i]]
W = 0.97321, p-value = 0.8555


$`Middle Triassic`

        Shapiro-Wilk normality test

data:  X[[i]]
W = 0.95442, p-value = 0.238
```

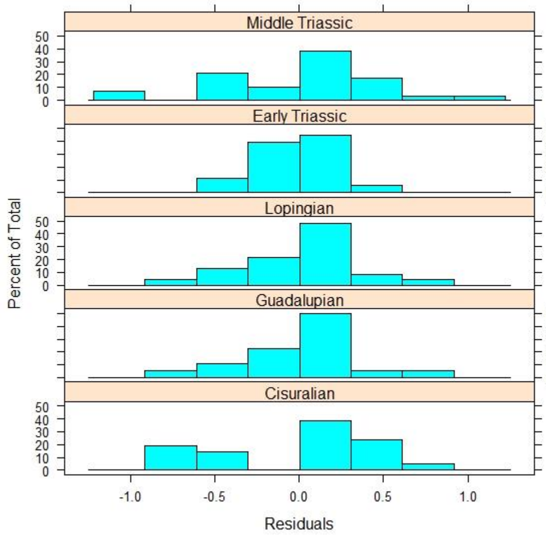

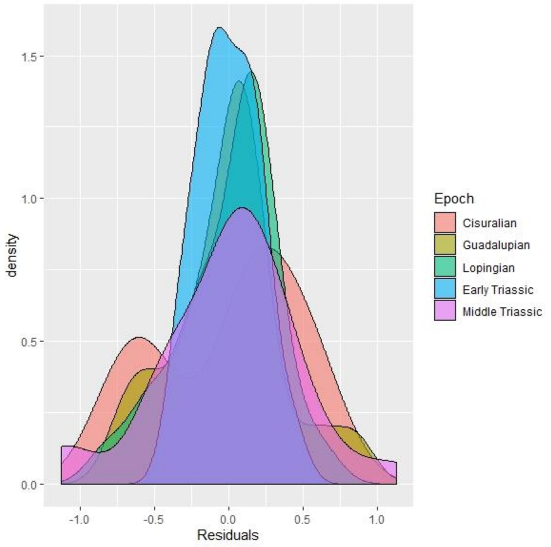

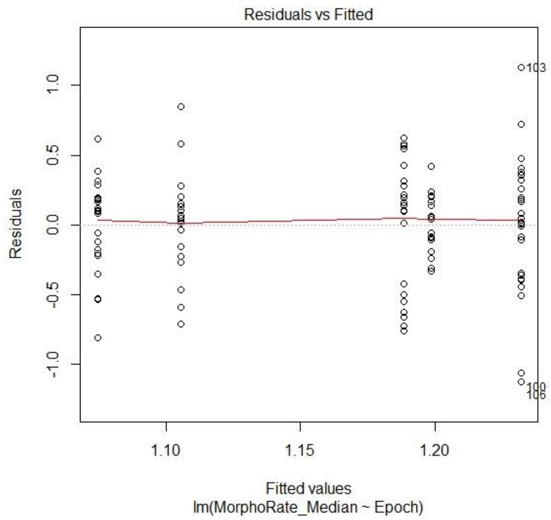

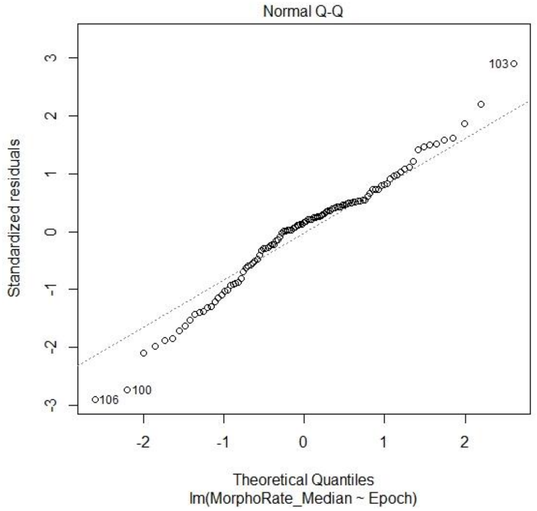


```
        Shapiro-Wilk normality test BY LEVEL (RESIDUALS)

$Cisuralian

        Shapiro-Wilk normality test

data:  X[[i]]
W = 0.89004, p-value = 0.02254


$Guadalupian

        Shapiro-Wilk normality test

data:  X[[i]]
W = 0.96387, p-value = 0.6778
```

```
$Lopingian

        Shapiro-Wilk normality test

data:  X[[i]]
W = 0.94333, p-value = 0.2117


$`Early Triassic`

        Shapiro-Wilk normality test

data:  X[[i]]
W = 0.97321, p-value = 0.8555


$`Middle Triassic`

        Shapiro-Wilk normality test

data:  X[[i]]
W = 0.95442, p-value = 0.238
```

Fligner-Killeen test of homogeneity of variances

```
data:  Residuals by Epoch
Fligner-Killeen:med chi-squared = 5.4858, df = 4, p-value = 0.241
```

Decision: Parametric test

Pairwise t-tests

	Cisuralian	Guadalupian	Lopingian	Early Triassic
Guadalupian	0.861716	NA	NA	NA
Lopingian	0.858289	0.892796	NA	NA
Early Triassic	0.934988	0.861716	0.858289	NA
Middle Triassic	0.892796	0.858289	0.858289	0.892796

Lepidosaurs

Morphological rates

Original data

	Epoch	n	nvalid	mean	sd	min	Q1	median	Q3	max
1	J1 (Hatte-Aelen)	18	16	1.348	1.233	0.029	0.505	0.932	1.862	4.05
2	J2 (Bajo-Tithon)	24	22	2.498	6.455	0.178	0.318	0.445	0.814	26.60
3	Early Cretaceous	46	46	0.658	0.887	0.081	0.196	0.308	0.698	3.89
4	LK1 (Ceno-Sant)	21	20	0.670	0.504	0.089	0.230	0.561	1.038	1.44
5	LK2 (Camp-Maast)	26	21	1.277	1.048	0.066	0.581	0.989	1.640	3.91
6	Paleocene	12	12	0.880	0.891	0.027	0.472	0.658	0.931	3.29
7	Early Eocene	25	24	0.778	1.065	0.021	0.060	0.446	0.998	4.99
8	Extant	47	47	0.564	0.360	0.272	0.378	0.441	0.632	2.41

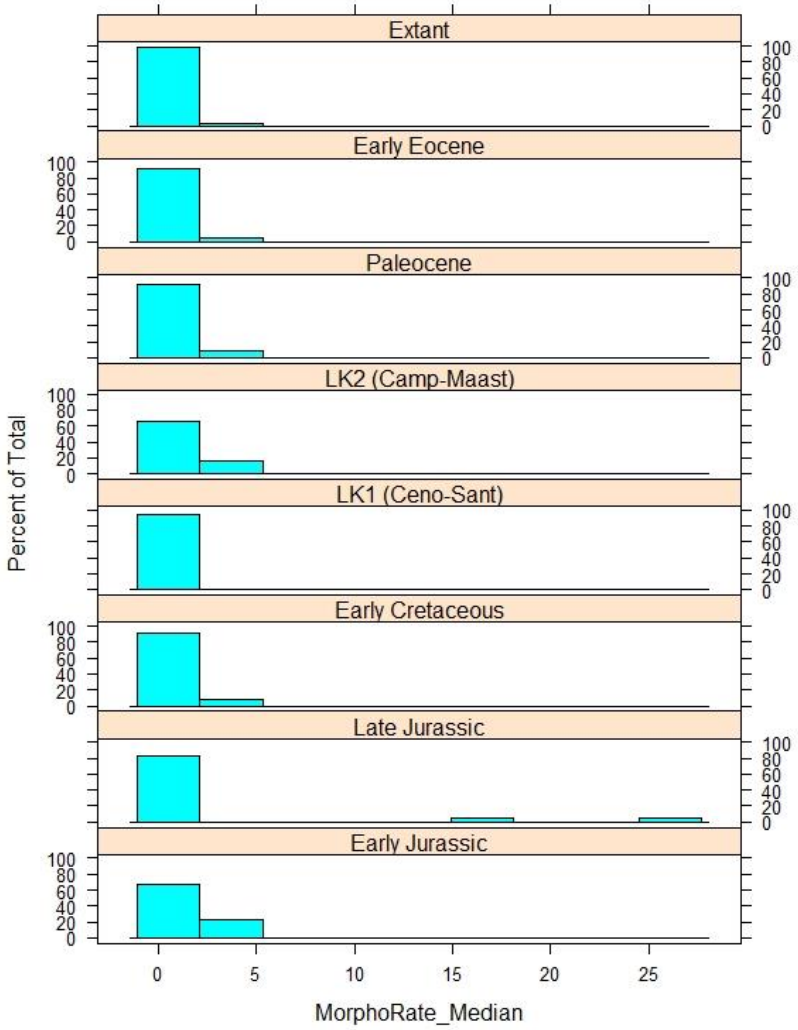

Shapiro-Wilk normality test ALL LEVELS (MorphoRate)

```
$`J1 (Hatte-Aelen)`

        Shapiro-Wilk normality test

data:  X[[i]]
W = 0.89939, p-value = 0.07858


$`J2 (Bajo-Tithon)`

        Shapiro-Wilk normality test

data:  X[[i]]
W = 0.38567, p-value = 1.304e-08


$`Early Cretaceous`

        Shapiro-Wilk normality test

data:  X[[i]]
W = 0.62073, p-value = 1.146e-09


$`LK1 (Ceno-Sant)`

        Shapiro-Wilk normality test

data:  X[[i]]
W = 0.86429, p-value = 0.00934


$`LK2 (Camp-Maast)`

        Shapiro-Wilk normality test

data:  X[[i]]
W = 0.88805, p-value = 0.02066


$Paleocene

        Shapiro-Wilk normality test

data:  X[[i]]
W = 0.76701, p-value = 0.004046


$`Early Eocene`

        Shapiro-Wilk normality test

data:  X[[i]]
W = 0.67398, p-value = 4.649e-06


$Extant

        Shapiro-Wilk normality test

data:  X[[i]]
W = 0.64027, p-value = 1.746e-09
```

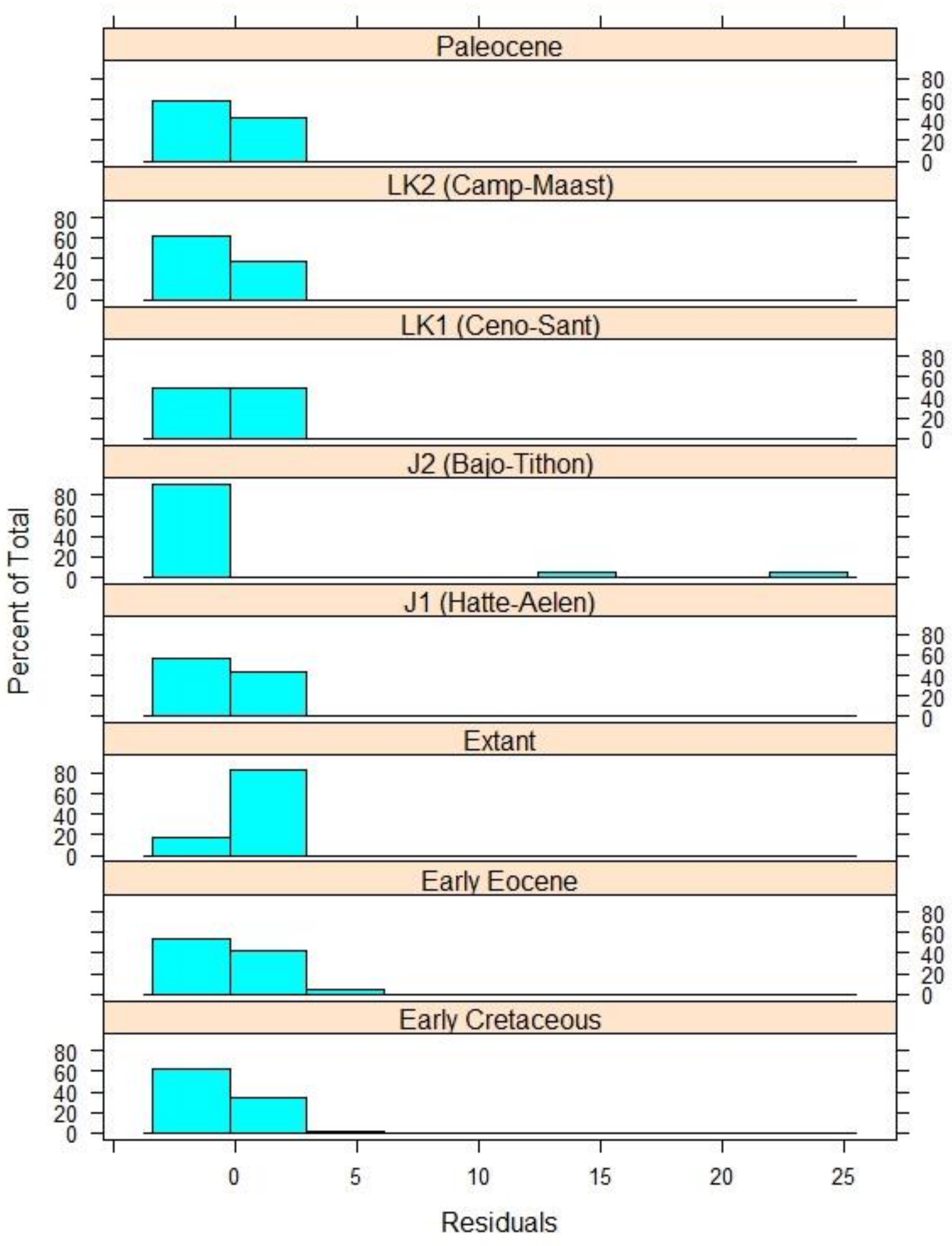

Shapiro-Wilk normality test ALL LEVEL (RESIDUALS)

```
$`J1 (Hatte-Aelen)`

        Shapiro-Wilk normality test

data:  X[[i]]
W = 0.89939, p-value = 0.07858


$`J2 (Bajo-Tithon)`

        Shapiro-Wilk normality test

data:  X[[i]]
W = 0.38567, p-value = 1.304e-08


$`Early Cretaceous`

        Shapiro-Wilk normality test

data:  X[[i]]
W = 0.62073, p-value = 1.146e-09


$`LK1 (Ceno-Sant)`

        Shapiro-Wilk normality test

data:  X[[i]]
W = 0.86429, p-value = 0.00934


$`LK2 (Camp-Maast)`

        Shapiro-Wilk normality test

data:  X[[i]]
W = 0.88805, p-value = 0.02066


$Paleocene

        Shapiro-Wilk normality test

data:  X[[i]]
W = 0.76701, p-value = 0.004046


$`Early Eocene`

        Shapiro-Wilk normality test

data:  X[[i]]
W = 0.67398, p-value = 4.649e-06


$Extant

        Shapiro-Wilk normality test

data:  X[[i]]
W = 0.64027, p-value = 1.746e-09
```

Cube root Transformation

	Epoch	n	mean	sd	min	Q1	median	Q3	max
1	J1 (Hatte-Aelen)	16	0.973	0.402	0.309	0.776	0.977	1.228	1.594
2	J2 (Bajo-Tithon)	22	0.972	0.609	0.563	0.683	0.763	0.933	2.985
3	Early Cretaceous	46	0.763	0.286	0.432	0.580	0.675	0.887	1.573
4	LK1 (Ceno-Sant)	20	0.809	0.248	0.446	0.612	0.815	1.012	1.129
5	LK2 (Camp-Maast)	21	0.998	0.317	0.404	0.834	0.996	1.179	1.575
6	Paleocene	12	0.857	0.327	0.299	0.778	0.870	0.976	1.487
7	Early Eocene	24	0.768	0.369	0.276	0.391	0.763	0.999	1.709
8	Extant	47	0.804	0.131	0.648	0.723	0.761	0.858	1.341

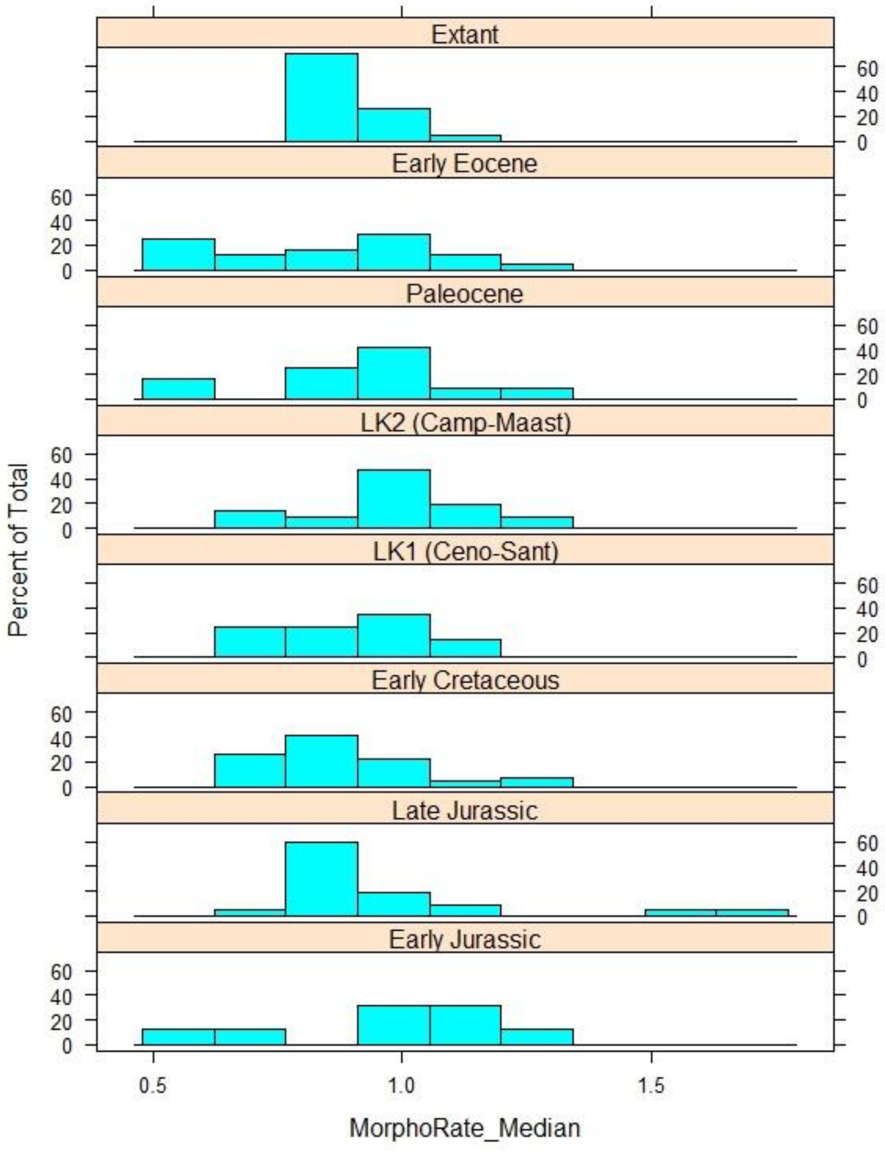

Shapiro-Wilk normality test ALL LEVELS (MorphoRate)

```
$`J1 (Hatte-Aelen)`

        Shapiro-Wilk normality test

data:  X[[i]]
W = 0.95111, p-value = 0.5074


$`J2 (Bajo-Tithon)`

        Shapiro-Wilk normality test

data:  X[[i]]
W = 0.56489, p-value = 5.682e-07


$`Early Cretaceous`

        Shapiro-Wilk normality test

data:  X[[i]]
W = 0.86075, p-value = 5.924e-05


$`LK1 (Ceno-Sant)`

        Shapiro-Wilk normality test

data:  X[[i]]
W = 0.88838, p-value = 0.02511


$`LK2 (Camp-Maast)`

        Shapiro-Wilk normality test

data:  X[[i]]
W = 0.97144, p-value = 0.7647


$Paleocene

        Shapiro-Wilk normality test

data:  X[[i]]
W = 0.91889, p-value = 0.2769


$`Early Eocene`

        Shapiro-Wilk normality test

data:  X[[i]]
W = 0.93412, p-value = 0.1205


$Extant

        Shapiro-Wilk normality test

data:  X[[i]]
W = 0.83175, p-value = 8.856e-06
```

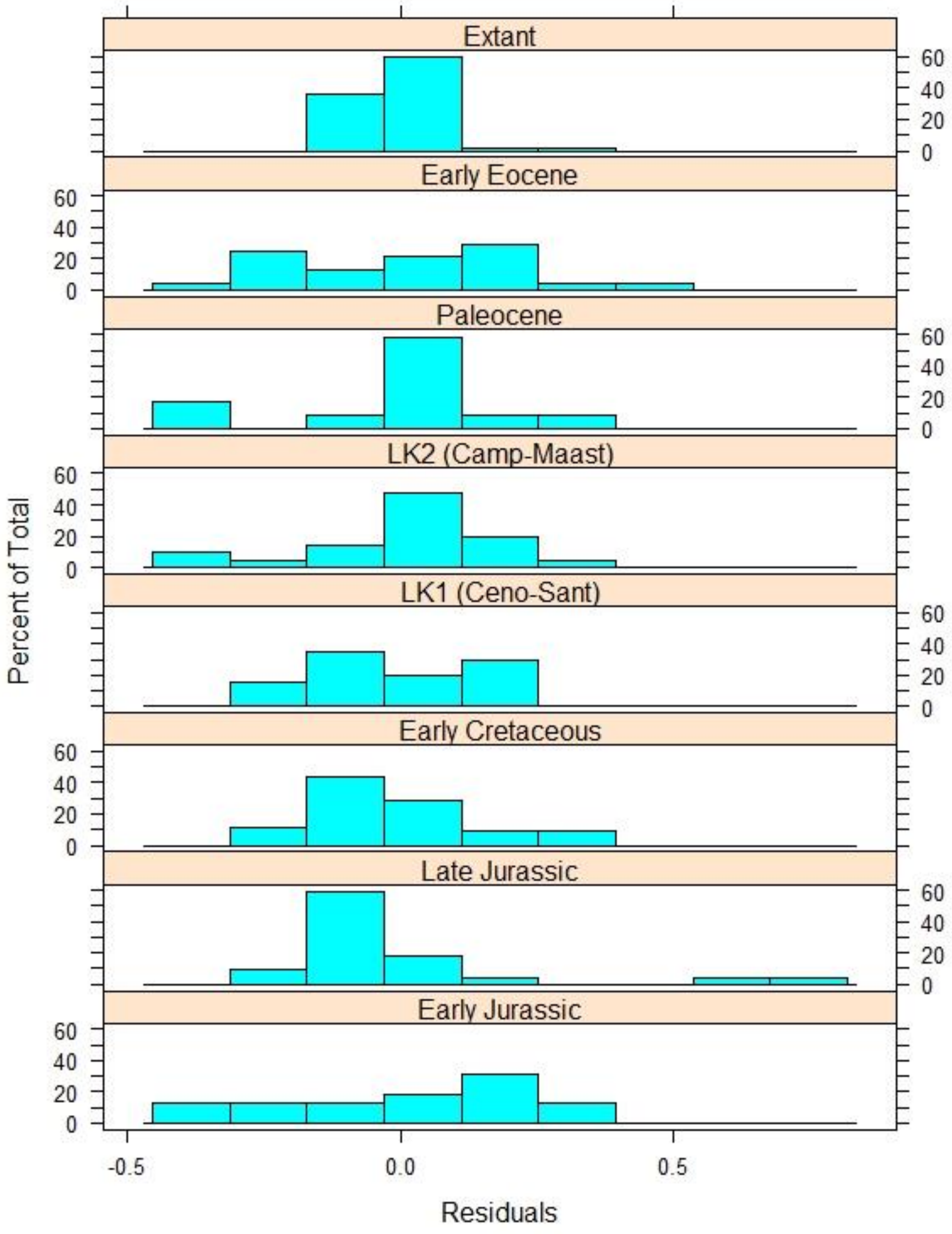

Extant
Early Eocene
Paleocene
LK2 (Camp-Maast)
LK1 (Ceno-Sant)
Early Cretaceous
Late Jurassic
Early Jurassic
Percent of Total
0
20
40
60
-0.5
0.0
0.5
Residuals

```
                Shapiro-Wilk normality test ALL LEVEL (RESIDUALS)

data:  residuals(model)
W = 0.9581, p-value = 8.398e-06


        Shapiro-Wilk normality test BY LEVEL (MorphoRate)
$`J1 (Hatte-Aelen)`

        Shapiro-Wilk normality test

data:  X[[i]]
W = 0.95111, p-value = 0.5074


$`J2 (Bajo-Tithon)`

        Shapiro-Wilk normality test

data:  X[[i]]
W = 0.56489, p-value = 5.682e-07


$`Early Cretaceous`

        Shapiro-Wilk normality test

data:  X[[i]]
W = 0.86075, p-value = 5.924e-05


$`LK1 (Ceno-Sant)`

        Shapiro-Wilk normality test

data:  X[[i]]
W = 0.88838, p-value = 0.02511


$`LK2 (Camp-Maast)`

        Shapiro-Wilk normality test

data:  X[[i]]
W = 0.97144, p-value = 0.7647


$Paleocene

        Shapiro-Wilk normality test

data:  X[[i]]
W = 0.91889, p-value = 0.2769


$`Early Eocene`

        Shapiro-Wilk normality test

data:  X[[i]]
W = 0.93412, p-value = 0.1205
```

```
$Extant

        Shapiro-Wilk normality test

data:  X[[i]]
W = 0.83175, p-value = 8.856e-06


        Fligner-Killeen test of homogeneity of variances

data:  Residuals by Epoch
Fligner-Killeen:med chi-squared = 35.191, df = 7, p-value = 1.03e-05
```

Decision: Nonparametric test

Pairwise comparisons using Wilcoxon rank sum test

	J1 (Hatte-Aelen)	J2 (Bajo-Tithon)	Early Cretaceous	LK1 (Ceno-Sant)	LK2 (Camp-Maast)	Paleocene	Early Eocene
J2 (Bajo-Tithon)	0.417792	NA	NA	NA	NA	NA	NA
Early Cretaceous	0.212601	0.212601	NA	NA	NA	NA	NA
LK1 (Ceno-Sant)	0.43402	0.738575	0.494622	NA	NA	NA	NA
LK2 (Camp-Maast)	0.986053	0.220719	**0.038567**	0.288567	NA	NA	NA
Paleocene	0.52818	0.52818	0.350619	0.986053	0.350619	NA	NA
Early Eocene	0.350619	0.6128	0.995064	0.661069	0.136851	0.83368	NA
Extant	0.136851	0.995064	0.136851	0.986053	**0.038567**	0.243536	0.876402

Lepidosaurs

Molecular rates

Original data

	Epoch	n	nvalid	mean	sd	min	Q1	median	Q3	max
1	J1 (Hatte-Aelen)	18	16	1.624	1.447	0.292	0.409	1.095	2.158	4.490
2	J2 (Bajo-Tithon)	24	22	0.349	0.215	0.078	0.127	0.400	0.443	0.825
3	Early Cretaceous	46	46	0.432	0.301	0.060	0.181	0.396	0.558	1.390
4	LK1 (Ceno-Sant)	21	20	0.574	0.232	0.099	0.494	0.588	0.714	1.010
5	LK2 (Camp-Maast)	26	21	0.731	0.960	0.151	0.235	0.450	0.700	4.390
6	Paleocene	12	12	1.130	1.053	0.017	0.350	0.750	1.528	3.340
7	Early Eocene	25	24	1.562	2.159	0.201	0.496	0.728	2.277	10.700
8	Extant	47	47	0.955	0.497	0.002	0.638	0.833	1.260	2.440

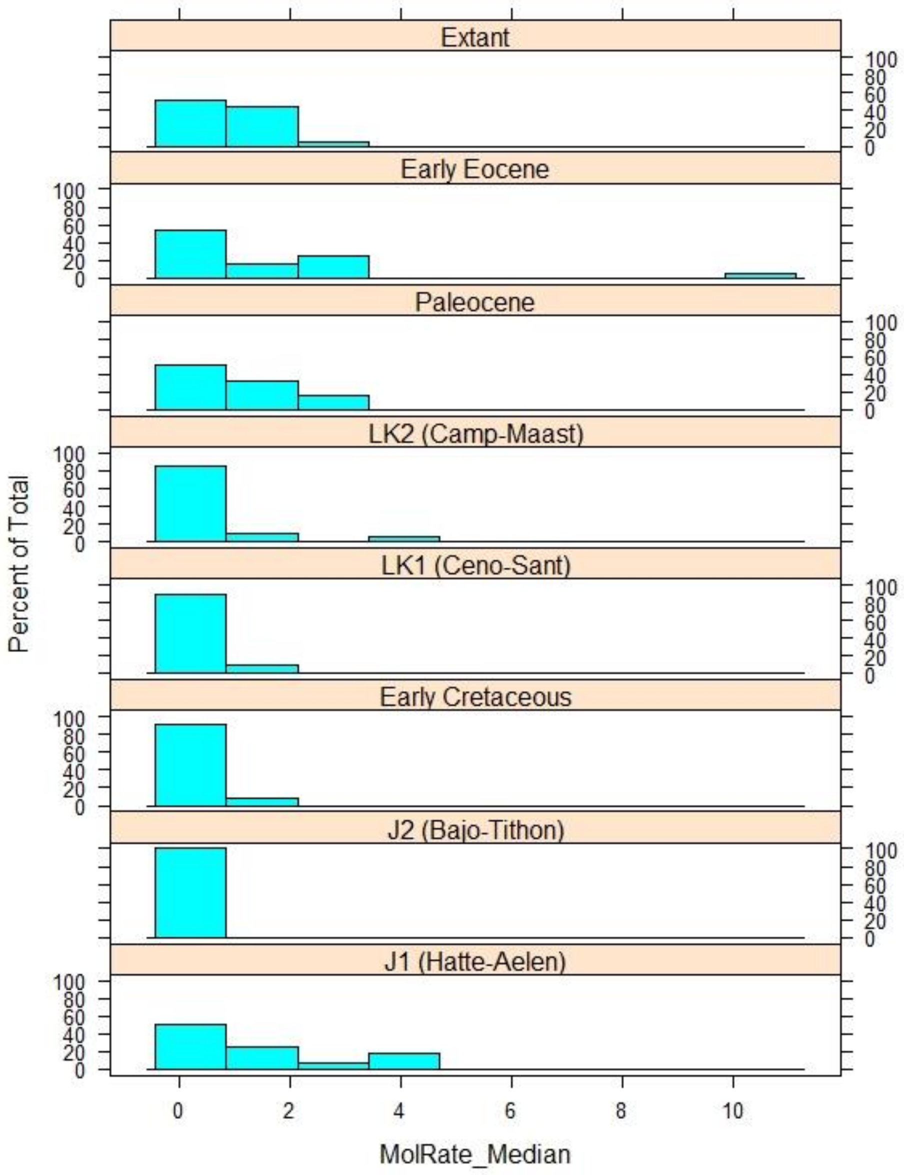

```
        Shapiro-Wilk normality test BY LEVEL (MoloRate)


$`J1 (Hatte-Aelen)`

        Shapiro-Wilk normality test

data:  X[[i]]
W = 0.83101, p-value = 0.007248


$`J2 (Bajo-Tithon)`

        Shapiro-Wilk normality test

data:  X[[i]]
W = 0.91379, p-value = 0.0566


$`Early Cretaceous`

        Shapiro-Wilk normality test

data:  X[[i]]
W = 0.91032, p-value = 0.001775


$`LK1 (Ceno-Sant)`

        Shapiro-Wilk normality test

data:  X[[i]]
W = 0.95348, p-value = 0.423


$`LK2 (Camp-Maast)`

        Shapiro-Wilk normality test

data:  X[[i]]
W = 0.57286, p-value = 1.03e-06


$Paleocene

        Shapiro-Wilk normality test

data:  X[[i]]
W = 0.86772, p-value = 0.06114


$`Early Eocene`

        Shapiro-Wilk normality test

data:  X[[i]]
W = 0.5574, p-value = 2.139e-07
```

```
$Extant

        Shapiro-Wilk normality test

data:  X[[i]]
W = 0.9455, p-value = 0.02894
```

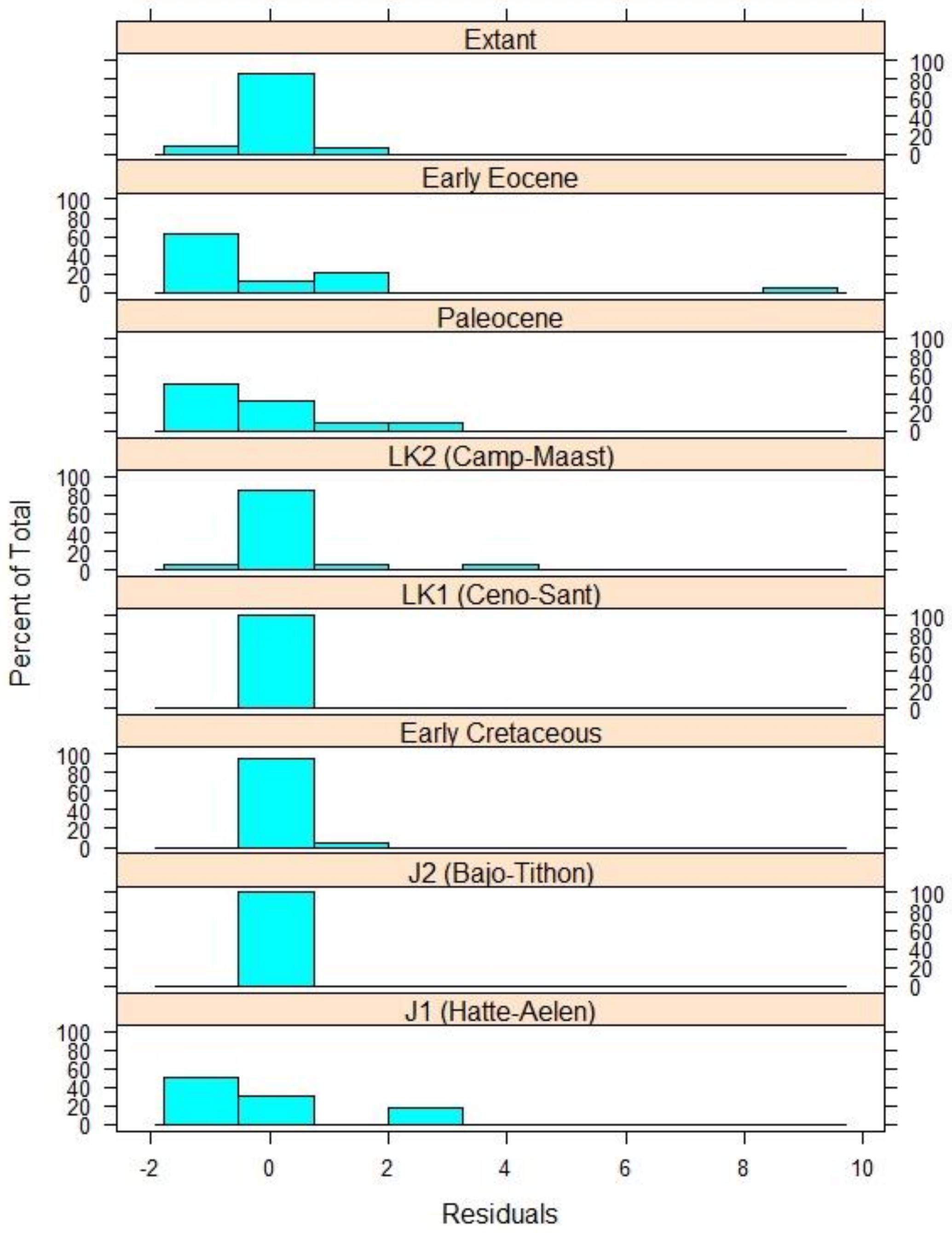

```
        Shapiro-Wilk normality test BY LEVEL (RESIDUALS)

$`J1 (Hatte-Aelen)`

        Shapiro-Wilk normality test

data:  X[[i]]
W = 0.83101, p-value = 0.007248


$`J2 (Bajo-Tithon)`

        Shapiro-Wilk normality test

data:  X[[i]]
W = 0.91379, p-value = 0.0566


$`Early Cretaceous`

        Shapiro-Wilk normality test

data:  X[[i]]
W = 0.91032, p-value = 0.001775


$`LK1 (Ceno-Sant)`

        Shapiro-Wilk normality test

data:  X[[i]]
W = 0.95348, p-value = 0.423


$`LK2 (Camp-Maast)`

        Shapiro-Wilk normality test

data:  X[[i]]
W = 0.57286, p-value = 1.03e-06


$Paleocene

        Shapiro-Wilk normality test

data:  X[[i]]
W = 0.86772, p-value = 0.06114


$`Early Eocene`

        Shapiro-Wilk normality test

data:  X[[i]]
W = 0.5574, p-value = 2.139e-07


$Extant

        Shapiro-Wilk normality test

data:  X[[i]]
```

```
W = 0.9455, p-value = 0.02894
```

Log Transformation

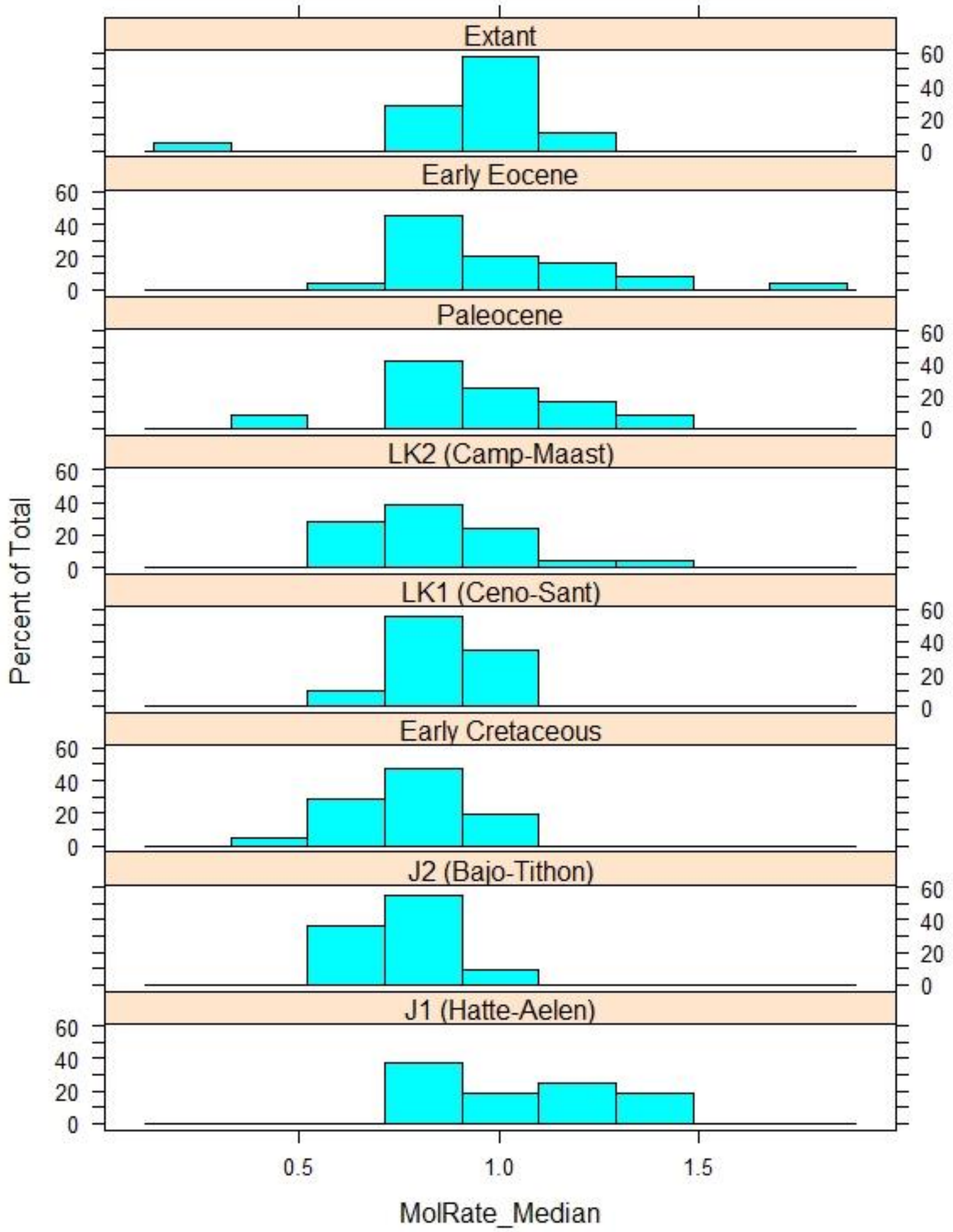

```
Shapiro-Wilk normality test BY LEVEL (MolRate)
$`J1 (Hatte-Aelen)`

	Shapiro-Wilk normality test

data:  X[[i]]
W = 0.90917, p-value = 0.1128


$`J2 (Bajo-Tithon)`

	Shapiro-Wilk normality test

data:  X[[i]]
W = 0.88711, p-value = 0.01656


$`Early Cretaceous`

	Shapiro-Wilk normality test

data:  X[[i]]
W = 0.95928, p-value = 0.1075


$`LK1 (Ceno-Sant)`

	Shapiro-Wilk normality test

data:  X[[i]]
W = 0.78223, p-value = 0.0004749


$`LK2 (Camp-Maast)`

	Shapiro-Wilk normality test

data:  X[[i]]
W = 0.91109, p-value = 0.05771


$Paleocene

	Shapiro-Wilk normality test

data:  X[[i]]
W = 0.8652, p-value = 0.05683


$`Early Eocene`

	Shapiro-Wilk normality test

data:  X[[i]]
W = 0.92135, p-value = 0.06257


$Extant

	Shapiro-Wilk normality test

data:  X[[i]]
W = 0.50515, p-value = 2.286e-11
```

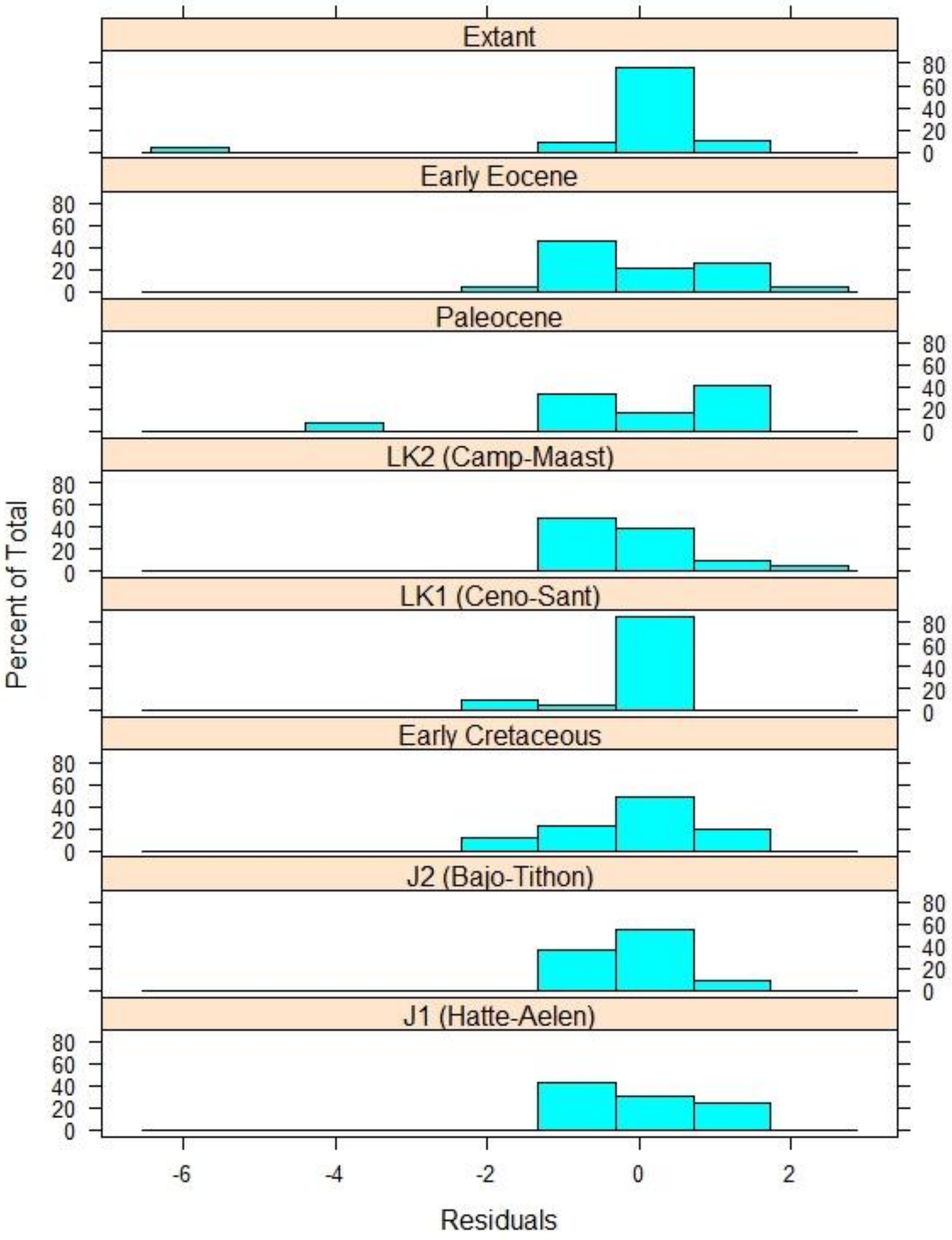
Extant
Early Eocene
Paleocene
LK2 (Camp-Maast)
LK1 (Ceno-Sant)
Early Cretaceous
J2 (Bajo-Tithon)
J1 (Hatte-Aelen)
Percent of Total
Residuals
0
20
40
60
80
-6
-4
-2
0
2

```
                Shapiro-Wilk normality test BY LEVEL (RESIDUALS)

$`J1 (Hatte-Aelen)`

        Shapiro-Wilk normality test

data:  X[[i]]
W = 0.90917, p-value = 0.1128


$`J2 (Bajo-Tithon)`

        Shapiro-Wilk normality test

data:  X[[i]]
W = 0.88711, p-value = 0.01656


$`Early Cretaceous`

        Shapiro-Wilk normality test

data:  X[[i]]
W = 0.95928, p-value = 0.1075


$`LK1 (Ceno-Sant)`

        Shapiro-Wilk normality test

data:  X[[i]]
W = 0.78223, p-value = 0.0004749


$`LK2 (Camp-Maast)`

        Shapiro-Wilk normality test

data:  X[[i]]
W = 0.91109, p-value = 0.05771


$Paleocene

        Shapiro-Wilk normality test

data:  X[[i]]
W = 0.8652, p-value = 0.05683


$`Early Eocene`

        Shapiro-Wilk normality test

data:  X[[i]]
W = 0.92135, p-value = 0.06257


$Extant

        Shapiro-Wilk normality test

data:  X[[i]]
W = 0.50515, p-value = 2.286e-11
```

Fligner-Killeen test of homogeneity of variances

data: Residuals by Epoch
Fligner-Killeen:med chi-squared = 18.87, df = 7, p-value = 0.008604

Decision: Nonparametric test

Pairwise comparisons using Wilcoxon rank sum test

	J1 (Hatte-Aelen)	J2 (Bajo-Tithon)	Early Cretaceous	LK1 (Ceno-Sant)	LK2 (Camp-Maast)	Paleocene	Early Eocene
J2 (Bajo-Tithon)	**0.003508**	NA	NA	NA	NA	NA	NA
Early Cretaceous	**0.003131**	0.48788	NA	NA	NA	NA	NA
LK1 (Ceno-Sant)	0.133946	**0.008481**	**0.034769**	NA	NA	NA	NA
LK2 (Camp-Maast)	**0.034338**	0.168577	0.431729	0.382623	NA	NA	NA
Paleocene	0.431729	0.089725	0.089725	0.597384	0.286101	NA	NA
Early Eocene	0.902304	**0.000186**	**0.000258**	0.203337	**0.032357**	0.523821	NA
Extant	0.56262	**2.32E-06**	**6.35E-07**	**0.003508**	**0.003508**	0.775915	0.837841

Early Diapsids

Morphological disparity

```
Original data
```

	Epoch	n	mean	sd	min	Q1	median	Q3	max
1	Cisuralian	100	18.503	0.294	17.583	18.332	18.502	18.694	19.122
2	Guadalupian	100	17.257	0.907	13.908	16.728	17.584	17.962	18.851
3	Lopingian	100	18.372	0.407	17.214	18.108	18.404	18.674	19.109
4	Early Triassic	100	17.657	0.875	13.843	17.121	17.892	18.202	19.320
5	Middle Triassic	100	19.186	0.210	18.710	19.050	19.185	19.316	19.590

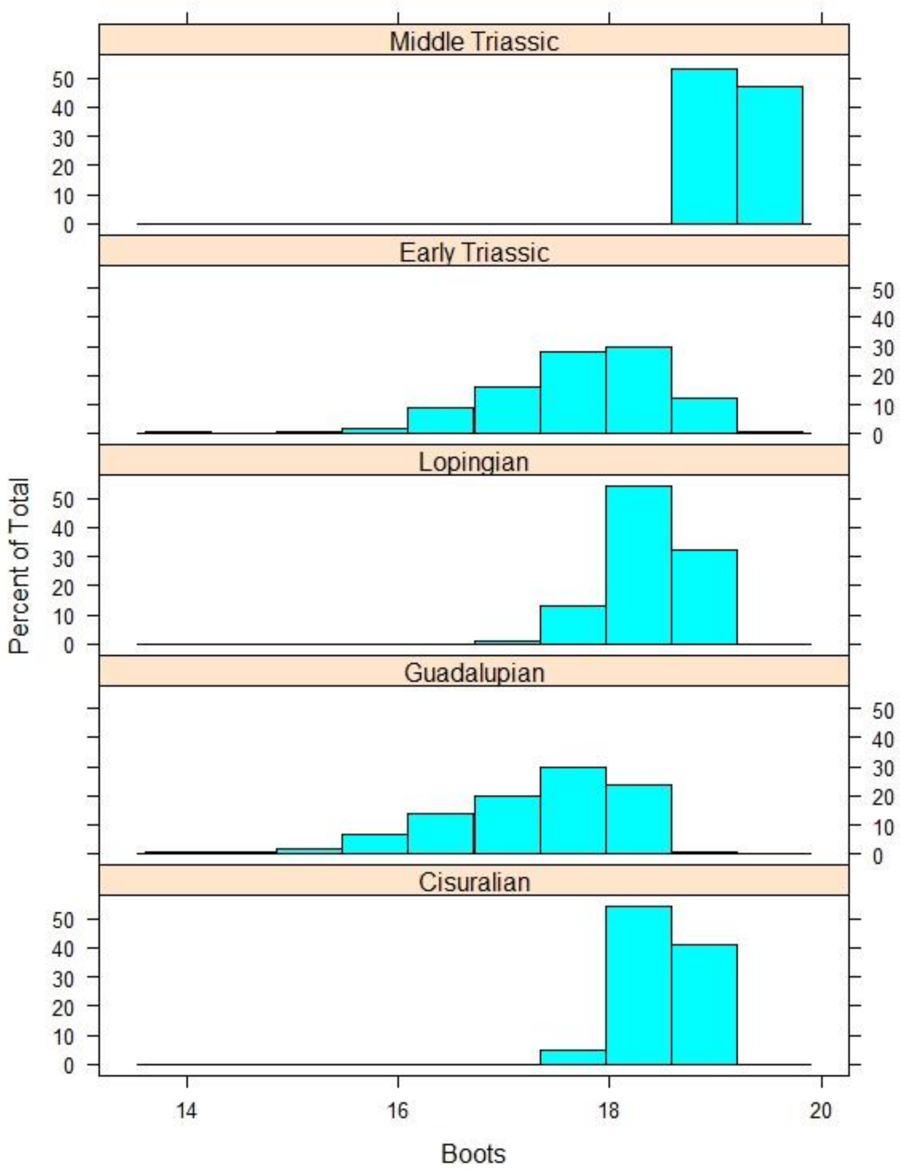

```
$Cisuralian

	Shapiro-Wilk normality test

data:  X[[i]]
W = 0.98648, p-value = 0.4035


$Guadalupian

	Shapiro-Wilk normality test

data:  X[[i]]
W = 0.91876, p-value = 1.211e-05


$Lopingian

	Shapiro-Wilk normality test

data:  X[[i]]
W = 0.98095, p-value = 0.1577


$`Early Triassic`

	Shapiro-Wilk normality test

data:  X[[i]]
W = 0.92468, p-value = 2.515e-05


$`Middle Triassic`

	Shapiro-Wilk normality test

data:  X[[i]]
W = 0.98327, p-value = 0.2368
```

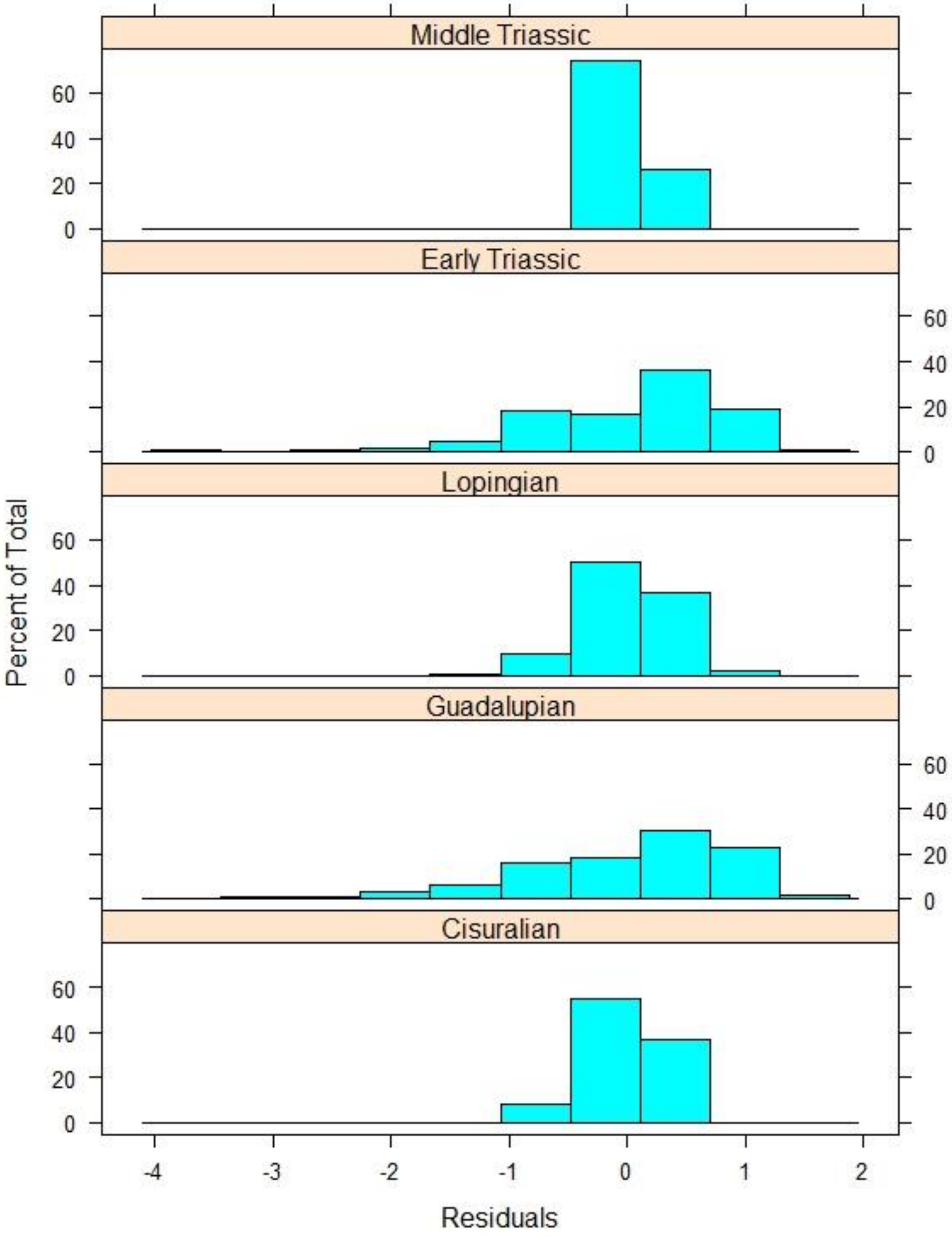

Middle Triassic
Early Triassic
Lopingian
Guadalupian
Cisuralian
Percent of Total
Residuals
0
20
40
60
-4
-3
-2
-1
0
1
2

```
        Shapiro-Wilk normality test BY LEVEL (RESIDUALS)


$Cisuralian

        Shapiro-Wilk normality test

data:  X[[i]]
W = 0.98648, p-value = 0.4035


$Guadalupian

        Shapiro-Wilk normality test

data:  X[[i]]
W = 0.91876, p-value = 1.211e-05


$Lopingian

        Shapiro-Wilk normality test

data:  X[[i]]
W = 0.98095, p-value = 0.1577


$`Early Triassic`

        Shapiro-Wilk normality test

data:  X[[i]]
W = 0.92468, p-value = 2.515e-05


$`Middle Triassic`

        Shapiro-Wilk normality test

data:  X[[i]]
W = 0.98327, p-value = 0.2368
```

Transformed Data (Power of 5)

	Epoch	n	mean	sd	min	Q1	median	Q3	max
1	Cisuralian	100	18.503	0.294	17.583	18.332	18.502	18.694	19.122
2	Guadalupian	100	17.257	0.907	13.908	16.728	17.584	17.962	18.851
3	Lopingian	100	18.372	0.407	17.214	18.108	18.404	18.674	19.109
4	Early Triassic	100	17.657	0.875	13.843	17.121	17.892	18.202	19.320
5	Middle Triassic	100	19.186	0.210	18.710	19.050	19.185	19.316	19.590

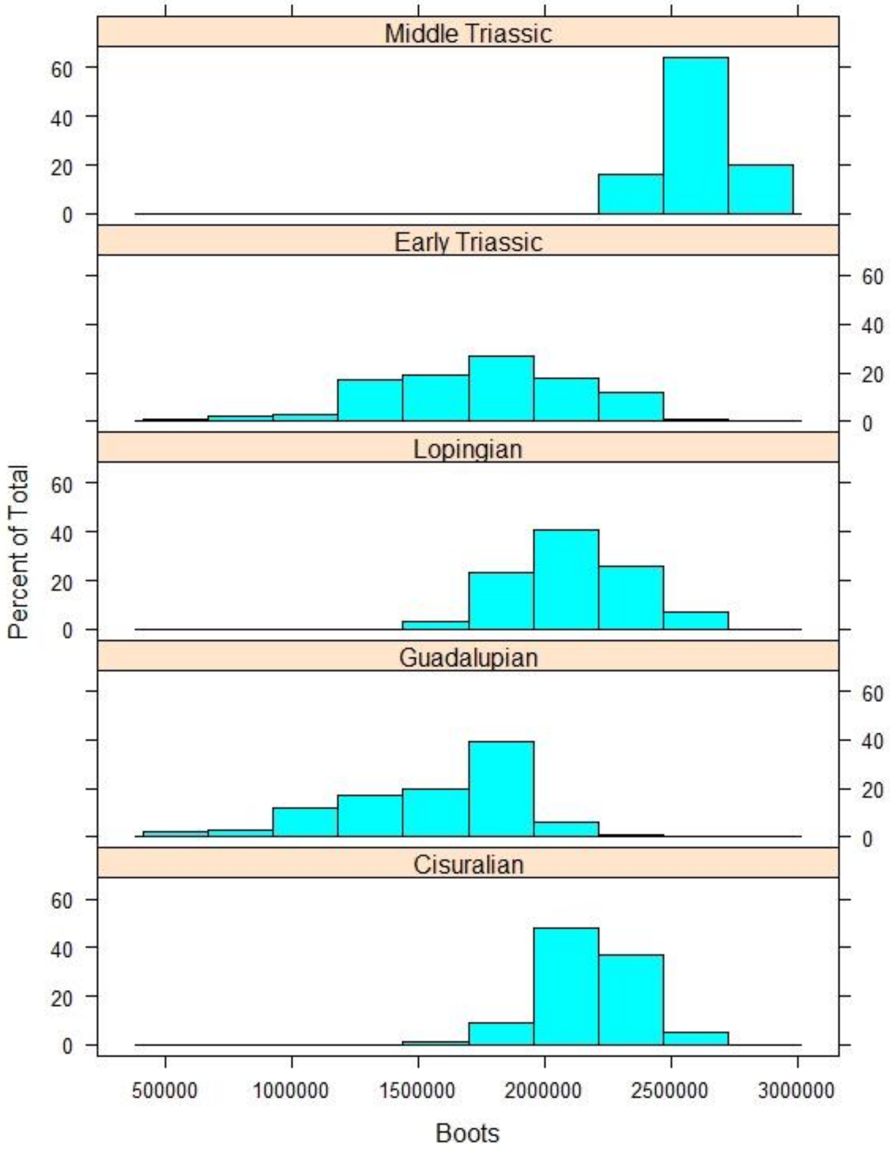

```
        Shapiro-Wilk normality test BY LEVEL (DIsparity)


$Cisuralian

        Shapiro-Wilk normality test

data:  X[[i]]
W = 0.9916, p-value = 0.7913


$Guadalupian

        Shapiro-Wilk normality test

data:  X[[i]]
W = 0.96799, p-value = 0.01555


$Lopingian

        Shapiro-Wilk normality test

data:  X[[i]]
W = 0.98794, p-value = 0.5035


$`Early Triassic`

        Shapiro-Wilk normality test

data:  X[[i]]
W = 0.98107, p-value = 0.1609


$`Middle Triassic`

        Shapiro-Wilk normality test

data:  X[[i]]
W = 0.98318, p-value = 0.2332
```

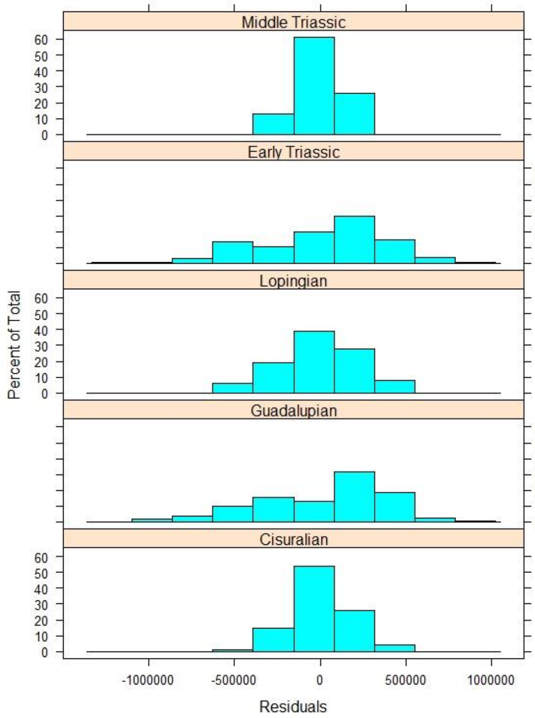
Middle Triassic
Early Triassic
Lopingian
Guadalupian
Cisuralian
Percent of Total
Residuals
-1000000
-500000
0
500000
1000000

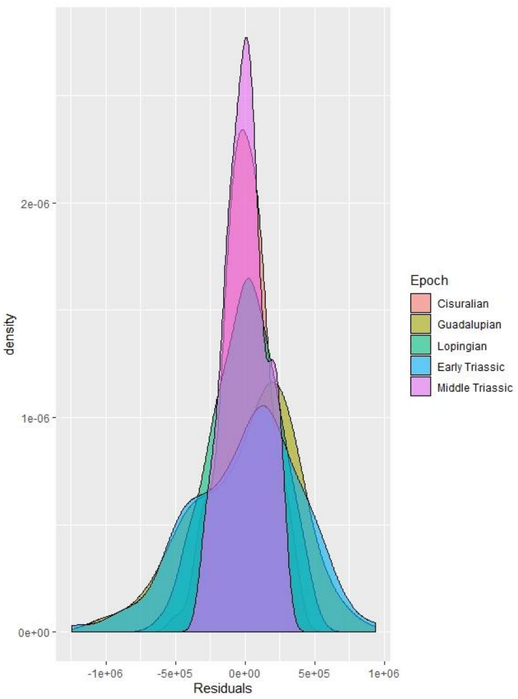
density
Residuals
Epoch
Cisuralian
Guadalupian
Lopingian
Early Triassic
Middle Triassic
2e-06
1e-06
0e+00
-1e+06
-5e+05
0e+00
5e+05
1e+06

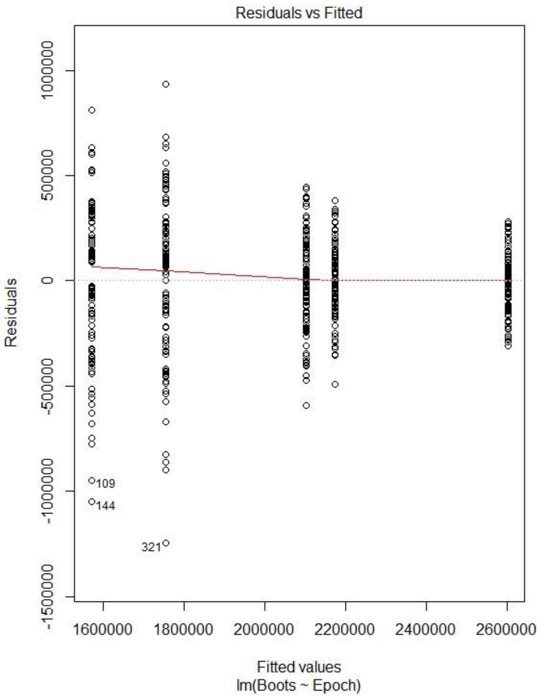
Residuals vs Fitted
Residuals
Fitted values
lm(Boots ~ Epoch)
109
144
321
1000000
500000
0
-500000
-1000000
-1500000
1600000
1800000
2000000
2200000
2400000
2600000

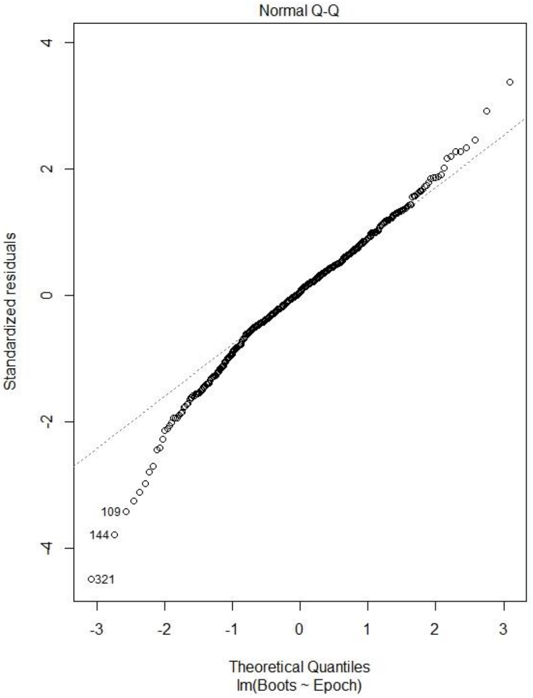
Normal Q-Q
Standardized residuals
Theoretical Quantiles
lm(Boots ~ Epoch)
109
144
321

```
        Shapiro-Wilk normality test BY LEVEL (RESIDUALS)


$Cisuralian

       Shapiro-Wilk normality test

data:  X[[i]]
W = 0.9916, p-value = 0.7913


$Guadalupian

       Shapiro-Wilk normality test

data:  X[[i]]
W = 0.96799, p-value = 0.01555


$Lopingian

       Shapiro-Wilk normality test

data:  X[[i]]
W = 0.98794, p-value = 0.5035


$`Early Triassic`

       Shapiro-Wilk normality test

data:  X[[i]]
W = 0.98107, p-value = 0.1609


$`Middle Triassic`

       Shapiro-Wilk normality test

data:  X[[i]]
W = 0.98318, p-value = 0.2332


Fligner-Killeen test of homogeneity of variances

data:  Residuals by Epoch
Fligner-Killeen:med chi-squared = 80.469, df = 4, p-value < 2.2e-16
```

Decision: Nonparametric test

Pairwise comparisons using Wilcoxon rank sum test

	Cisuralian	Guadalupian	Lopingian	Early Triassic
Guadalupian	**1.60E-27**	NA	NA	NA
Lopingian	**0.026101**	**3.29E-22**	NA	NA
Early Triassic	**1.12E-16**	**0.001066**	**1.54E-11**	NA
Middle Triassic	**1.09E-30**	**3.16E-33**	**1.29E-30**	**3.08E-32**

Lepidosaurs

Morphological disparity

Original data

	Epoch	n	mean	sd	min	Q1	median	Q3	max
1	J1 (Hetta-Aalen)	100	18.052	0.763	15.693	17.650	18.219	18.560	19.162
2	J2 (Bajo-Titho)	100	18.140	0.657	15.827	17.837	18.297	18.585	19.220
3	Early Cretaceous	100	18.259	0.332	17.279	18.010	18.269	18.473	19.025
4	LK1 (Ceno-Sant)	100	18.282	0.706	16.176	17.852	18.386	18.751	19.589
5	LK2 (Camp-Maast)	100	18.912	0.280	17.899	18.706	18.934	19.110	19.485
6	Paleocene	100	17.946	0.777	14.936	17.512	18.090	18.578	19.143
7	Eocene	100	18.872	0.299	17.669	18.726	18.902	19.092	19.419
8	Extant	100	19.965	0.115	19.507	19.908	19.972	20.047	20.188

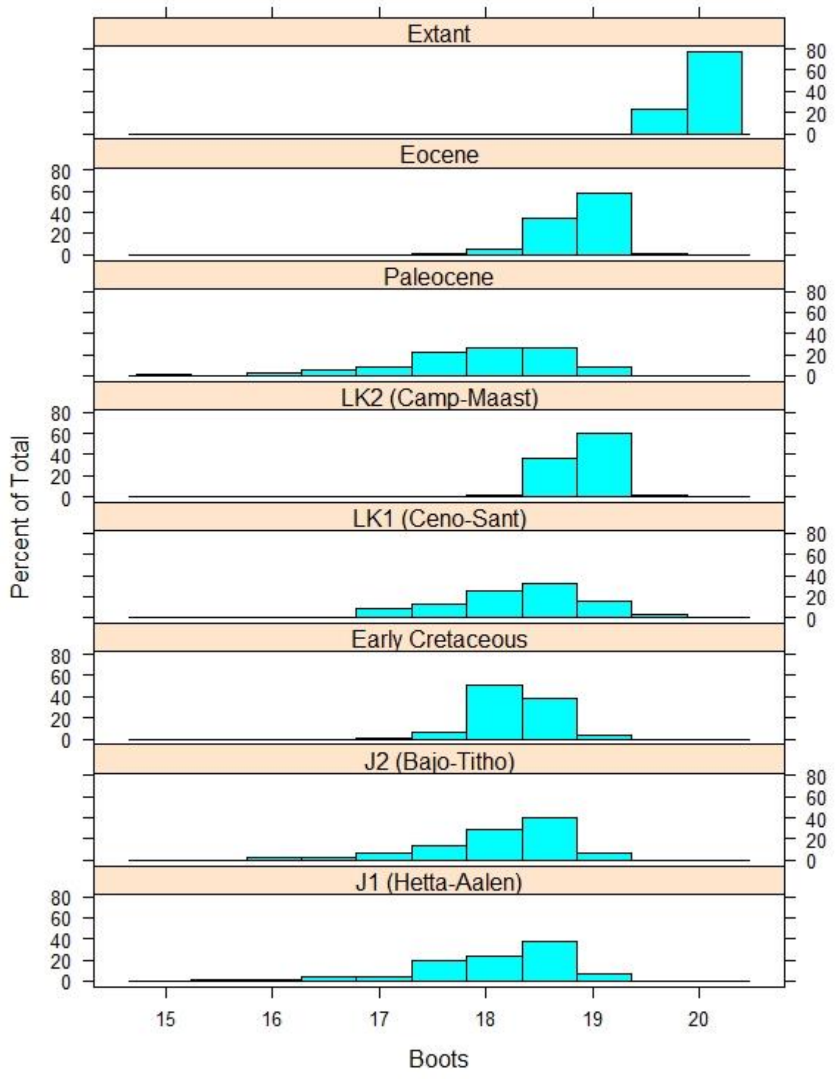

Shapiro-Wilk normality test BY LEVEL (Disparity)

```
$`J1 (Hetta-Aalen)`

	Shapiro-Wilk normality test

data:  X[[i]]
W = 0.90475, p-value = 2.381e-06


$`J2 (Bajo-Titho)`

	Shapiro-Wilk normality test

data:  X[[i]]
W = 0.91657, p-value = 9.296e-06


$`Early Cretaceous`

	Shapiro-Wilk normality test

data:  X[[i]]
W = 0.98822, p-value = 0.5248


$`LK1 (Ceno-Sant)`

	Shapiro-Wilk normality test

data:  X[[i]]
W = 0.97513, p-value = 0.05524


$`LK2 (Camp-Maast)`

	Shapiro-Wilk normality test

data:  X[[i]]
W = 0.97288, p-value = 0.03686


$Paleocene

	Shapiro-Wilk normality test

data:  X[[i]]
W = 0.94152, p-value = 0.0002391


$Eocene

	Shapiro-Wilk normality test

data:  X[[i]]
W = 0.94814, p-value = 0.0006266
```

```
$Extant

        Shapiro-Wilk normality test

data:  X[[i]]
W = 0.95957, p-value = 0.00375
```

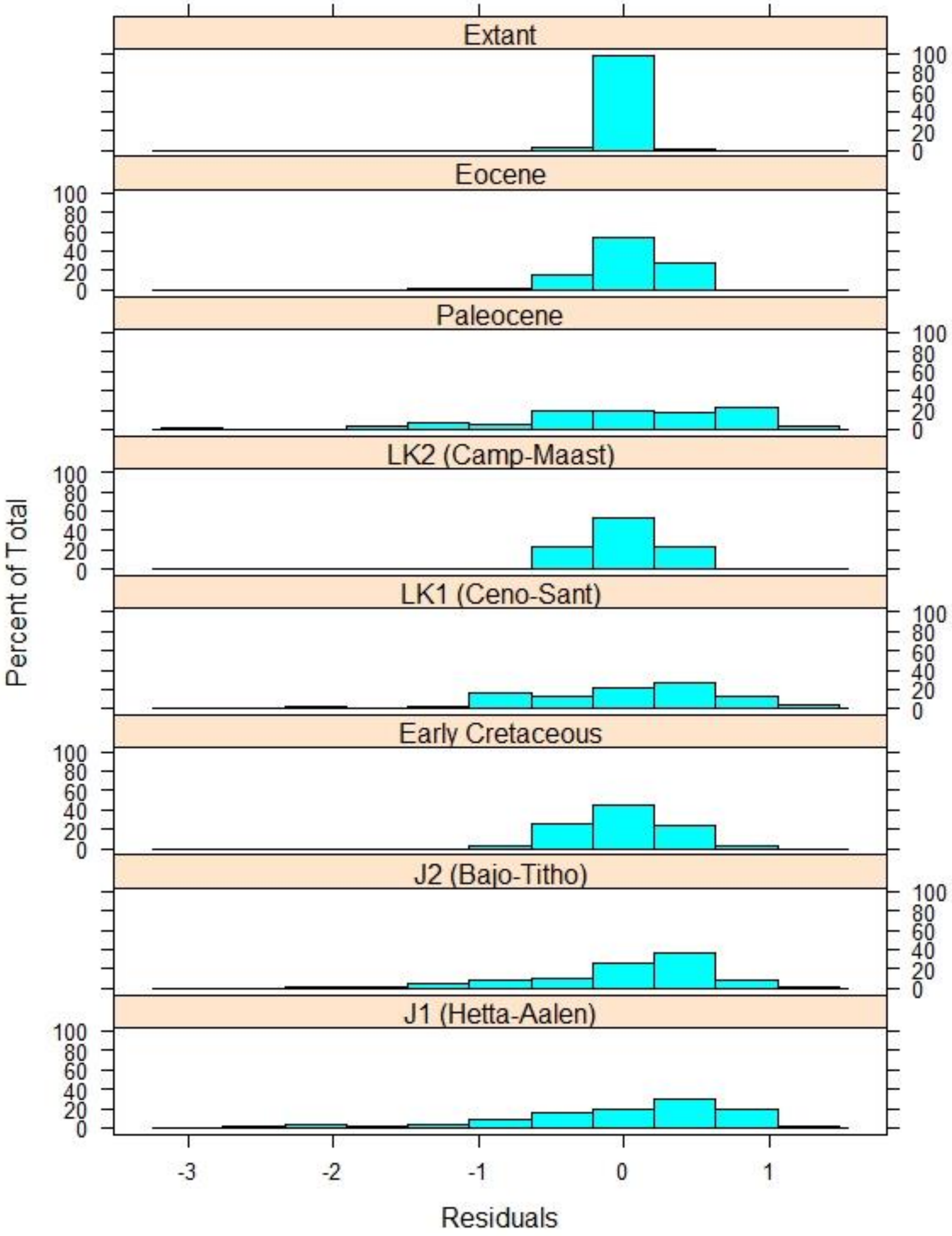


```
 Shapiro-Wilk normality test BY LEVEL (RESIDUALS)
$`J1 (Hetta-Aalen)`

        Shapiro-Wilk normality test
```

```
data:  X[[i]]
W = 0.90475, p-value = 2.381e-06


$`J2 (Bajo-Titho)`

	Shapiro-Wilk normality test

data:  X[[i]]
W = 0.91657, p-value = 9.296e-06


$`Early Cretaceous`

	Shapiro-Wilk normality test

data:  X[[i]]
W = 0.98822, p-value = 0.5248


$`LK1 (Ceno-Sant)`

	Shapiro-Wilk normality test

data:  X[[i]]
W = 0.97513, p-value = 0.05524


$`LK2 (Camp-Maast)`

	Shapiro-Wilk normality test

data:  X[[i]]
W = 0.97288, p-value = 0.03686


$Paleocene

	Shapiro-Wilk normality test

data:  X[[i]]
W = 0.94152, p-value = 0.0002391


$Eocene

	Shapiro-Wilk normality test

data:  X[[i]]
W = 0.94814, p-value = 0.0006266


$Extant

	Shapiro-Wilk normality test

data:  X[[i]]
W = 0.95957, p-value = 0.00375
```

Transformed Data (Power of 5)

	Epoch	n	mean	sd	min	Q1	median	Q3	max
1	J1 (Hetta-Aalen)	100	1949568	373194.9	951907.3	1712901	2007266	2202439	2583719
2	J2 (Bajo-Titho)	100	1988566	331739.7	993241.1	1805603	2050725	2217533	2622674
3	Early Cretaceous	100	2036233	183399.9	1540399.1	1894754	2035207	2150985	2492326
4	LK1 (Ceno-Sant)	100	2071749	384776.0	1107507.2	1813181	2100865	2317769	2884549
5	LK2 (Camp-Maast)	100	2424622	176335.4	1837020.1	2290430	2433467	2548708	2808655
6	Paleocene	100	1894586	378769.5	743225.7	1646767	1937224	2213282	2570791
7	Eocene	100	2399831	184336.9	1722210.8	2302585	2412921	2536576	2761739
8	Extant	100	3173264	90732.1	2824827.5	3127031	3177884	3237975	3353368

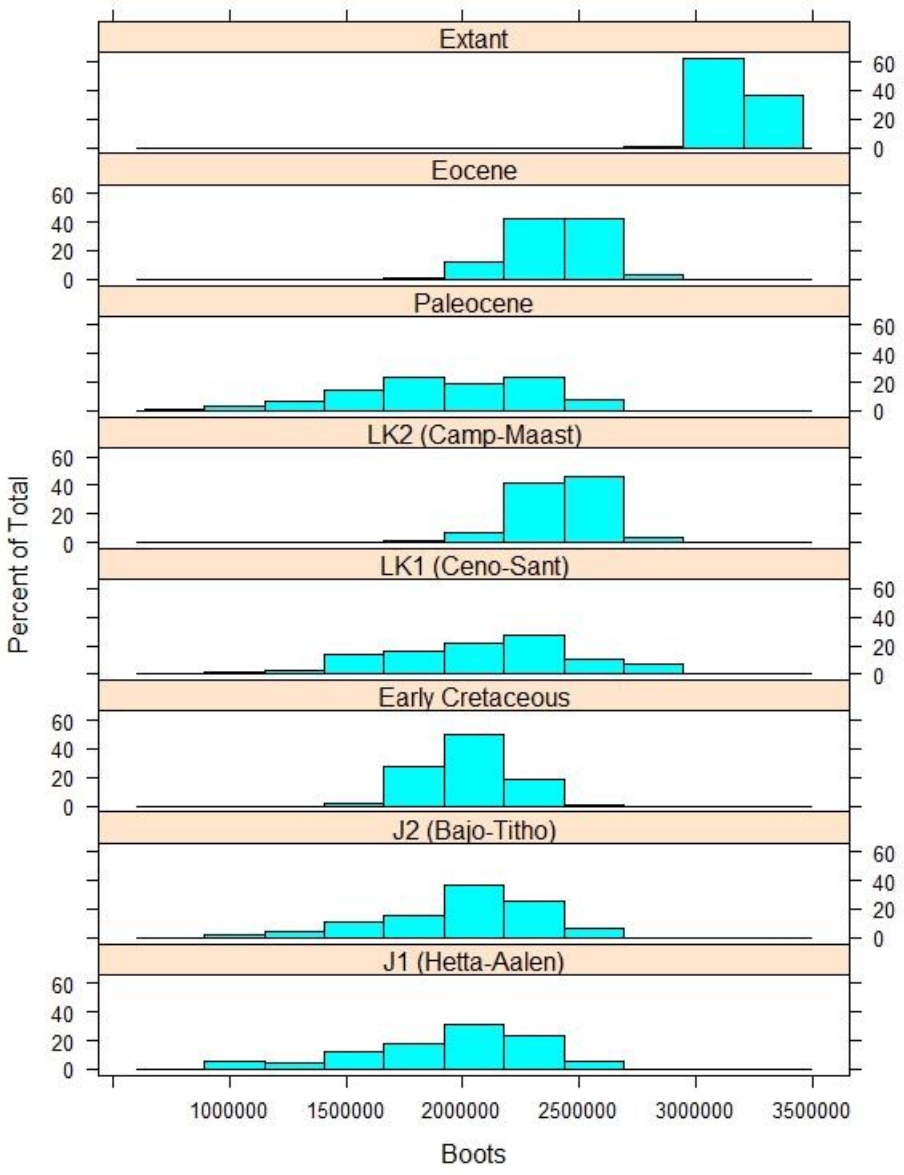

Shapiro-Wilk normality test BY LEVEL (DIsparity)

$`J1 (Hetta-Aalen)`

```
        Shapiro-Wilk normality test

data:  X[[i]]
W = 0.95102, p-value = 0.0009682


$`J2 (Bajo-Titho)`

        Shapiro-Wilk normality test

data:  X[[i]]
W = 0.95845, p-value = 0.003123


$`Early Cretaceous`

        Shapiro-Wilk normality test

data:  X[[i]]
W = 0.99184, p-value = 0.8092


$`LK1 (Ceno-Sant)`

        Shapiro-Wilk normality test

data:  X[[i]]
W = 0.98866, p-value = 0.558


$`LK2 (Camp-Maast)`

        Shapiro-Wilk normality test

data:  X[[i]]
W = 0.98298, p-value = 0.2251


$Paleocene

        Shapiro-Wilk normality test

data:  X[[i]]
W = 0.97582, p-value = 0.06255


$Eocene

        Shapiro-Wilk normality test

data:  X[[i]]
W = 0.96718, p-value = 0.0135


$Extant

        Shapiro-Wilk normality test

data:  X[[i]]
W = 0.96506, p-value = 0.009384
```

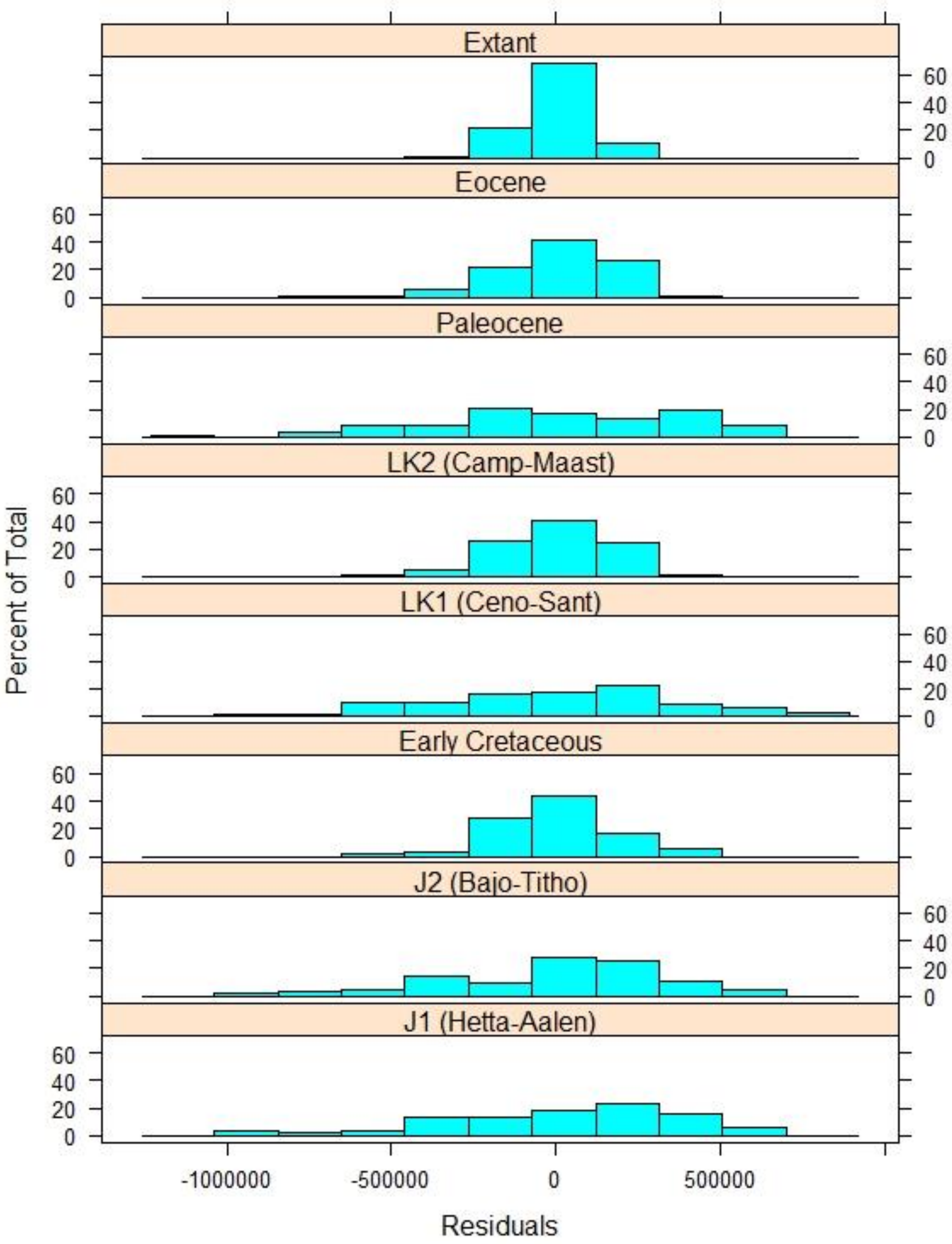


```
            Shapiro-Wilk normality test BY LEVEL (RESIDUALS)
$`J1 (Hetta-Aalen)`
```

```
        Shapiro-Wilk normality test

data:  X[[i]]
W = 0.95102, p-value = 0.0009682


$`J2 (Bajo-Titho)`

        Shapiro-Wilk normality test

data:  X[[i]]
W = 0.95845, p-value = 0.003123


$`Early Cretaceous`

        Shapiro-Wilk normality test

data:  X[[i]]
W = 0.99184, p-value = 0.8092


$`LK1 (Ceno-Sant)`

        Shapiro-Wilk normality test

data:  X[[i]]
W = 0.98866, p-value = 0.558


$`LK2 (Camp-Maast)`

        Shapiro-Wilk normality test

data:  X[[i]]
W = 0.98298, p-value = 0.2251


$Paleocene

        Shapiro-Wilk normality test

data:  X[[i]]
W = 0.97582, p-value = 0.06255


$Eocene

        Shapiro-Wilk normality test

data:  X[[i]]
W = 0.96718, p-value = 0.0135


$Extant

        Shapiro-Wilk normality test

data:  X[[i]]
W = 0.96506, p-value = 0.009384

        Fligner-Killeen test of homogeneity of variances
```

```
data:  Residuals by Epoch
Fligner-Killeen:med chi-squared = 173.34, df = 7, p-value < 2.2e-16
```

Decision: Nonparametric test

Pairwise comparisons using Wilcoxon rank sum test

	J1 (Hetta-Aalen)	J2 (Bajo-Titho)	Early Cretaceous	LK1 (Ceno-Sant)	LK2 (Camp-Maast)	Paleocene	Eocene
J2 (Bajo-Titho)	0.681771	NA	NA	NA	NA	NA	NA
Early Cretaceous	0.431259	0.925053	NA	NA	NA	NA	NA
LK1 (Ceno-Sant)	0.08743	0.19504	0.312467	NA	NA	NA	NA
LK2 (Camp-Maast)	**1.52E-21**	**1.08E-21**	**9.83E-26**	**1.20E-12**	NA	NA	NA
Paleocene	0.291441	0.102794	**0.017518**	**0.006147**	**1.01E-22**	NA	NA
Eocene	**3.06E-20**	**3.06E-20**	**1.10E-23**	**1.39E-11**	0.438097	**1.66E-21**	NA
Extant	**1.12E-33**	**1.12E-33**	**1.12E-33**	**1.12E-33**	**1.12E-33**	**1.12E-33**	**1.12E-33**
